# Supplementary material for: Revealing the structures of megadalton-scale DNA complexes with nucleotide resolution
Source: Nat Commun. 2020 Dec 4;11:6229. doi: 10.1038/s41467-020-20020-7 (PMC7718922; doi:10.1038/s41467-020-20020-7)
Supplement: Supplementary file 1 — Supplementary Information [file 41467_2020_20020_MOESM1_ESM.pdf]

Supplementary Information:

Revealing the structures of megadalton-scale DNA complexes with nucleotide resolution

Massimo Kube*[1], Fabian Kohler*[1], Elija Feigl*[1], Baki Nagel-Yüksel[1], Elena M. Willner[1], Jonas Funke[1], Thomas Gerling[1], Pierre Stömmer[1], Maximilian N. Honemann[1], Thomas G. Martin[2], Sjors H.W. Scheres[2], Hendrik Dietz[1#]

[1] Physik Department, Technische Universität München, Garching near Munich, Germany
[2] MRC Laboratory of Molecular Biology, Cambridge, United Kingdom
* These authors contributed equally. #Correspondence to dietz@tum.de

Content

Supplementary Figures 1-63
Supplementary Tables 1-3

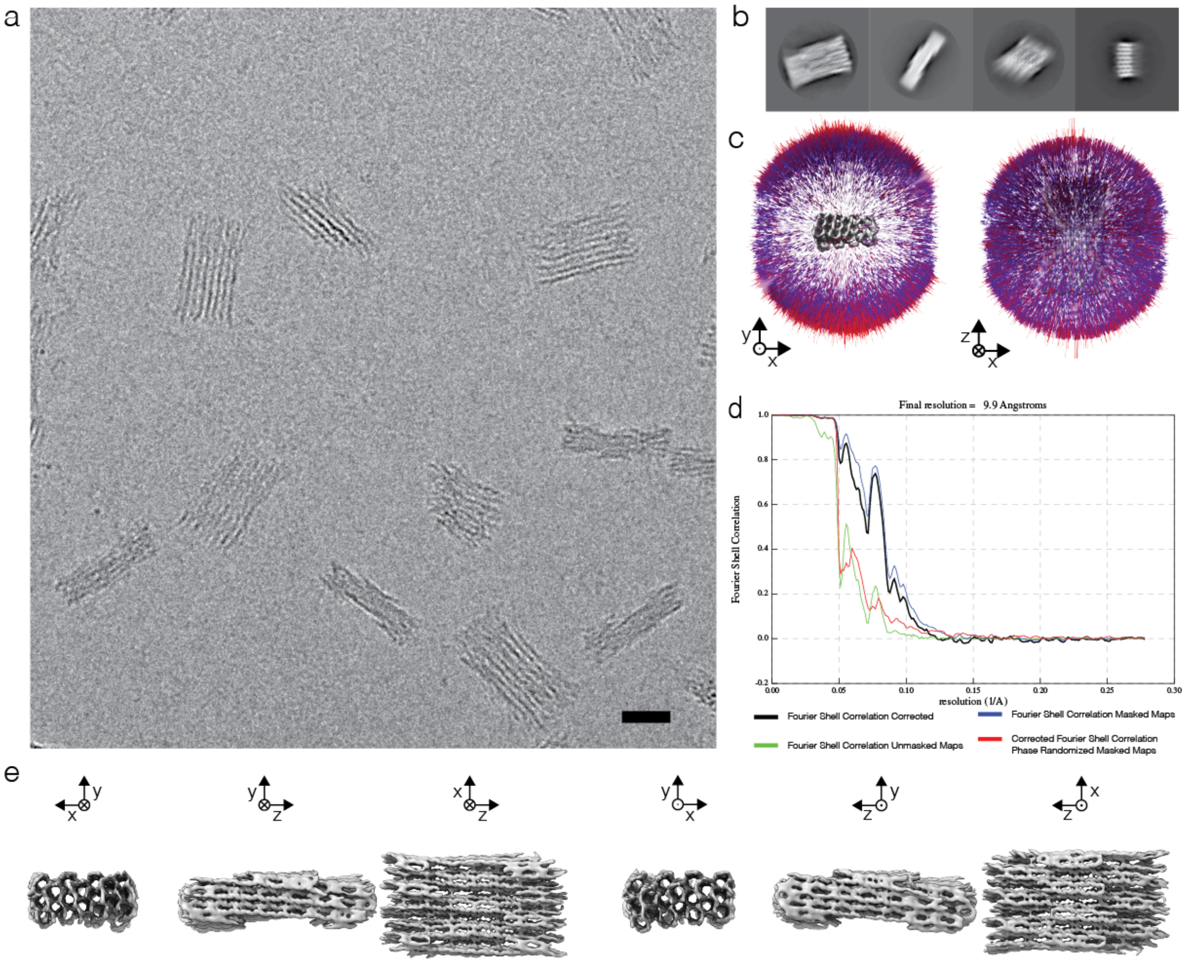

Supplementary Figure 1 | Cryo-EM map determination for 48 helix bundle brick. (a) Exemplary micrograph of a total of 1491 movie stacks. Scale bar = 25 nm. (b) Representative 2D Class averages. (c) Histogram representing the orientational distribution of particles. (d) FSC plot. (e) Six different views of the electron density map.

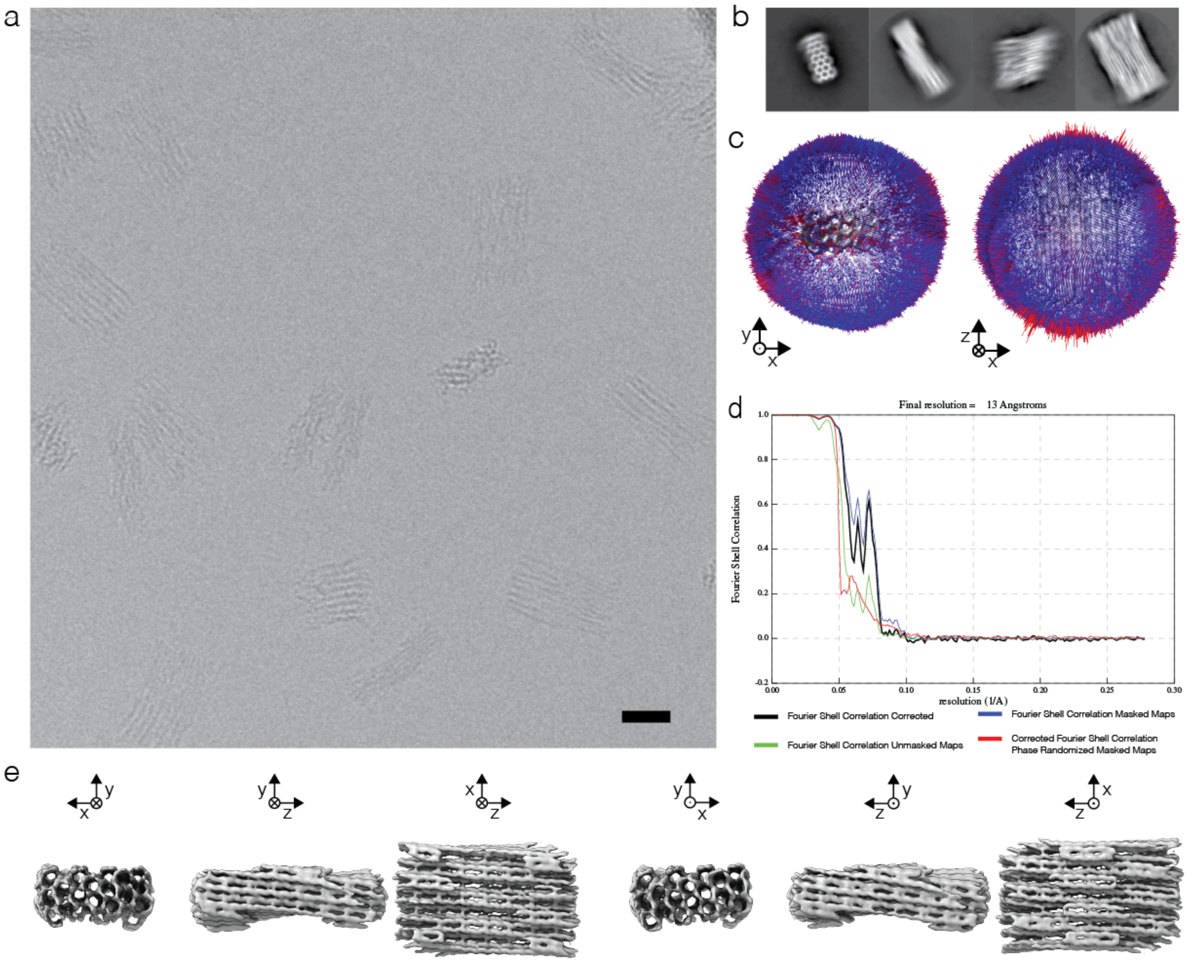

Supplementary Figure 2 | Cryo-EM map determination for 48 helix bundle brick with one T at all staple crossovers. (a) Exemplary micrograph of a total of 3893 movie stacks. Scale bar = 25 nm. (b) Representative 2D Class averages. (c) Histogram representing the orientational distribution of particles. (d) FSC plot. (e) Six different views of the electron density map.

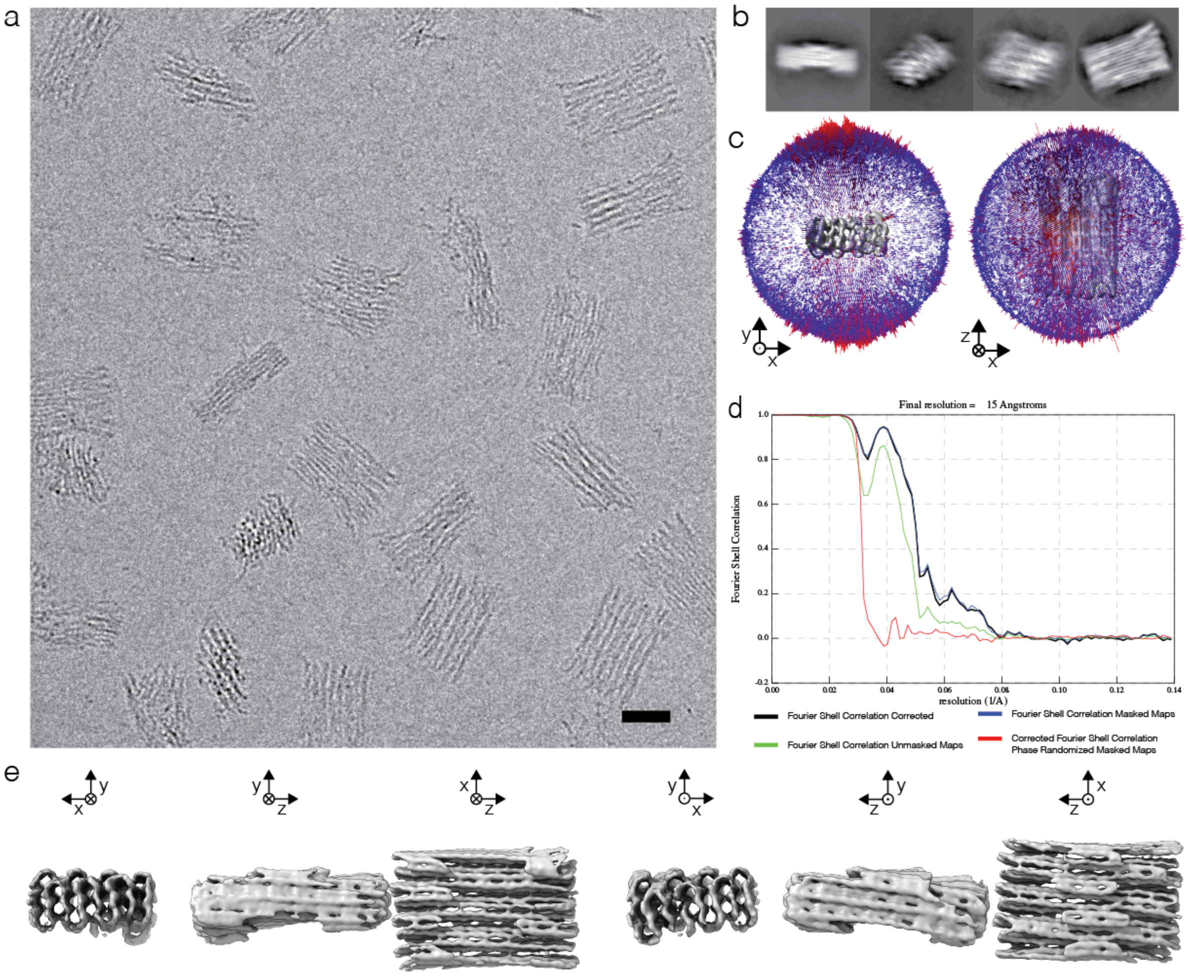

Supplementary Figure 3 | Cryo-EM map determination for 48 helix bundle brick with two T's at all staple crossovers. (a) Exemplary micrograph of a total of 2600 movie stacks. Scale bar = 25 nm. (b) Representative 2D Class averages. (c) Histogram representing the orientational distribution of particles. (d) FSC plot. (e) Six different views of the electron density map.

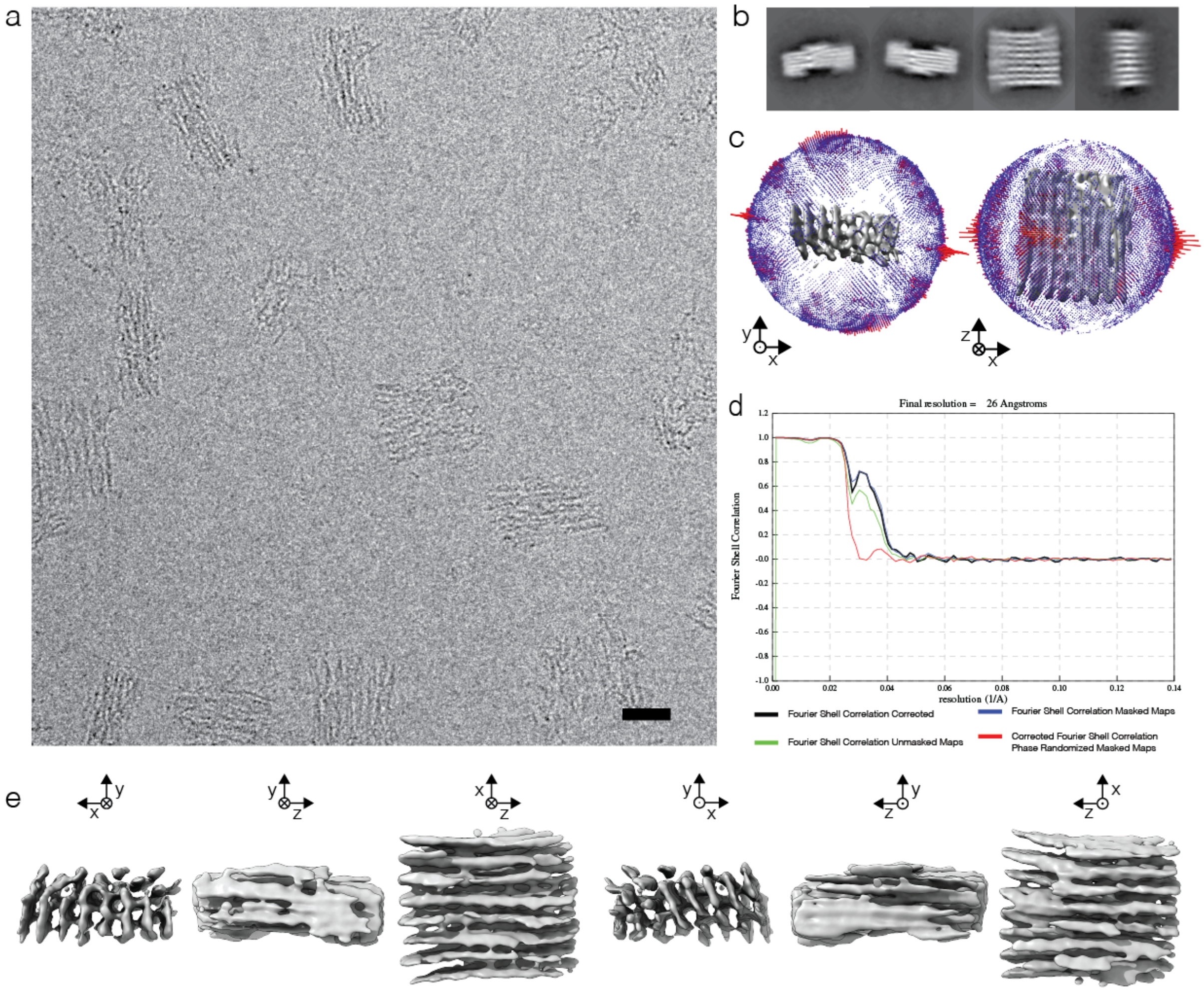

Supplementary Figure 4 | Cryo-EM map determination for 48 helix bundle brick with four T's at all staple crossovers. (a) Exemplary micrograph of a total of 1639 movie stacks. Scale bar = 25 nm. (b) Representative 2D Class averages. (c) Histogram representing the orientational distribution of particles. (d) FSC plot. (e) Six different views of the electron density map.

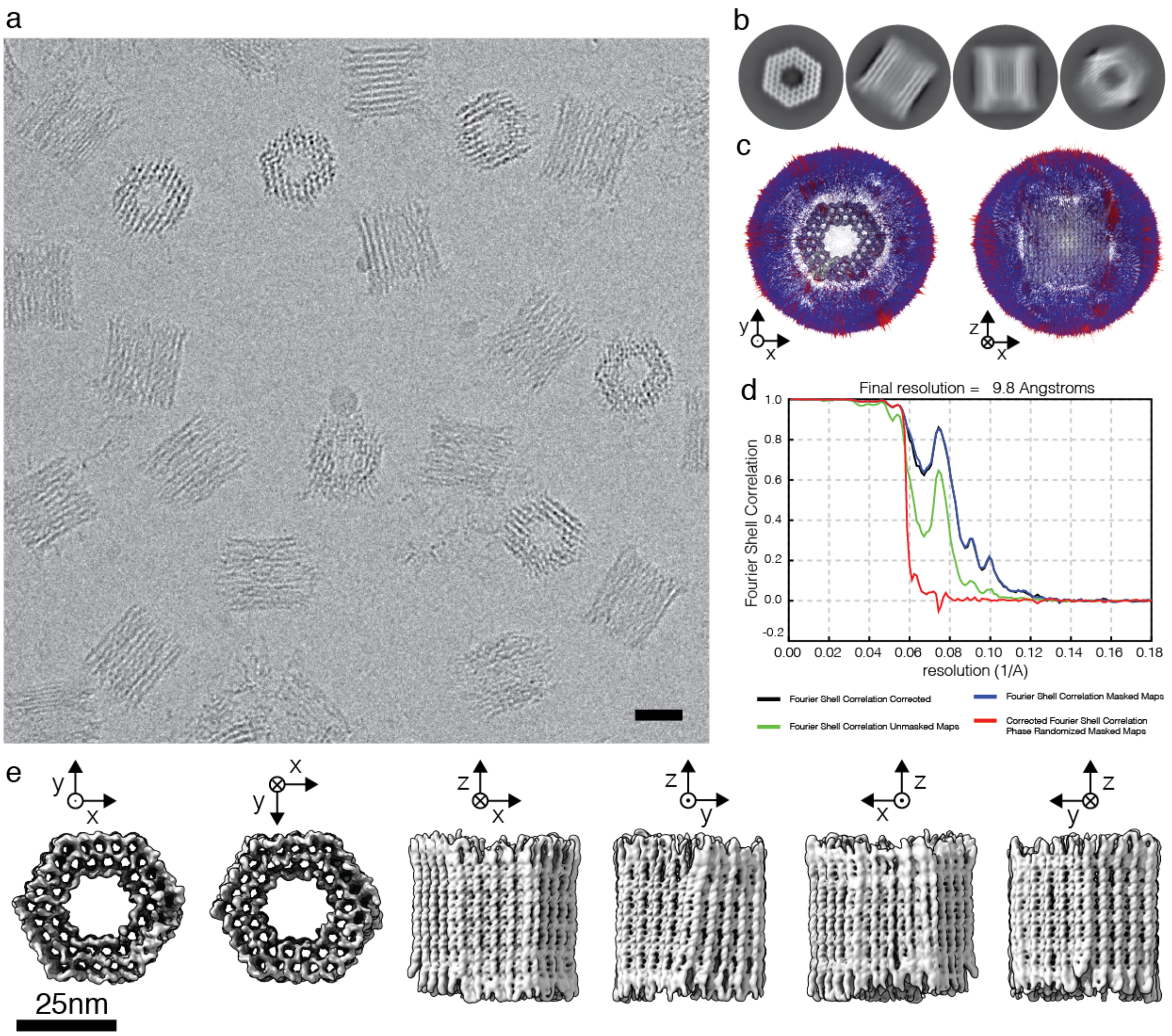

Supplementary Figure 5 | Cryo-EM map determination for the 126-helix barrel. (a) Exemplary micrograph of a total of 5005 movie stacks. Scale bar = 25 nm. (b) Representative 2D Class averages. (c) FSC plot. (d) Histogram representing the orientational distribution of particles. (e) Six different views of the electron density map.

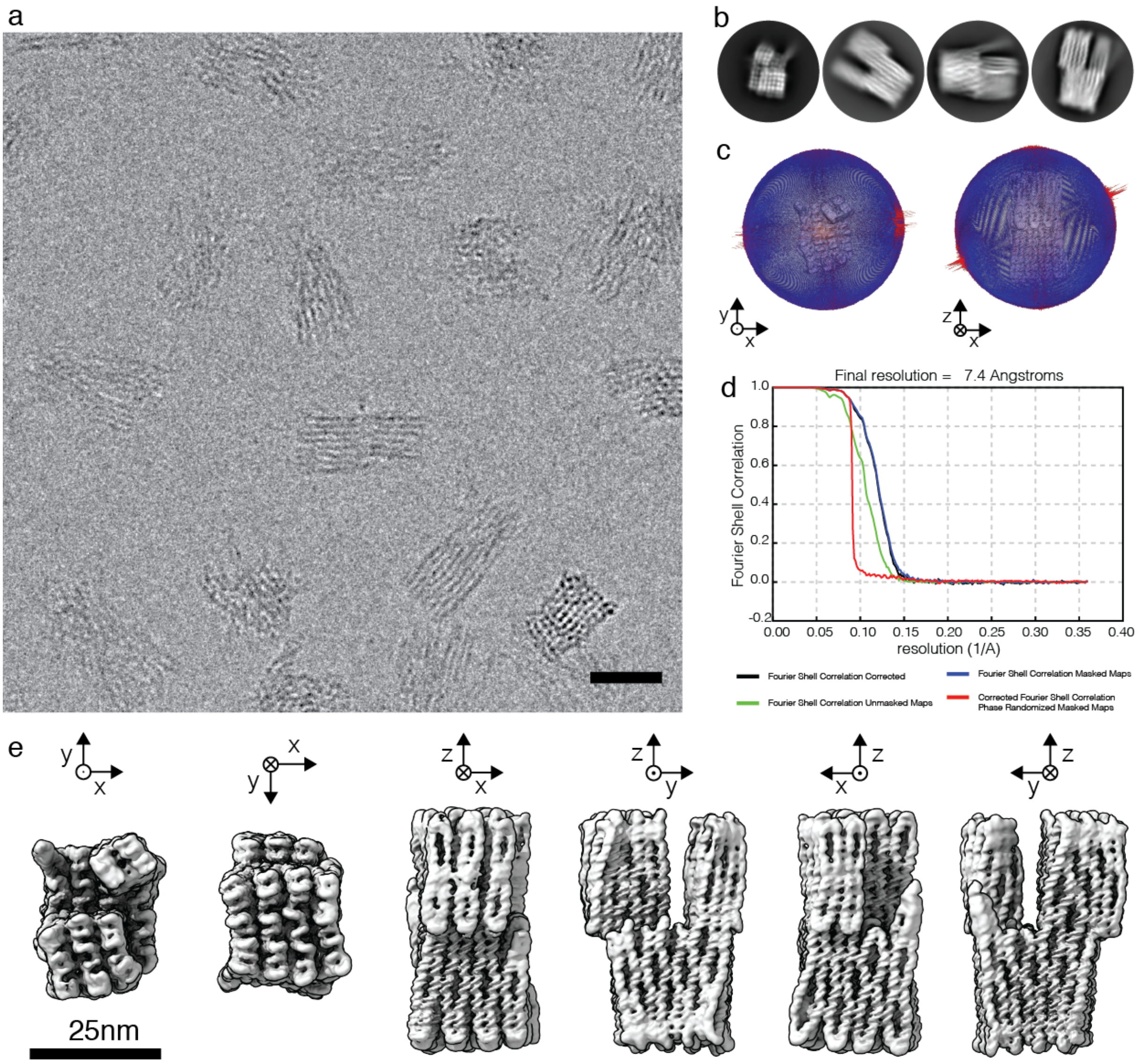

Supplementary Figure 6: Cryo-EM map determination for the Twisttower. (a) Exemplary micrograph of a total of 19434 movie stacks. Scale bar = 25 nm. (b) Representative 2D Class averages. (c) FSC plot. (d) Histogram representing the orientational distribution of particles. (e) Six different views of the electron density map.

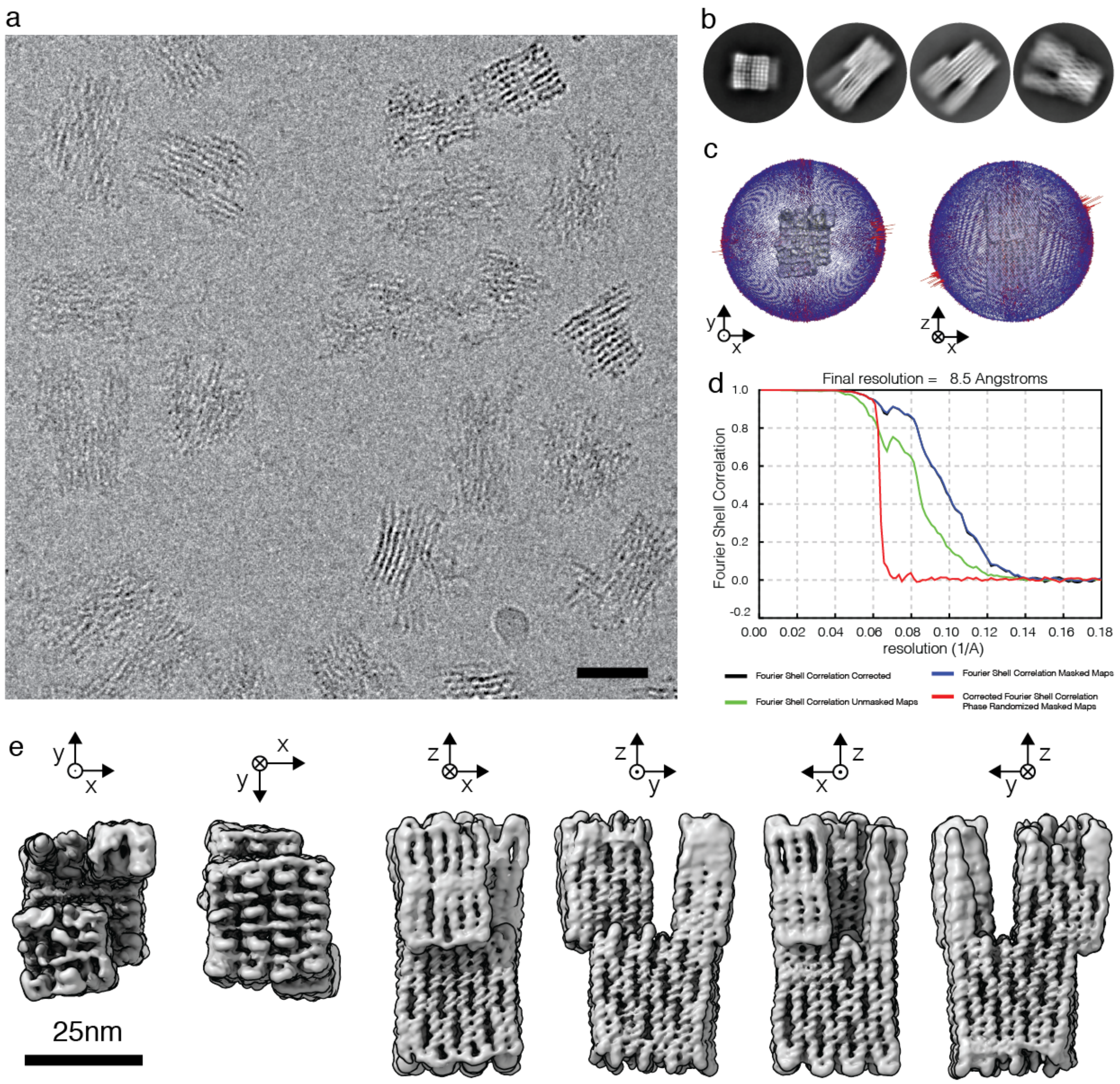

Supplementary Figure 7 | Cryo-EM map determination for the twist-corrected Twisttower. (a) Exemplary micrograph of a total of 5911 movie stacks. Scale bar = 25 nm. (b) Representative 2D Class averages. (c) FSC plot. (d) Histogram representing the orientational distribution of particles. (e) Six different views of the electron density map.

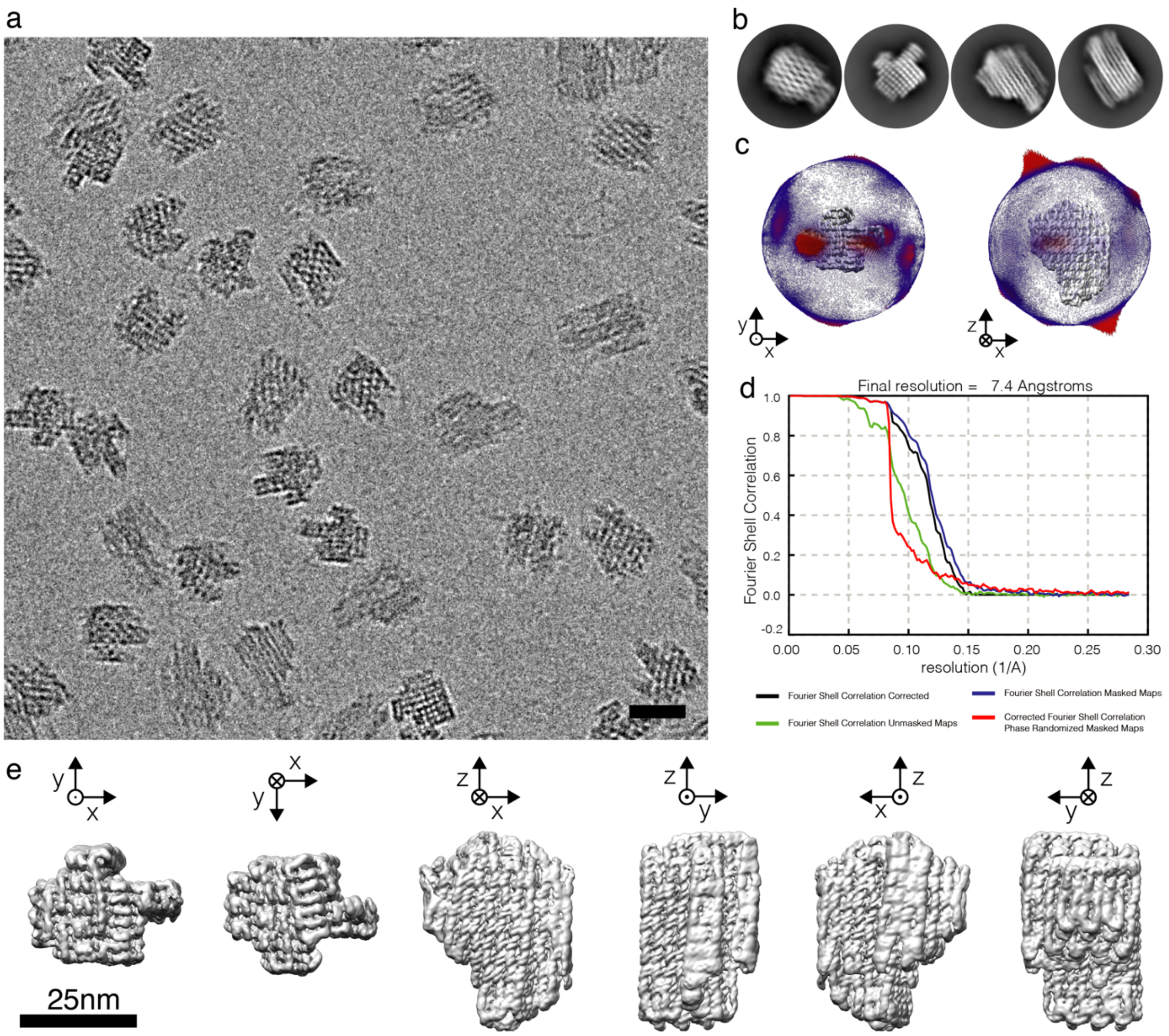

Supplementary Figure 8 | Cryo-EM map determination for pointer v2 object. (a) Exemplary micrograph of a total of 2381 movie stacks. Scale bar = 25 nm. (b) Representative 2D Class averages. (c) Histogram representing the orientational distribution of particles. (d) FSC plot. (e) Six different views of the electron density map.

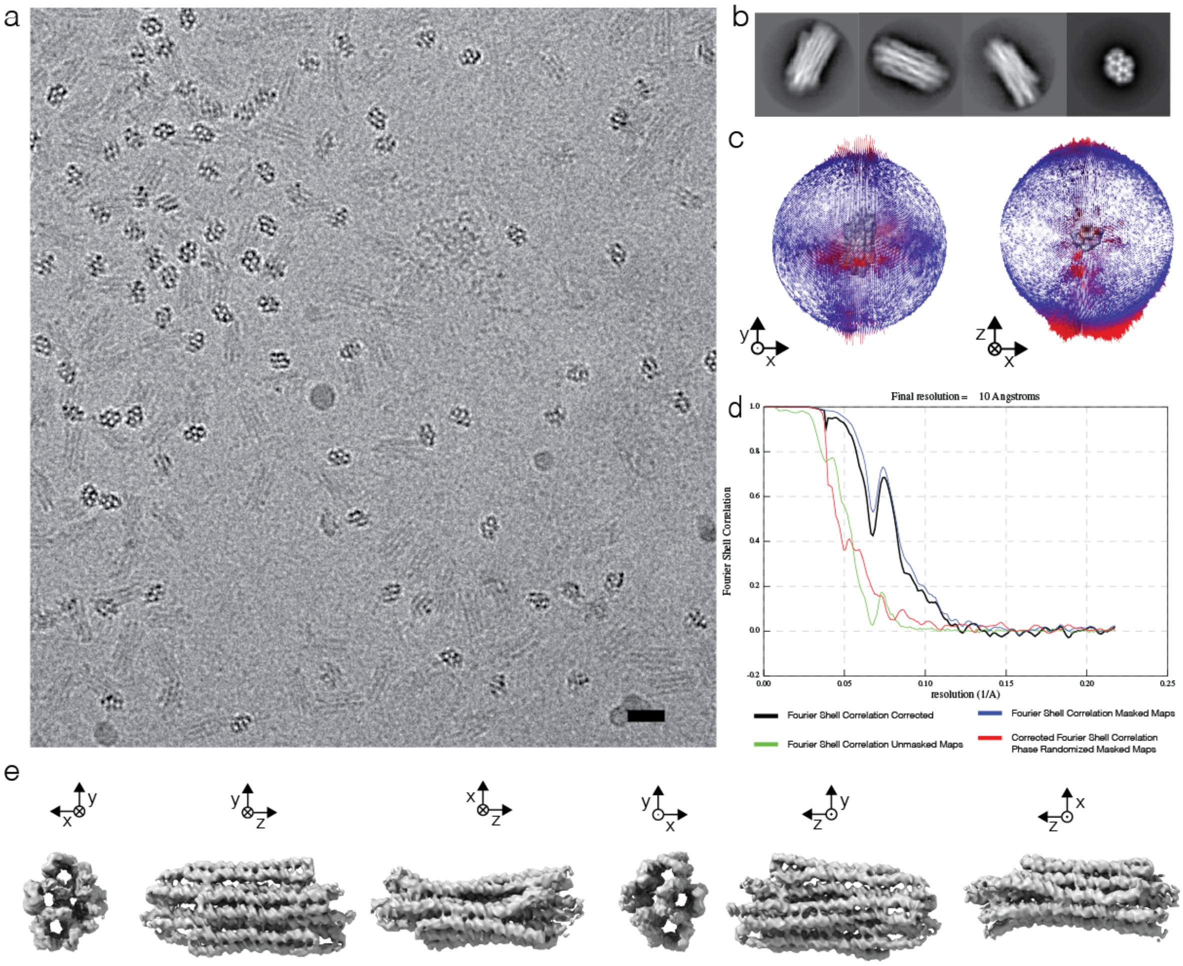

Supplementary Figure 9 | Cryo-EM map determination for 16-helix-bundle. (a) Exemplary micrograph of a total of 480 movie stacks. Scale bar = 25 nm. (b) Representative 2D Class averages. (c) Histogram representing the orientational distribution of particles. (d) FSC plot. (e) Six different views of the electron density map.

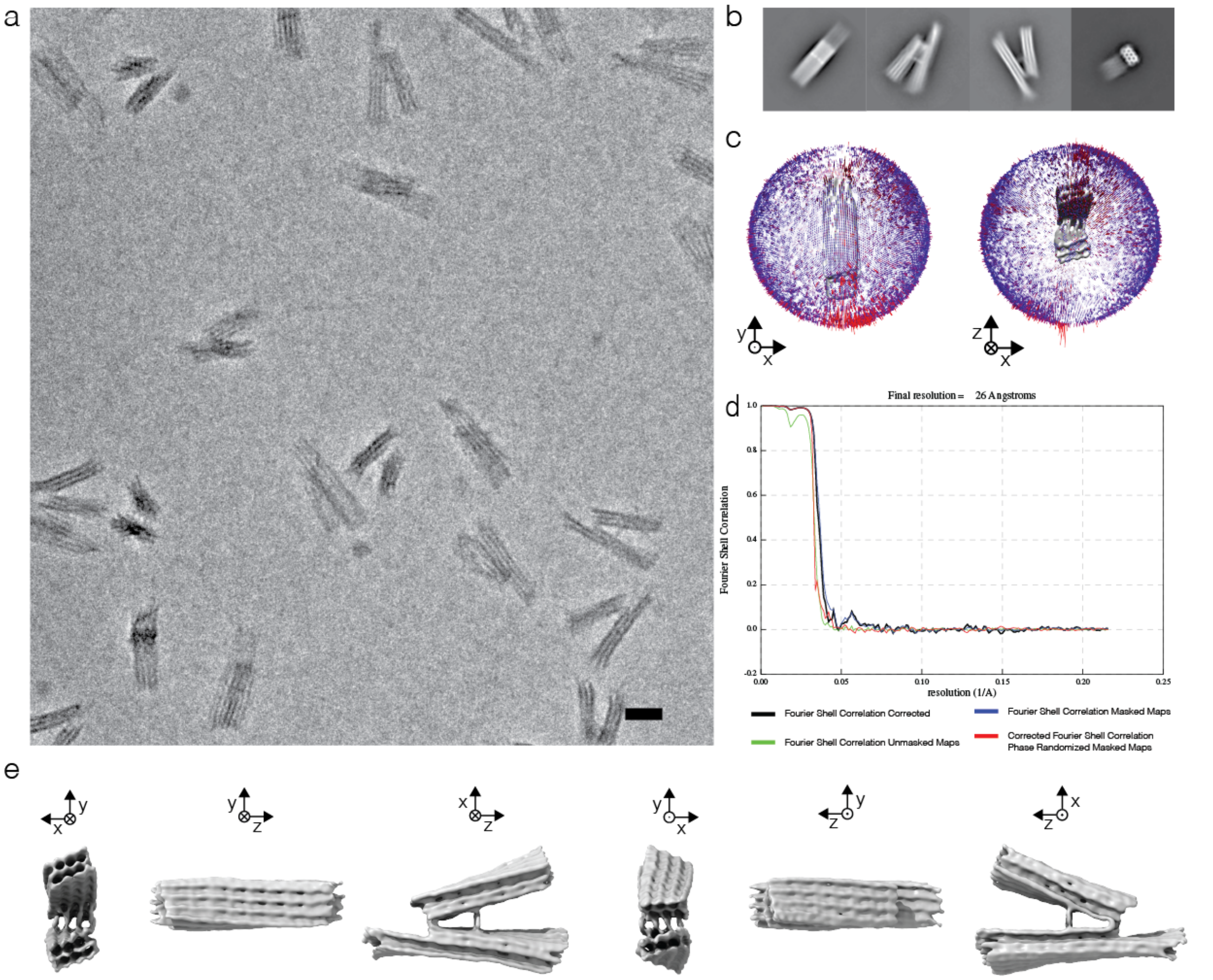

Supplementary Figure 10 | Cryo EM map determination for the hinged beam object v1. (a) Exemplary micrograph of a total of 744 movie stacks. Scale bar = 25 nm. (b) Representative 2D Class averages. (c) Histogram representing the orientational distribution of particles. (d) FSC plot. (e) Six different views of the electron density map.

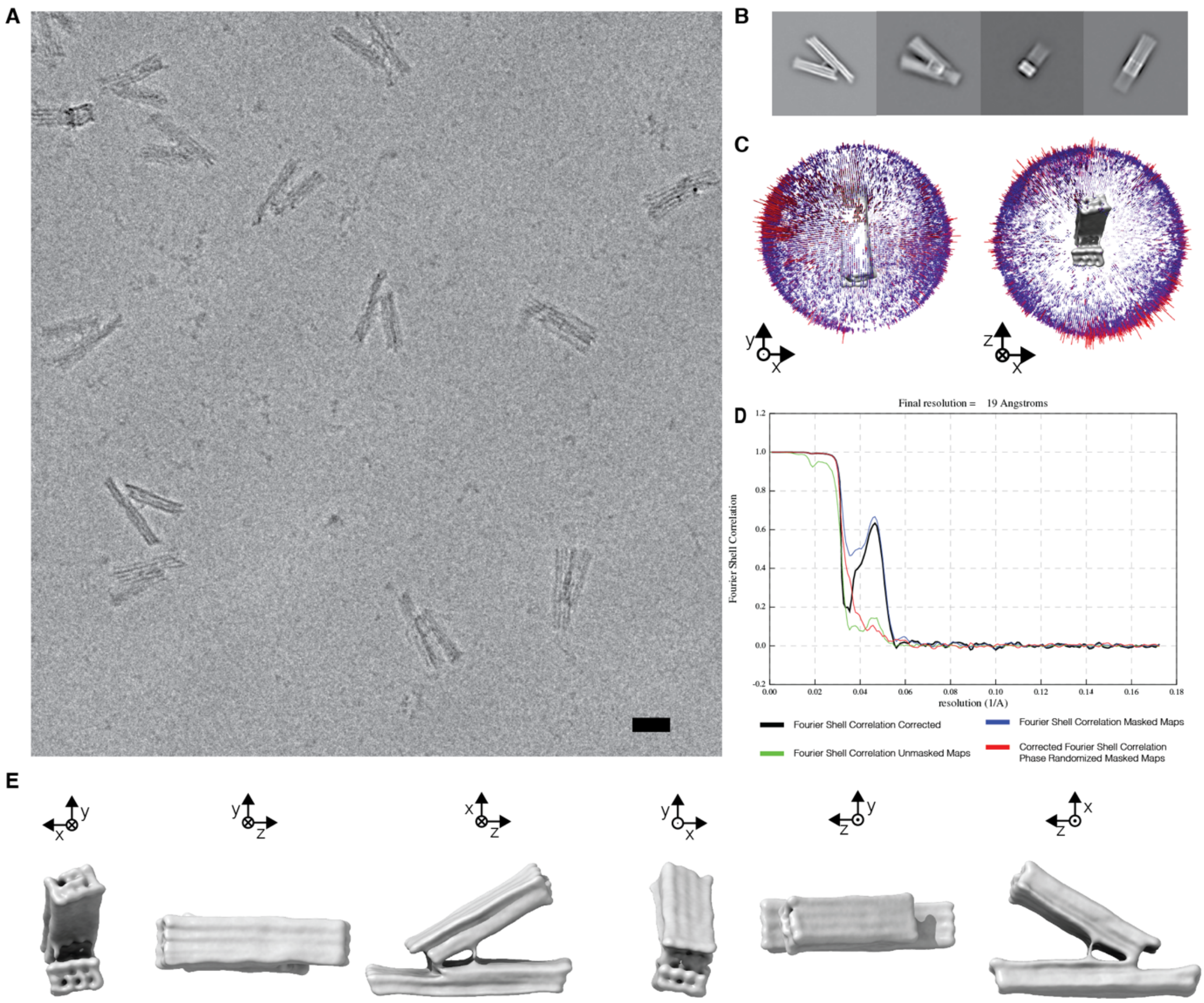

Supplementary Figure 11 | Cryo-EM map determination for the hinged beam object v2. (a) Exemplary micrograph of a total of 989 movie stacks. Scale bar = 25 nm. (b) Representative 2D Class averages. (c) Histogram representing the orientational distribution of particles. (d) FSC plot. (e) Six different views of the electron density map.

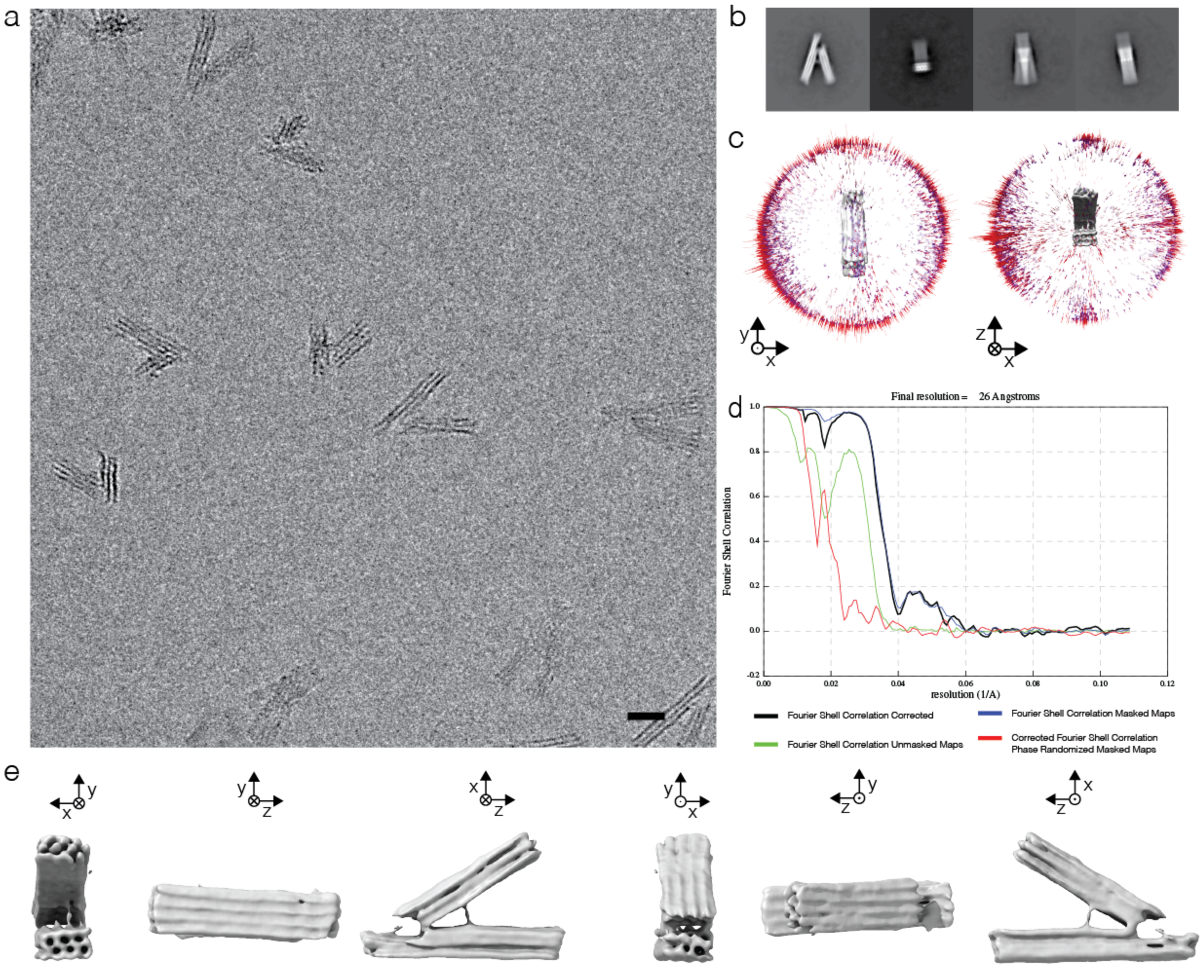

Supplementary Figure 12 | Cryo-EM map determination for the hinged beam object v3. (a) Exemplary micrograph of a total of 358 movie stacks. Scale bar = 25 nm. (b) Representative 2D Class averages. (c) Histogram representing the orientational distribution of particles. (d) FSC plot. (e) Six different views of the electron density map.

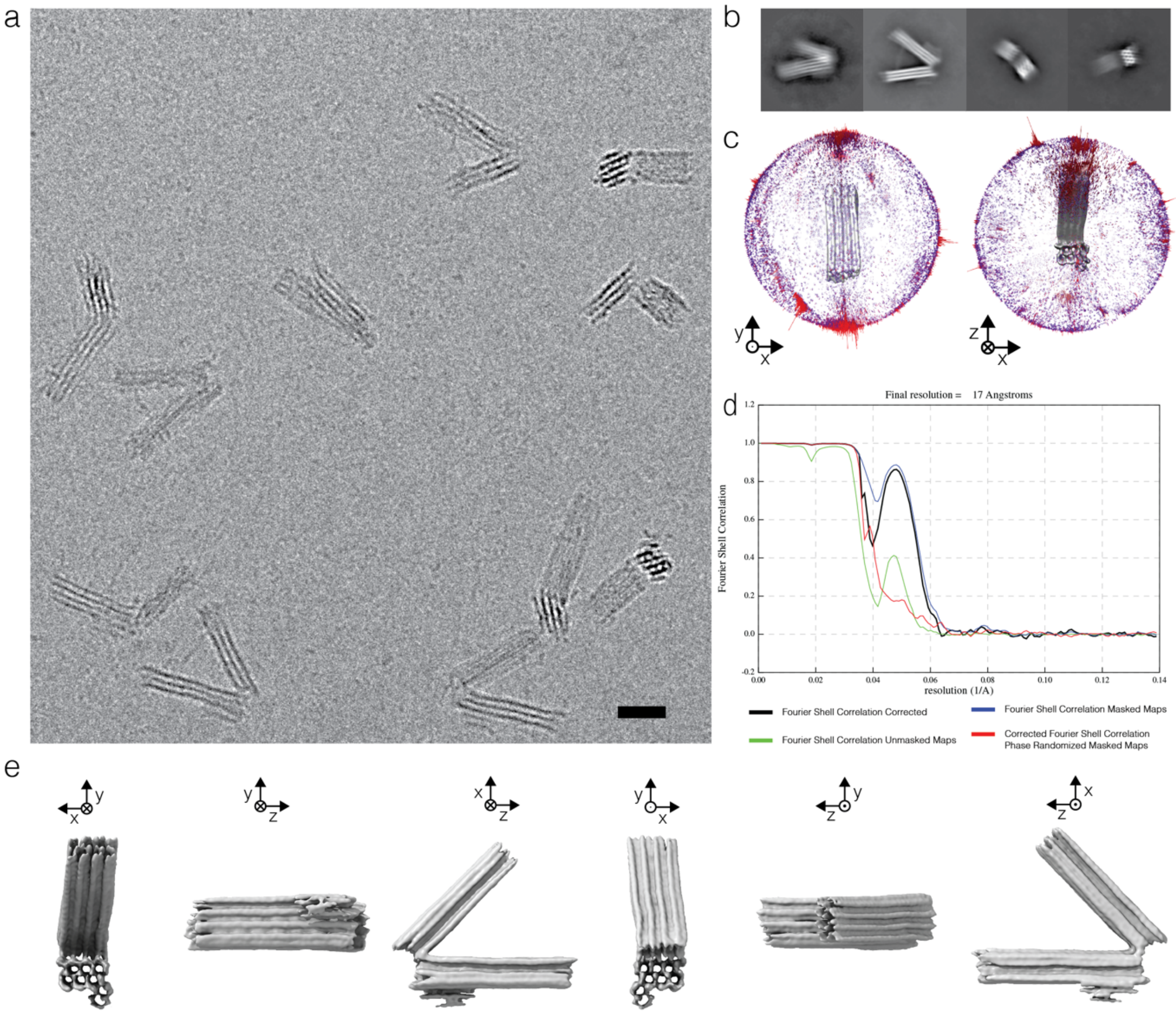

Supplementary Figure 13 | Cryo-EM map determination for the hinged beam object v4. (a) Exemplary micrograph of a total of 2503 movie stacks. Scale bar = 25 nm. (b) Representative 2D Class averages. (c) Histogram representing the orientational distribution of particles. (d) FSC plot. (e) Six different views of the electron density map.

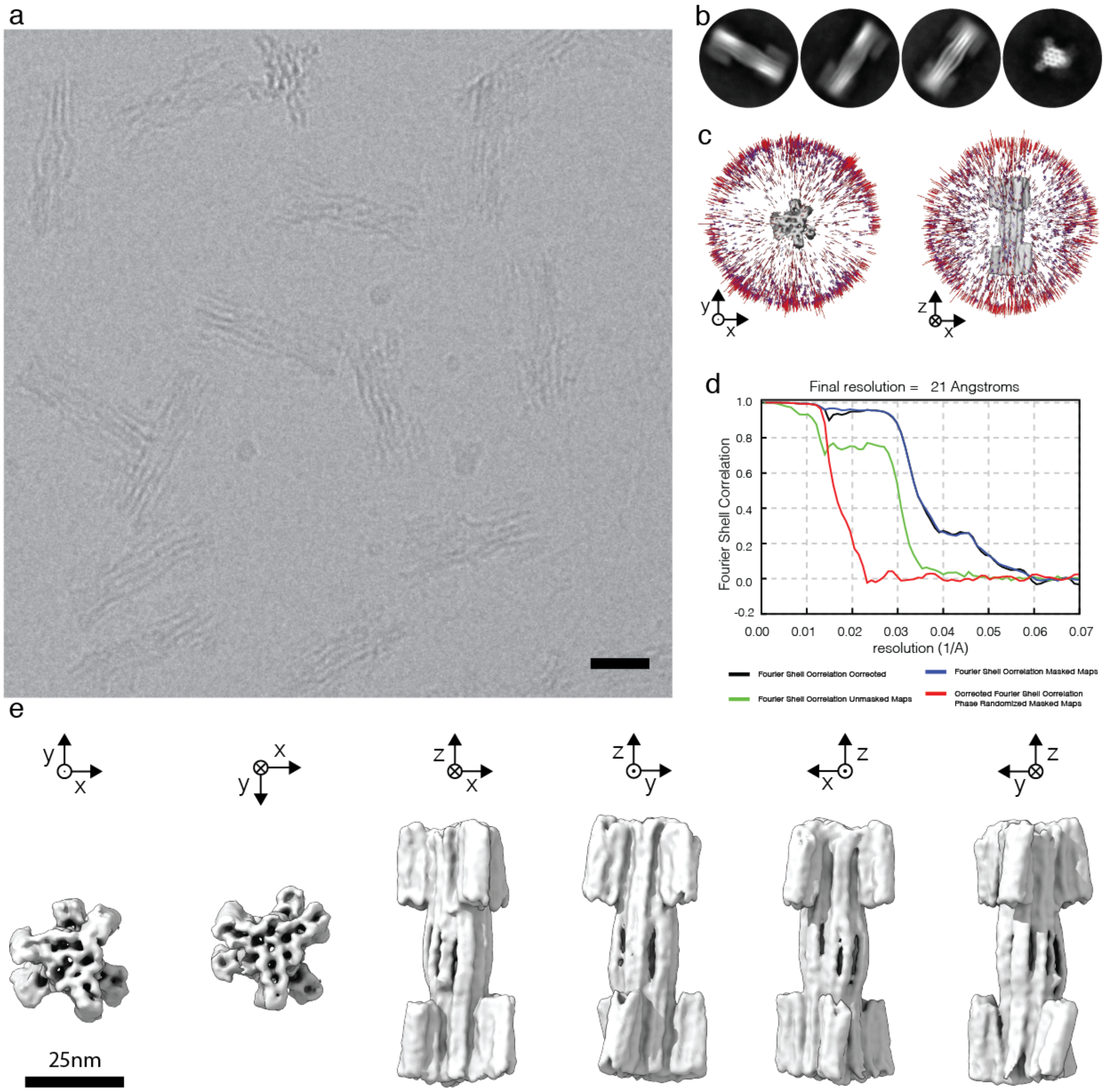

Supplementary Figure 14 | Cryo-EM map determination for dumbbell-like object v1. (a) Exemplary micrograph of a total of 220 movie stacks. Scale bar = 25 nm. (b) Representative 2D Class averages. (c) FSC plot. (d) Histogram representing the orientational distribution of particles. (e) Six different views of the electron density map.

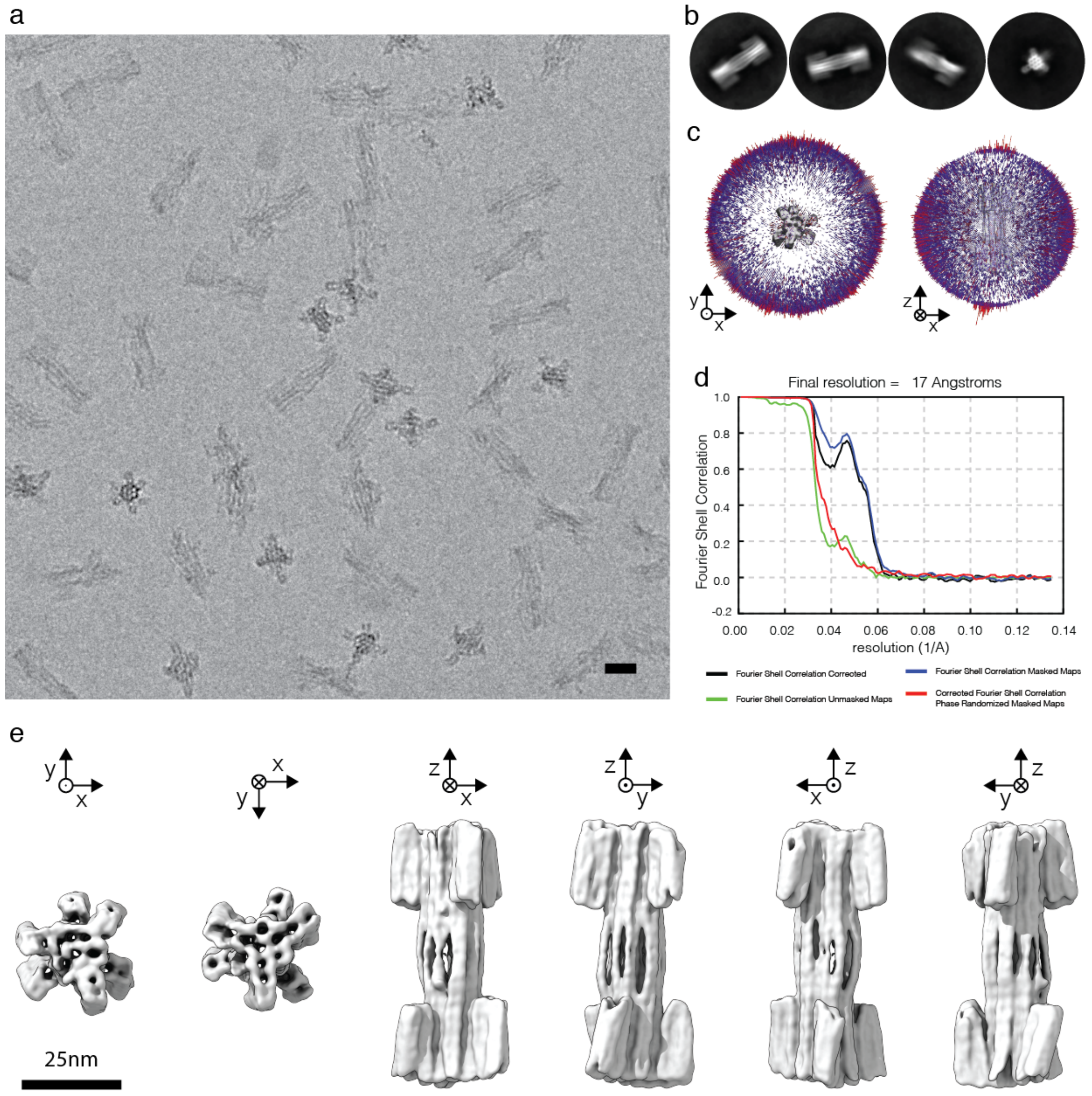

Supplementary Figure 15 | Cryo-EM map determination for dumbbell-like object v2. (a) Exemplary micrograph of a total of 736 movie stacks. Scale bar = 25 nm. (b) Representative 2D Class averages. (c) FSC plot. (d) Histogram representing the orientational distribution of particles. (e) Six different views of the electron density map.

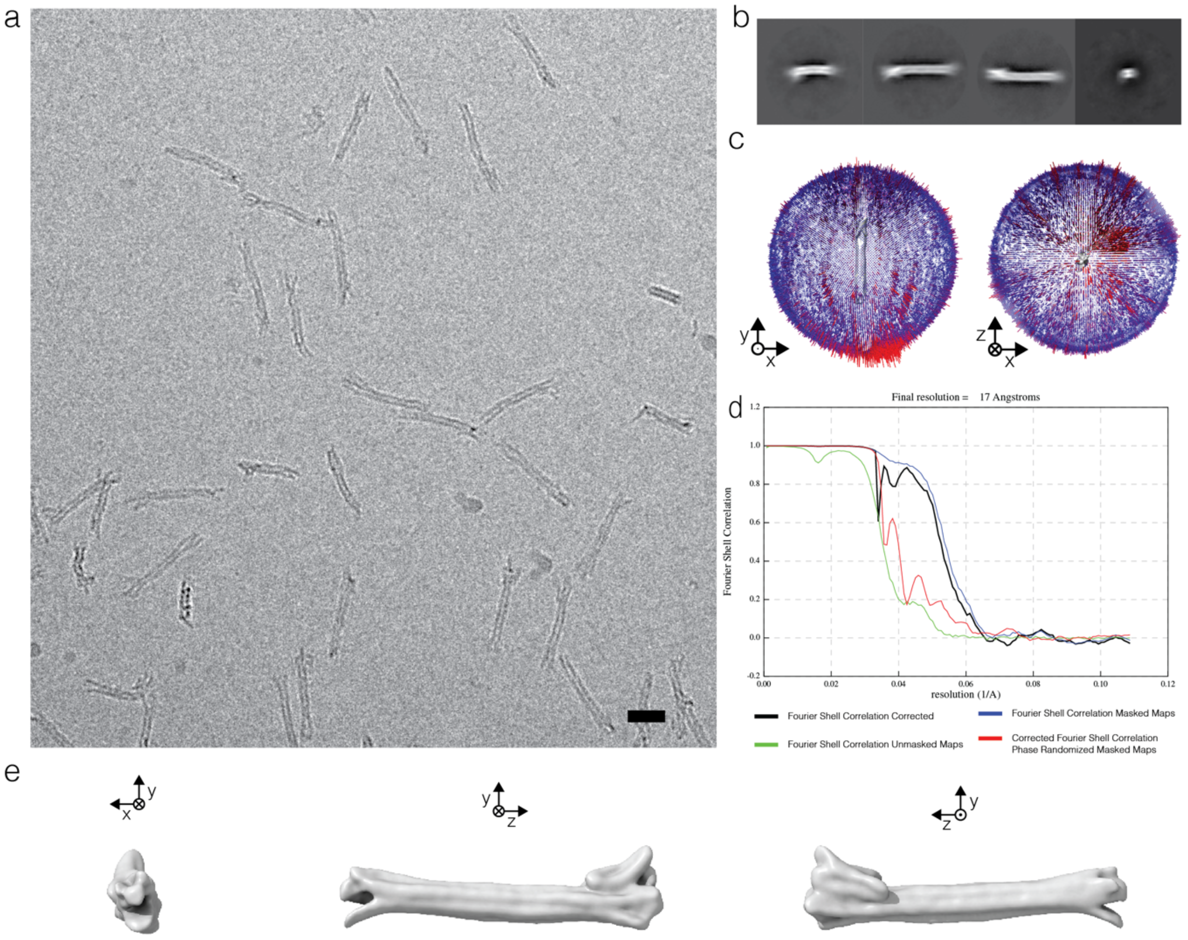

Supplementary Figure 16 | Cryo-EM map determination for the six-helix-tube v1. (a) Exemplary micrograph of a total of 744 movie stacks. Scale bar = 25 nm. (b) Representative 2D Class averages. (c) Histogram representing the orientational distribution of particles. (d) FSC plot. (e) Three different views of the electron density map.

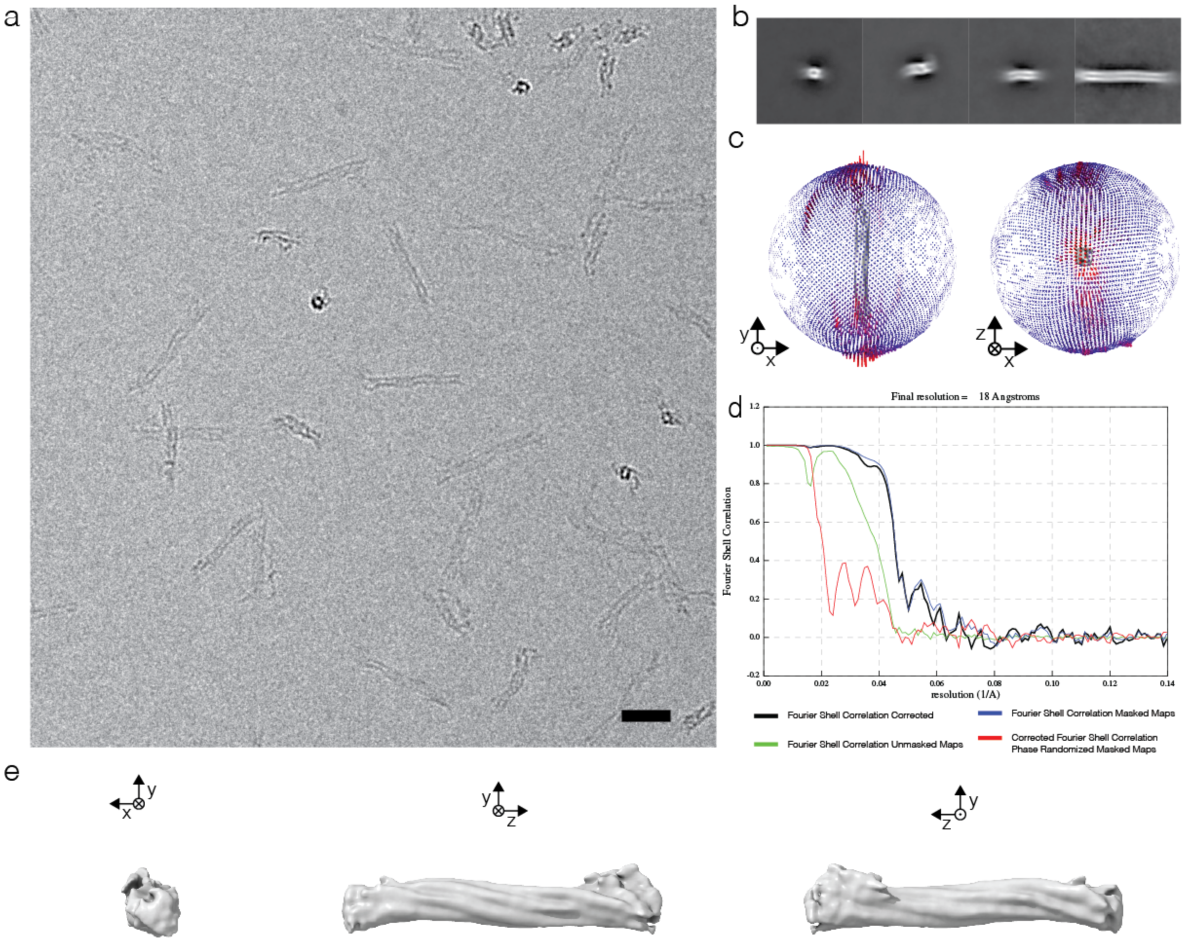

Supplementary Figure 17 | Cryo-EM map determination for the six-helix-tube v2. (a) Exemplary micrograph of a total of 989 movie stacks. Scale bar = 25 nm. (b) Representative 2D Class averages. (c) Histogram representing the orientational distribution of particles. (d) FSC plot. (e) Three different views of the electron density map.

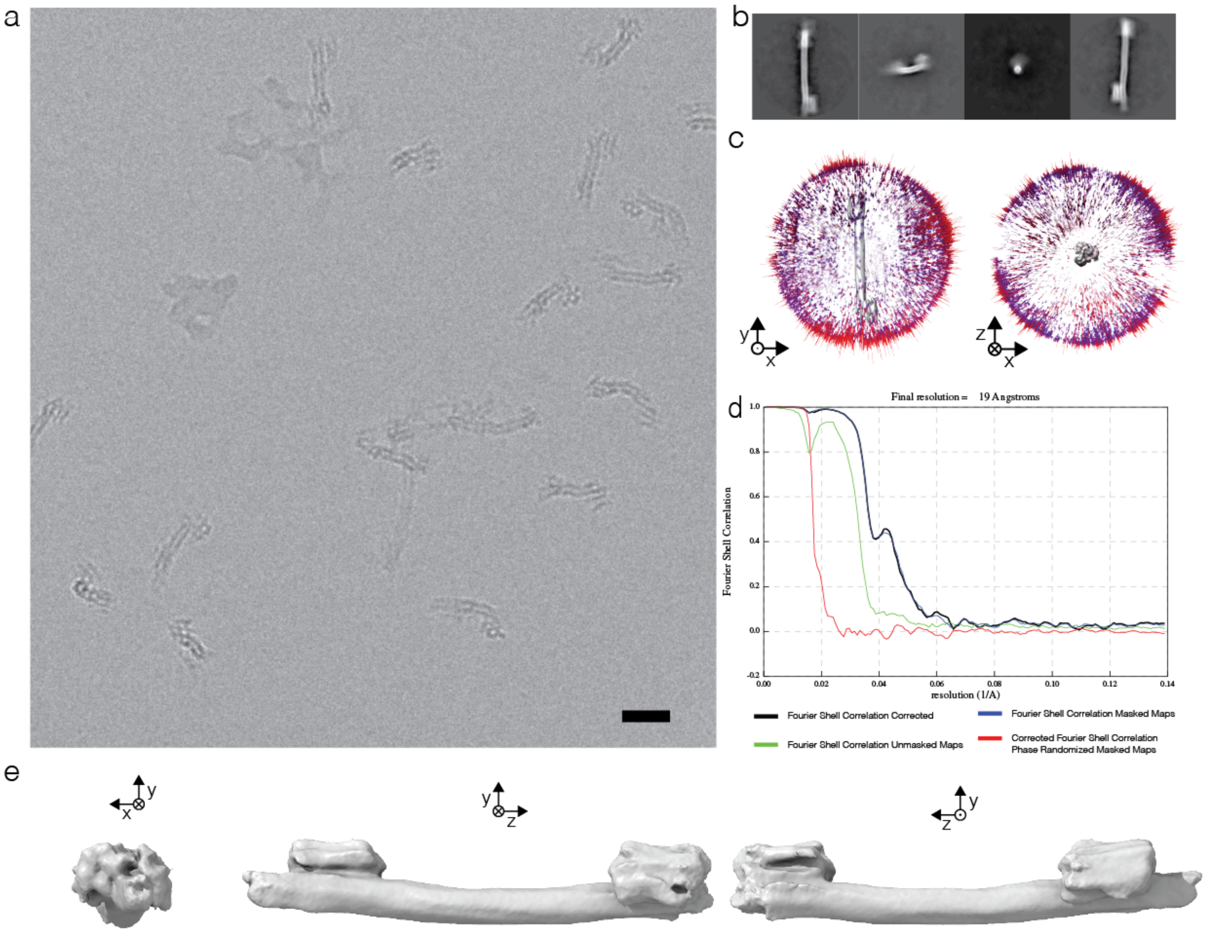

Supplementary Figure 18 | Cryo-EM map determination for the six-helix-tube v3. (a) Exemplary micrograph of a total of 358 movie stacks. Scale bar = 25 nm. (b) Representative 2D Class averages. (c) Histogram representing the orientational distribution of particles. (d) FSC plot. (e) Three different views of the electron density map.

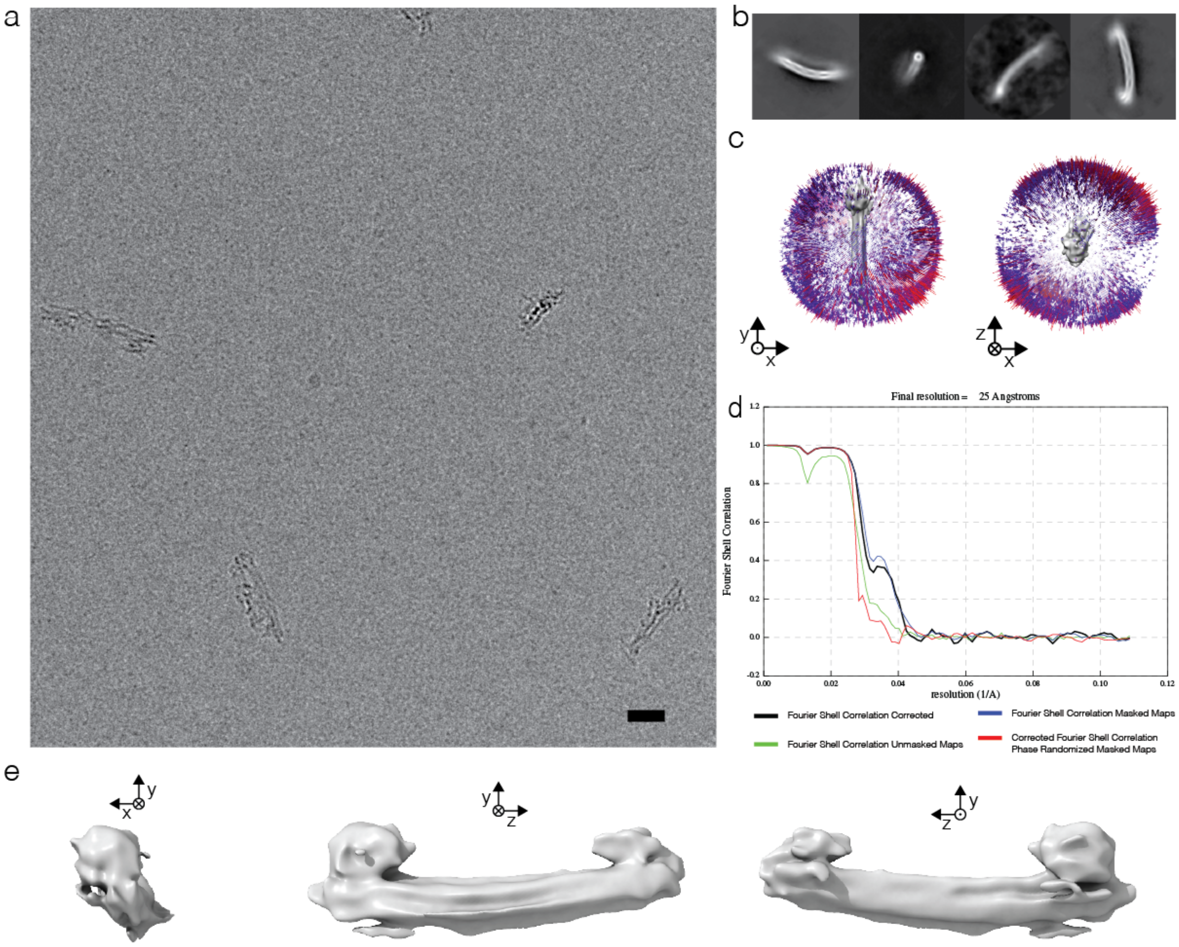

Supplementary Figure 19 | Cryo-EM map determination for the six-helix-tube v4. (a) Exemplary micrograph of a total of 2503 movie stacks. Scale bar = 25 nm. (b) Representative 2D Class averages. (c) Histogram representing the orientational distribution of particles. (d) FSC plot. (e) Three different views of the electron density map.

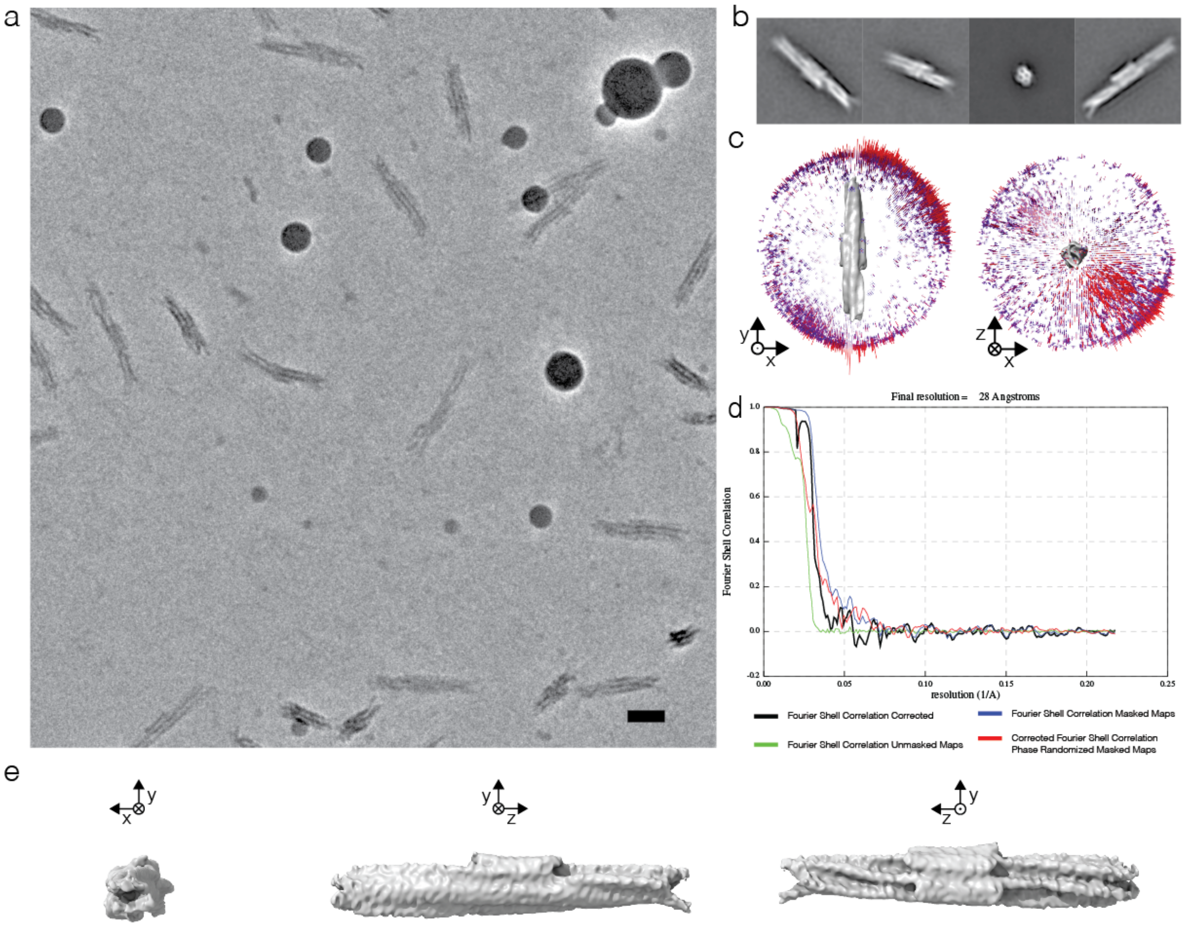

Supplementary Figure 20 | Cryo-EM map determination for the 10-helix tube. (a) Exemplary micrograph of a total of 421 movie stacks. Scale bar = 25 nm. (b) Representative 2D Class averages. (c) Histogram representing the orientational distribution of particles. (d) FSC plot. (e) Three different views of the electron density map.

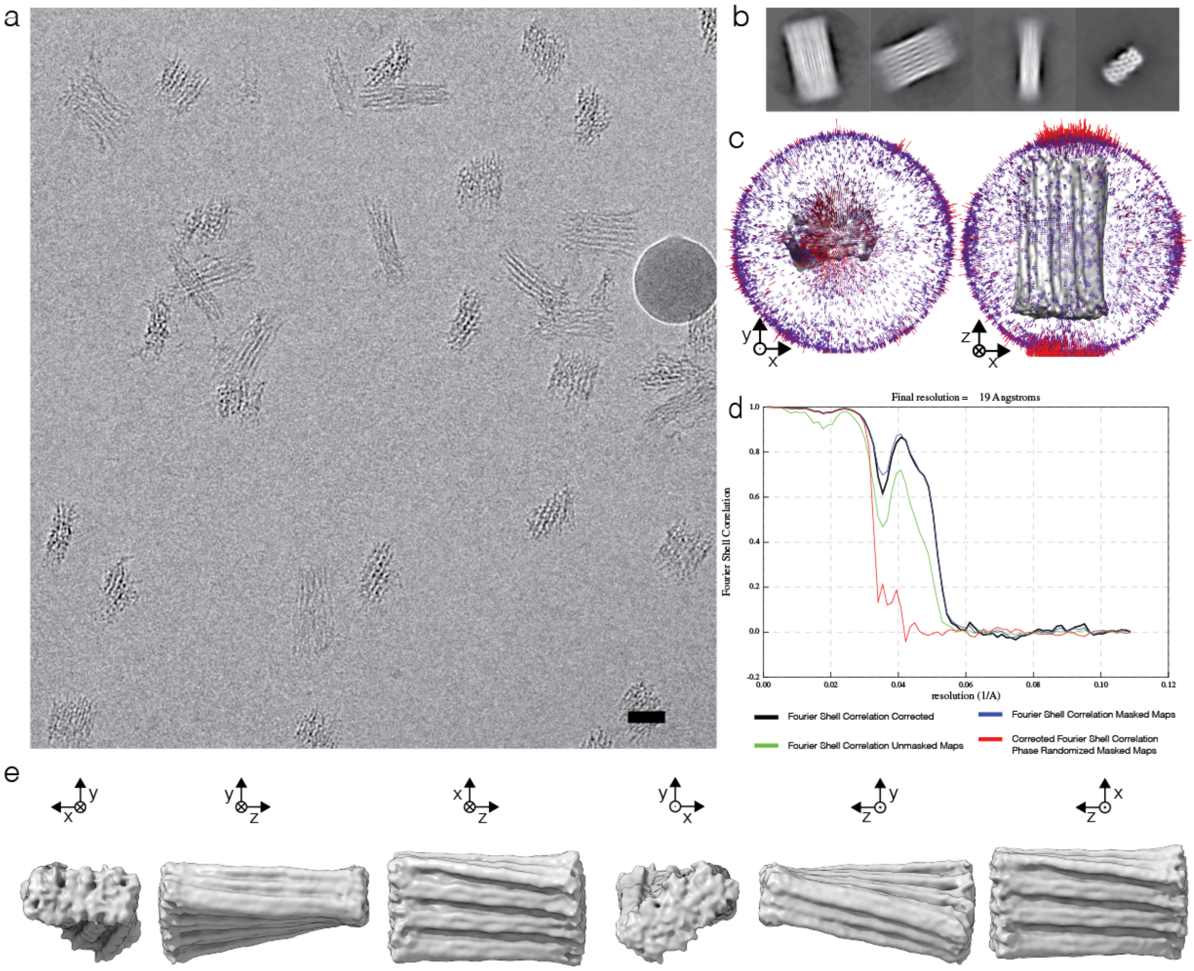

Supplementary Figure 21 | Cryo-EM map determination for 42 helix brick like DNA origami with reduced staple break density. (a) Exemplary micrograph of a total of 409 movie stacks. Scale bar = 25 nm. (b) Representative 2D Class averages. (c) Histogram representing the orientational distribution of particles. (d) FSC plot. (e) Six different views of the electron density map.

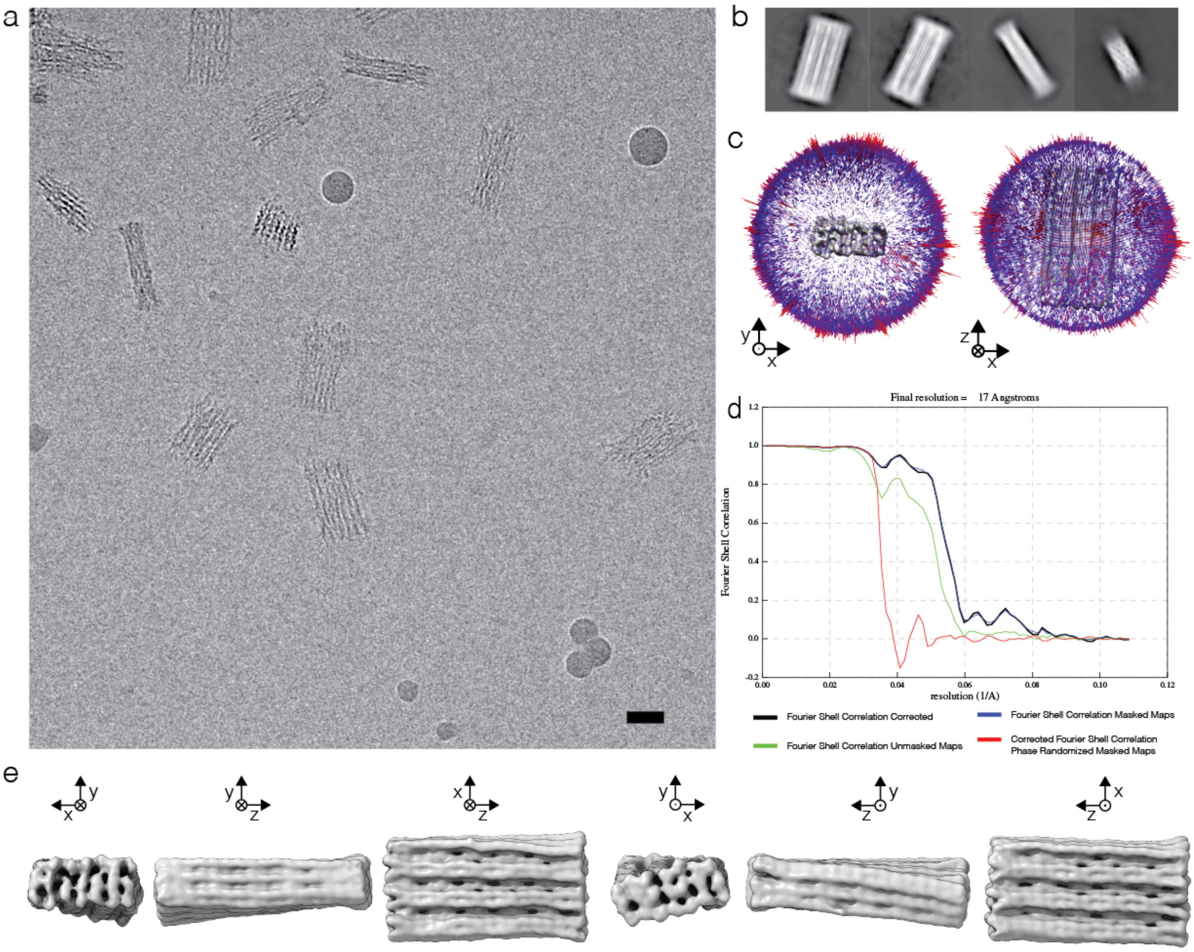

Supplementary Figure 22 | Cryo-EM map determination for 42 helix brick like DNA origami with reduced staple break density, UV point welded. (a) Exemplary micrograph of a total of 1228 movie stacks. Scale bar = 25 nm. (b) Representative 2D Class averages. (c) Histogram representing the orientational distribution of particles. (d) FSC plot. (e) Six different views of the electron density map.

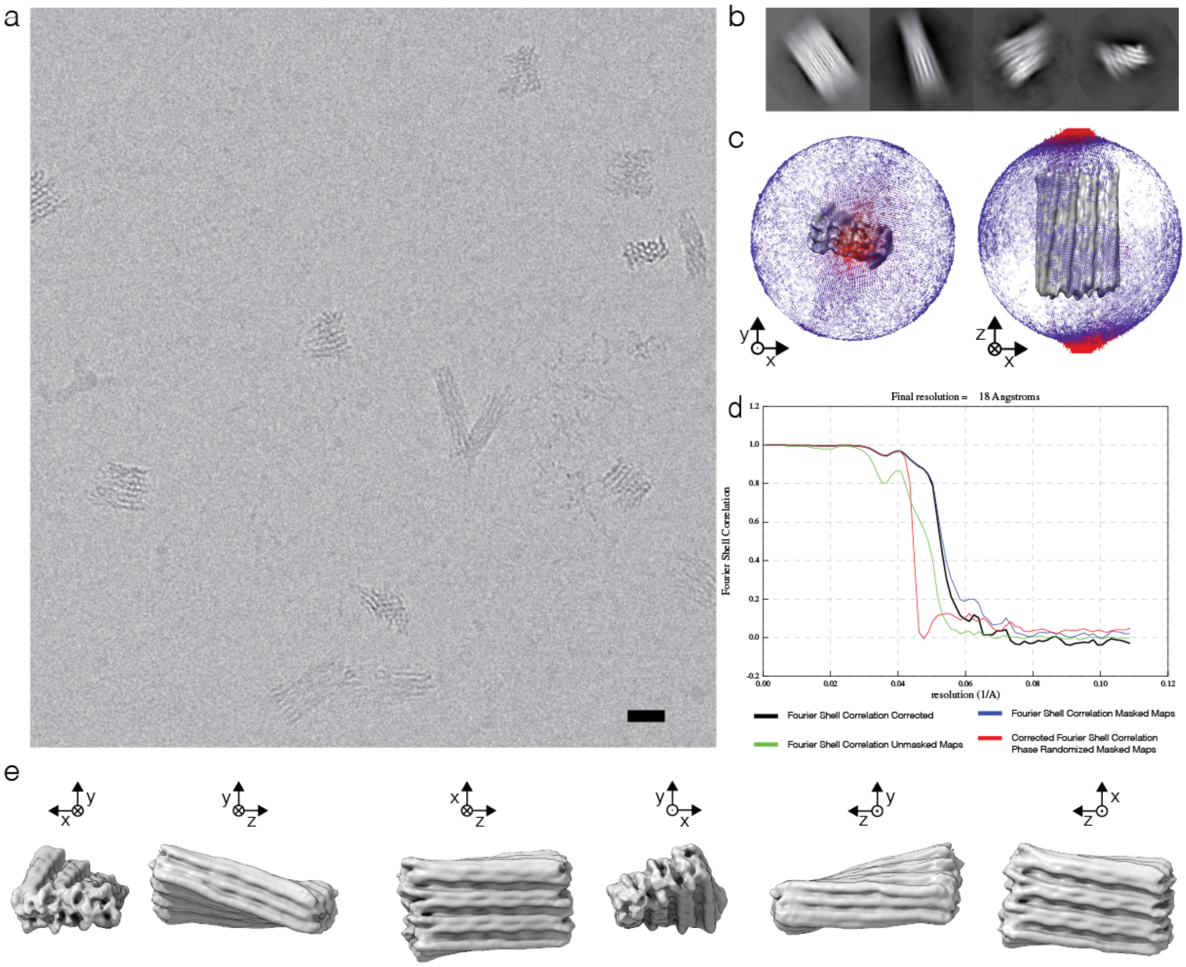

Supplementary Figure 23 | Cryo-EM map determination for 42 helix brick like DNA origami with aligned staple breaks. (a) Exemplary micrograph of a total of 1790 movie stacks. Scale bar = 25 nm. (b) Representative 2D Class averages. (c) Histogram representing the orientational distribution of particles. (d) FSC plot. (e) Six different views of the electron density map.

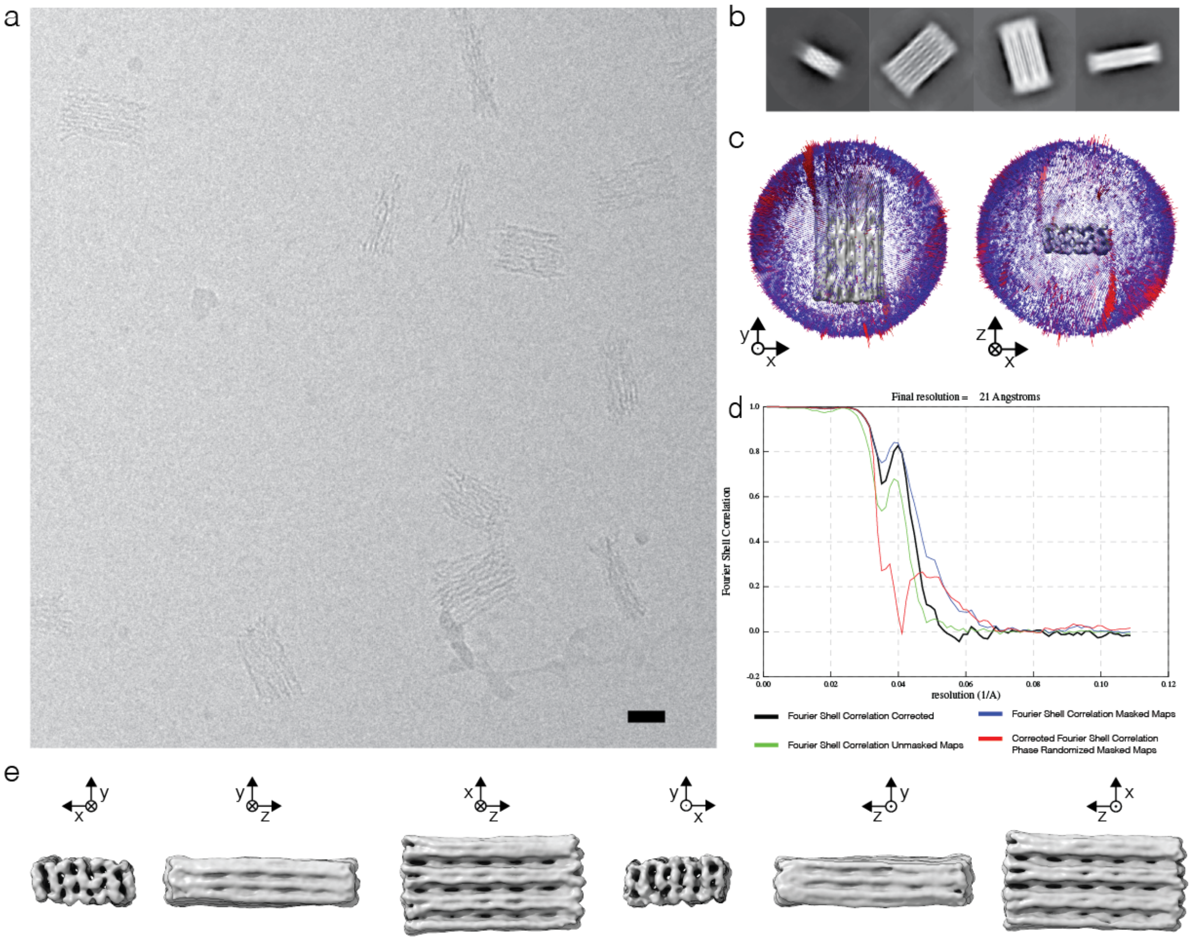

Supplementary Figure 24 | Cryo-EM map determination for 42 helix brick like DNA origami with aligned staple breaks, UV point welded. (a) Exemplary micrograph of a total of 1582 movie stacks. Scale bar = 25 nm. (b) Representative 2D Class averages. (c) Histogram representing the orientational distribution of particles. (d) FSC plot. (e) Six different views of the electron density map.

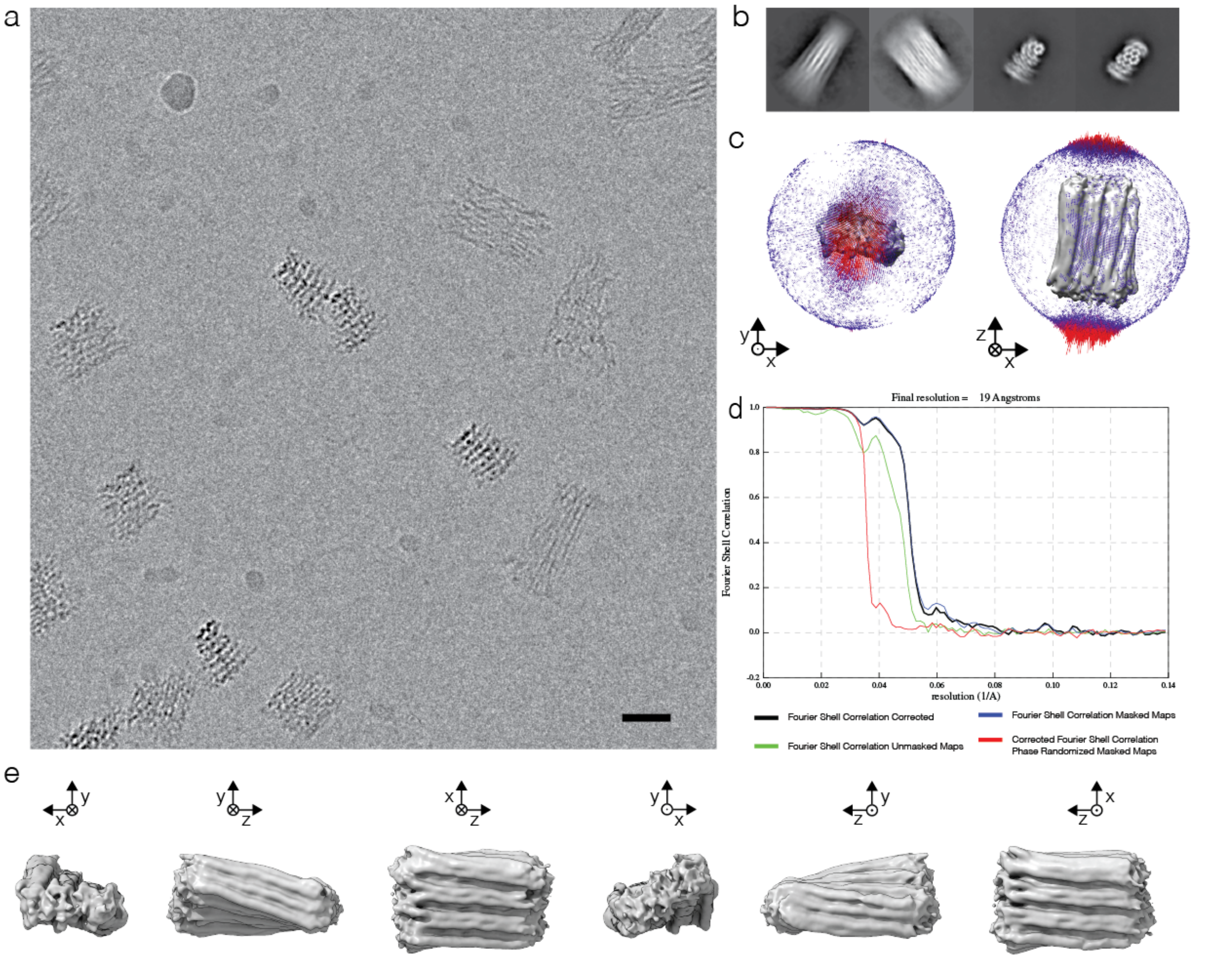

Supplementary Figure 25 | Cryo-EM map determination for 42 helix brick like DNA origami with aligned staple breaks and one T at each staple terminus. (a) Exemplary micrograph of a total of 1536 movie stacks. Scale bar = 25 nm. (b) Representative 2D Class averages. (c) Histogram representing the orientational distribution of particles. (d) FSC plot. (e) Six different views of the electron density map.

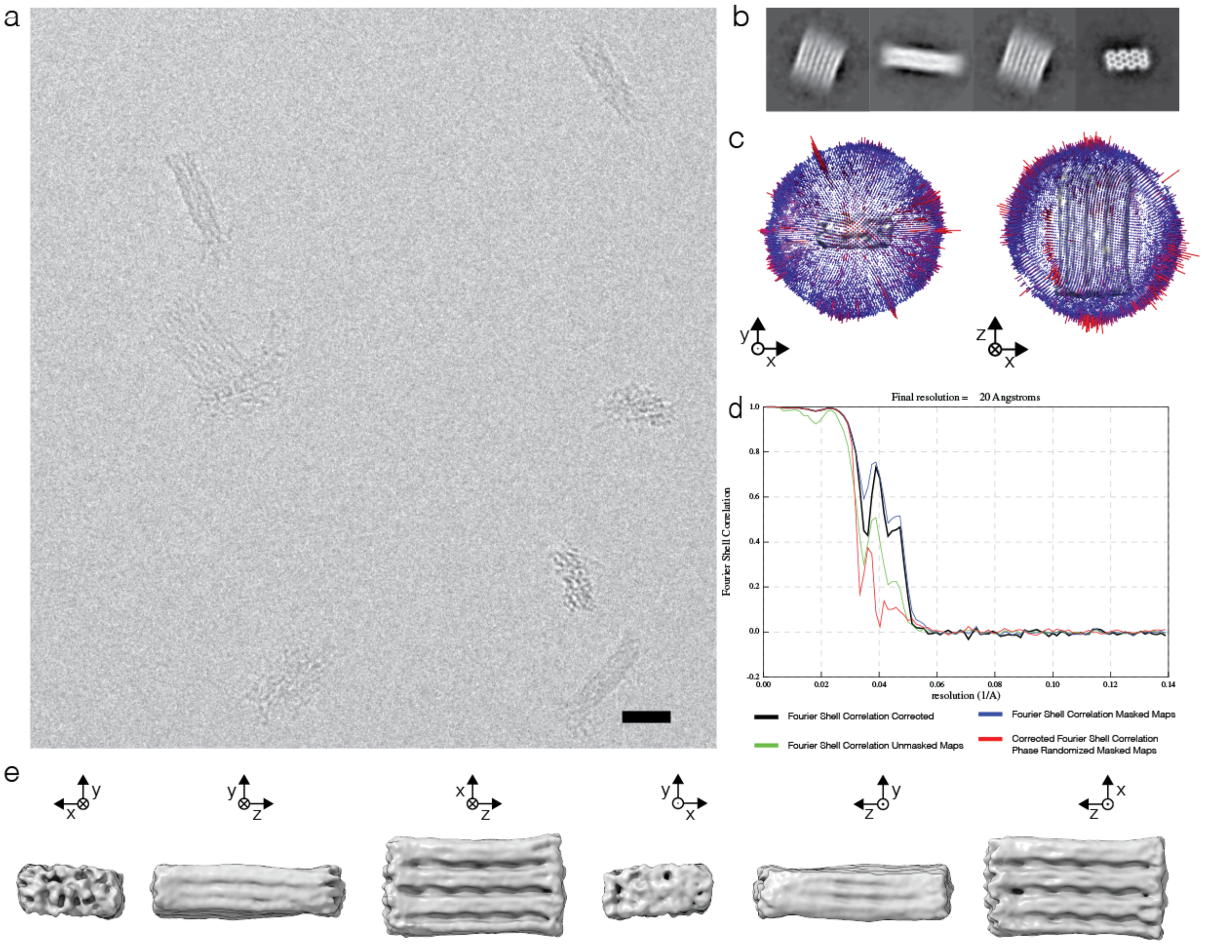

Supplementary Figure 26 | Cryo-EM map determination for 42 helix brick like DNA origami with aligned staple breaks and one T at each staple terminus, UV point welded. (a) Exemplary micrograph of a total of 2080 movie stacks. Scale bar = 25 nm. (b) Representative 2D Class averages. (c) Histogram representing the orientational distribution of particles. (d) FSC plot. (e) Six different views of the electron density map.

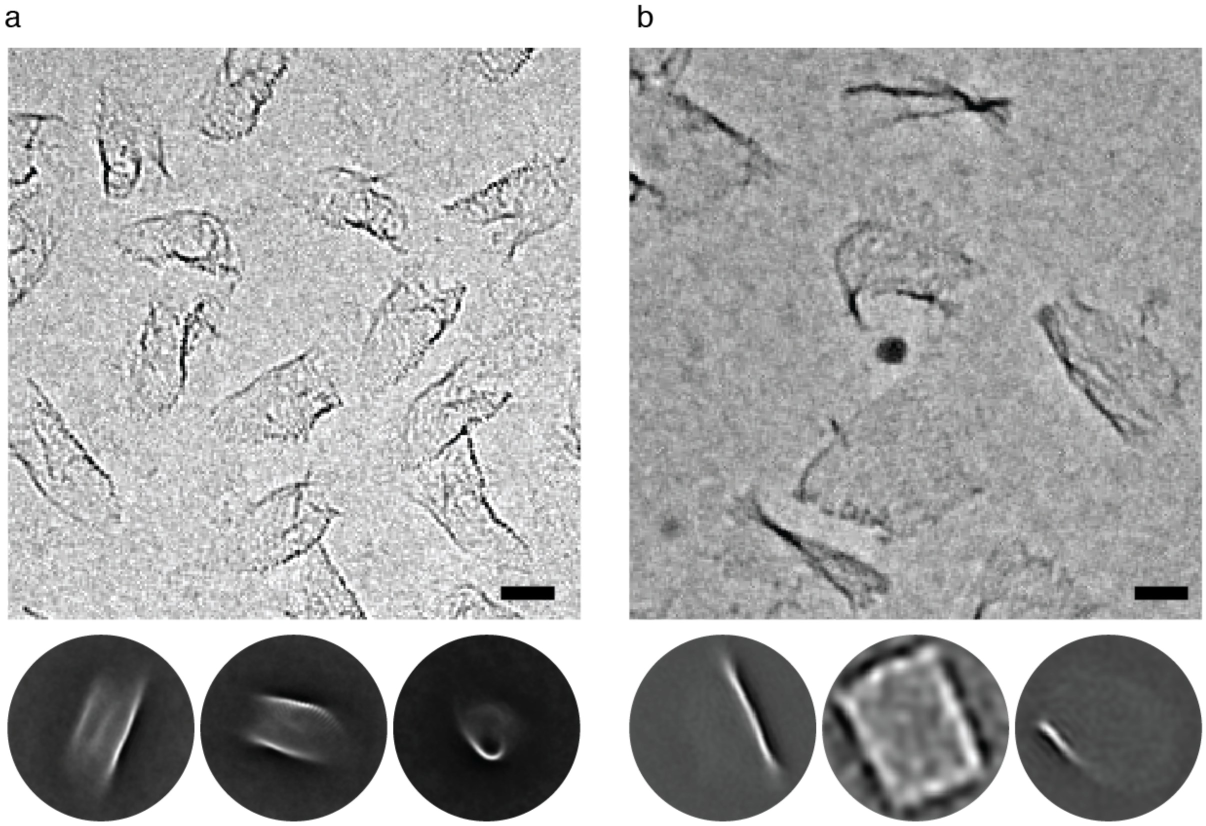

Supplementary Figure 27 | Cryo-EM imaging of single-layer DNA origami rectangle variants. (a) Exemplary micrograph of a total of 1167 movie stacks in free standing ice and 2D averages of a rectangle variant with default crossover spacing corresponding to a periodicity of 10.67bp/turn. (b) Exemplary micrograph of a total of 3365 movie stacks in free standing ice and 2D averages of a twist corrected rectangle variant with an average crossover spacing corresponding to a periodicity of 10.44bp/turn. Scale bars = 25 nm.

Supplementary Figure 28 | Six-helix-tube design variants. Left to right: section of the strand design diagram, Cryo-EM map with twist angle, laser scanned image of an agarose gel on which self-assembly reaction mixtures were electrophoresed. M = monomers, P = pocket. (a) Variant with nicks at crossovers for improved folding yield. (b) Variant with legacy strand breaking rules, gives byproducts. (c) Twist corrected variant (with deletions). (d) Twist enhanced variant (with insertions).

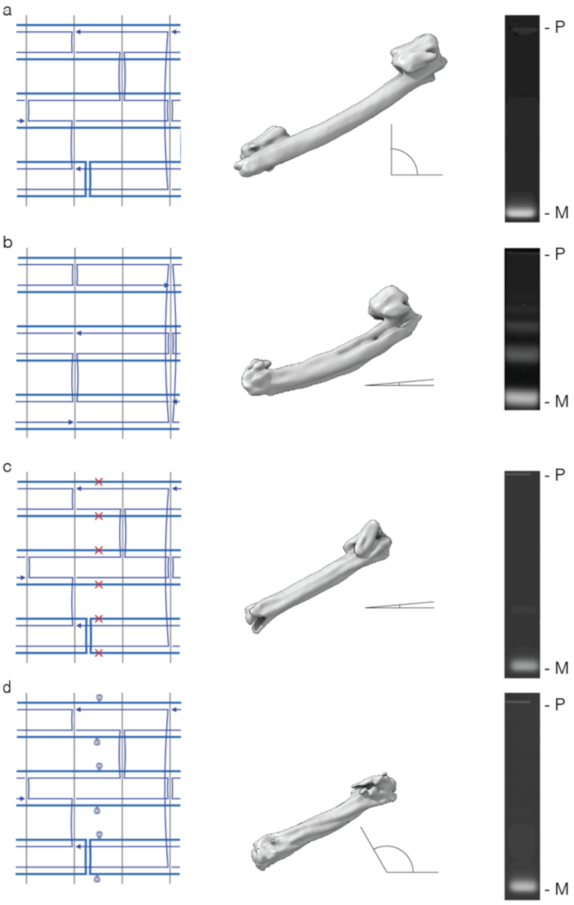

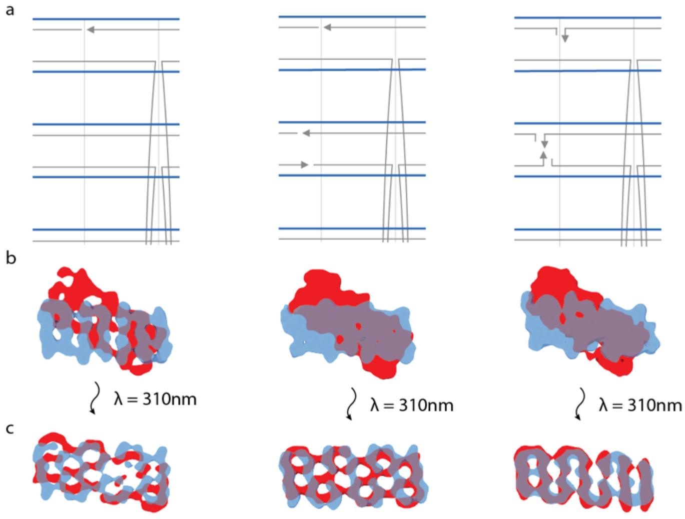

Supplementary Figure 29 | Strand break distribution and twist deformations. (a) Schematic representation of the distribution of staple breaks in three different 42 helix brick like DNA origami objects. Left: Reduced staple break density, misaligned ends. Middle: All staple ends were aligned along vertical planes. Right: At every staple terminus an additional unpaired thymidine was added. (b) Overlay of terminal cross-sectional slices at either end of the Cryo-EM maps determined for the variants as in a). (c) Overlay of terminal cross-sectional slices at either end of the Cryo-EM maps determined for the UV point welded variants after illumination with 310nm UV light for 2h.

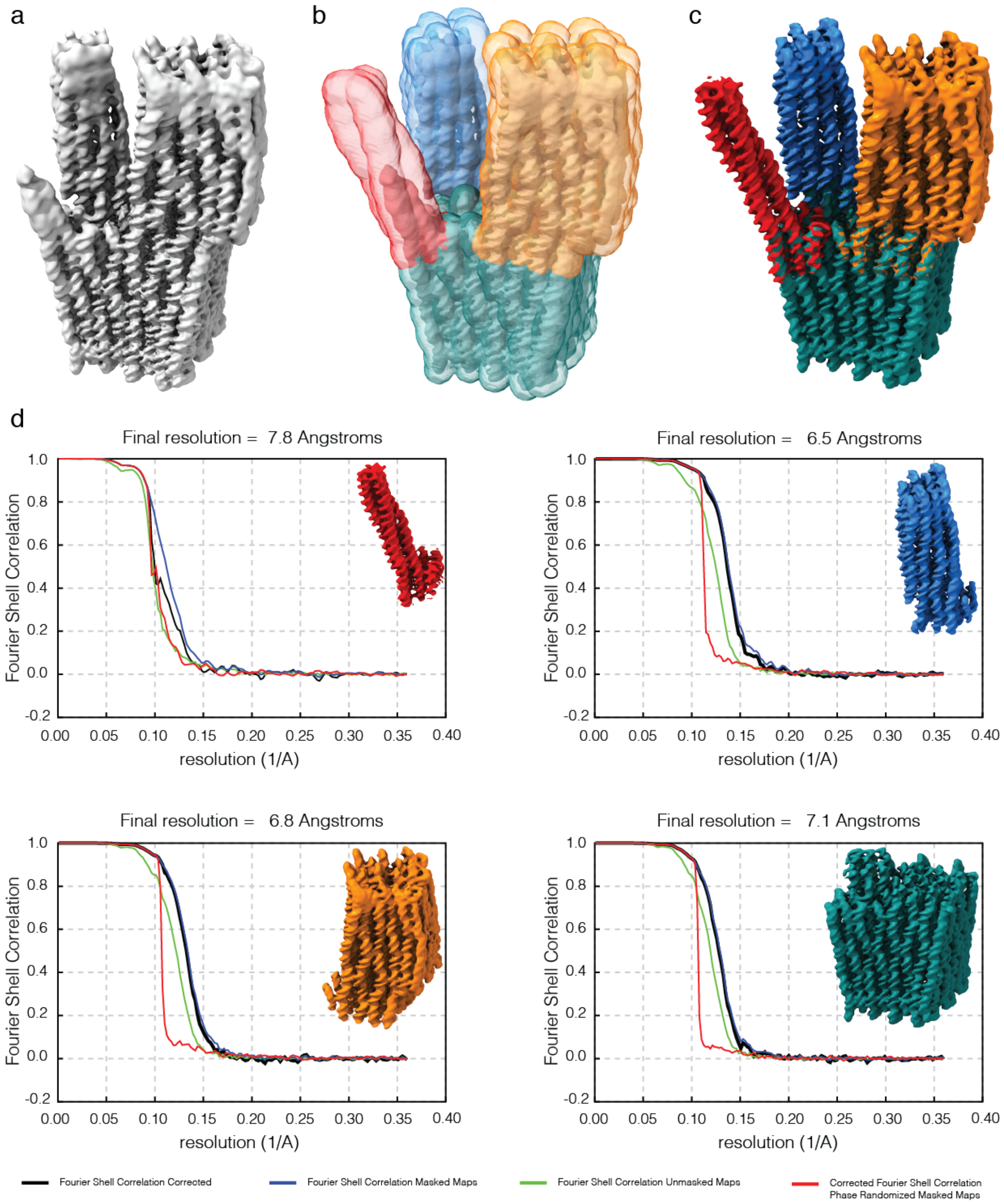

Supplementary Figure 30 | Multibody refinement of the Twisttower object. (a) Map from consensus refinement. (b) Masks used for multibody. (c) Individual bodies. (d) FSC curves for the individual bodies.

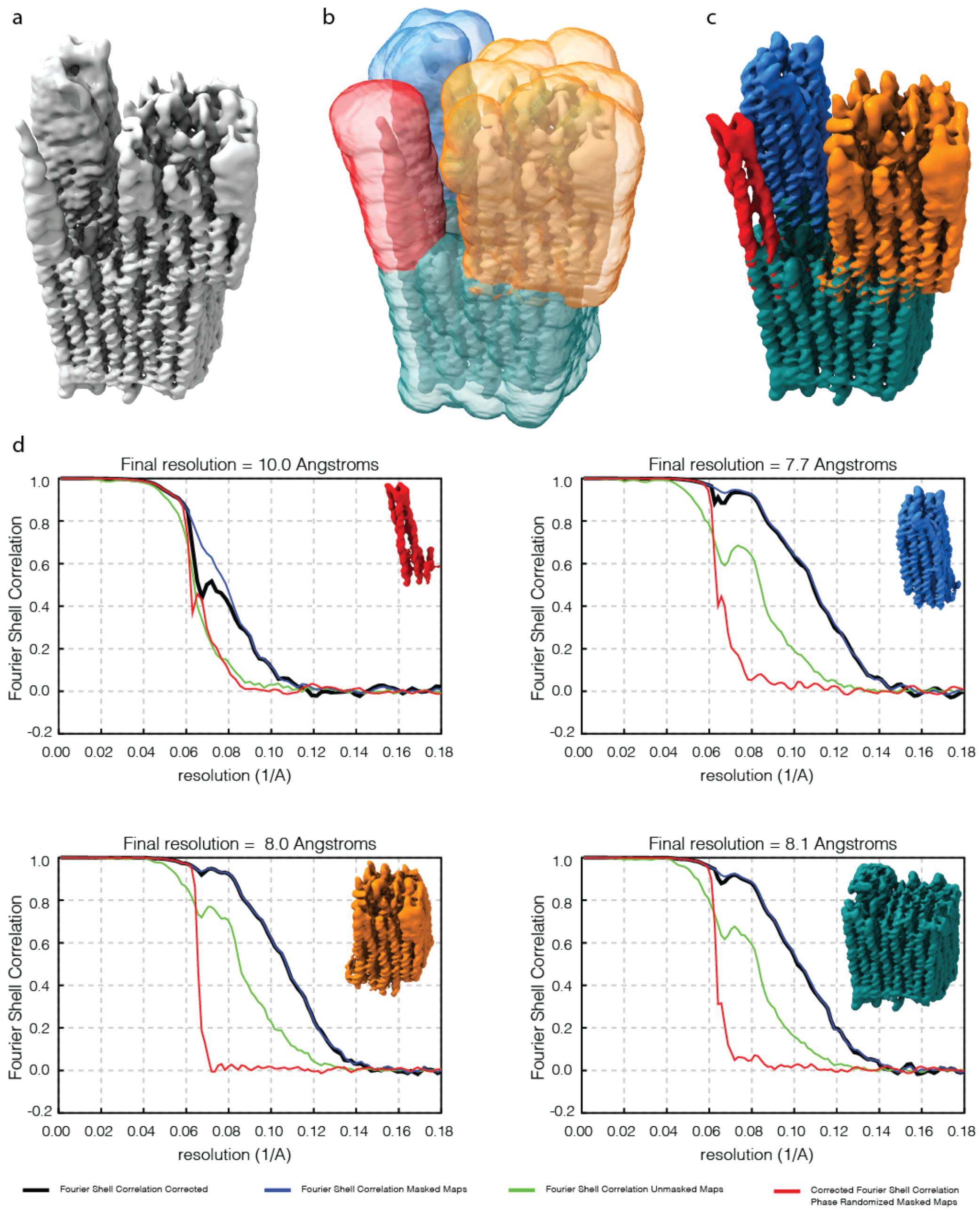

Supplementary Figure 31 | Multibody of twist-corrected Twisttower object. (a) Map from consensus refinement. (b) Masks used for multibody. (c) Individual bodies. (d) FSC curves for the individual bodies.

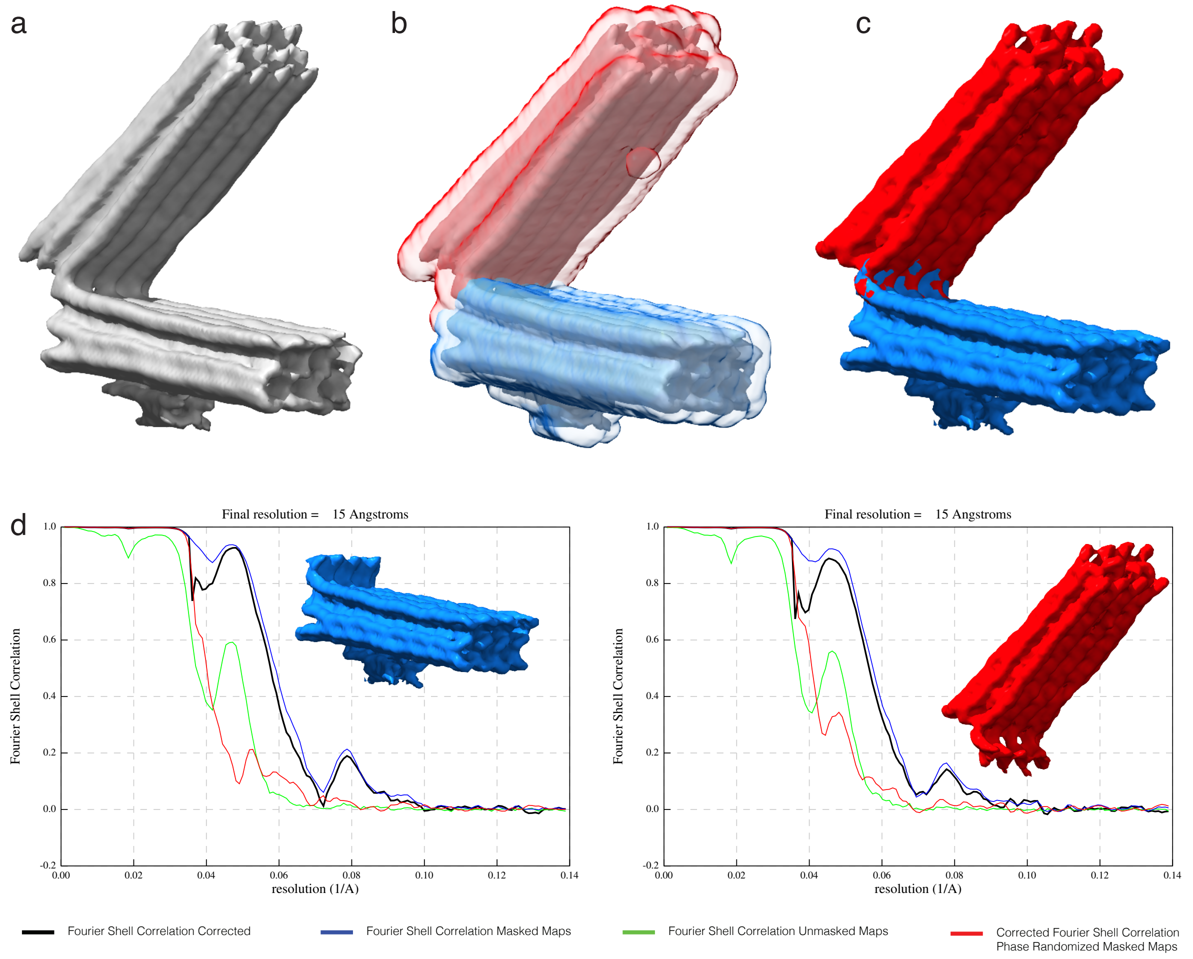

Supplementary Figure 32 | Multibody refinement of the hinged beam object v4. (a) Map from consensus refinement. (b) Masks used for multibody. (c) Individual bodies. (d) FSC curves for the individual bodies.

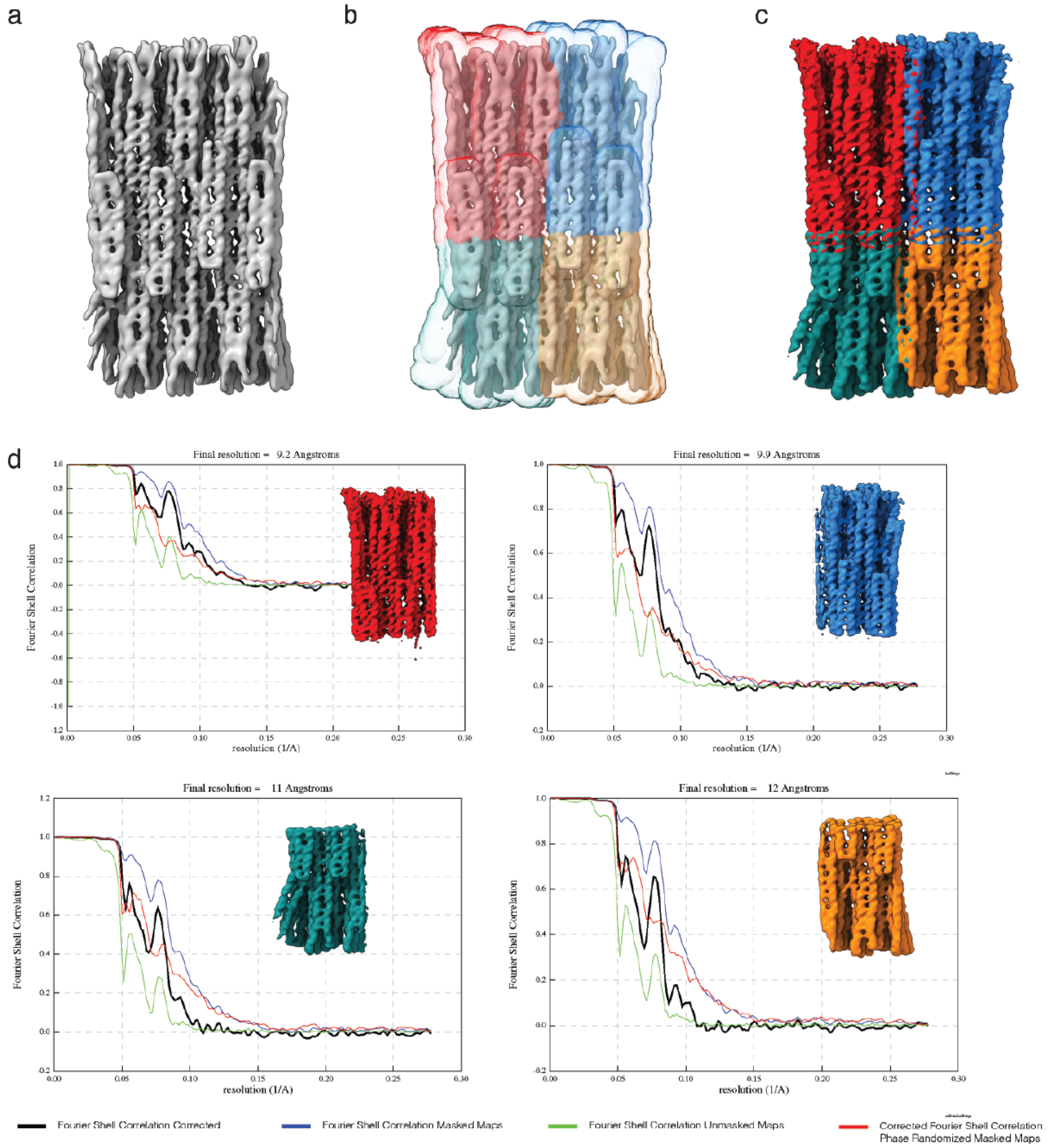

Supplementary Figure 33 | Multibody refinement of 48-helix bundle Brick. (a) Map from consensus refinement. (b) Masks used for multibody. (c) Individual bodies. (d) FSC curves for the individual bodies.

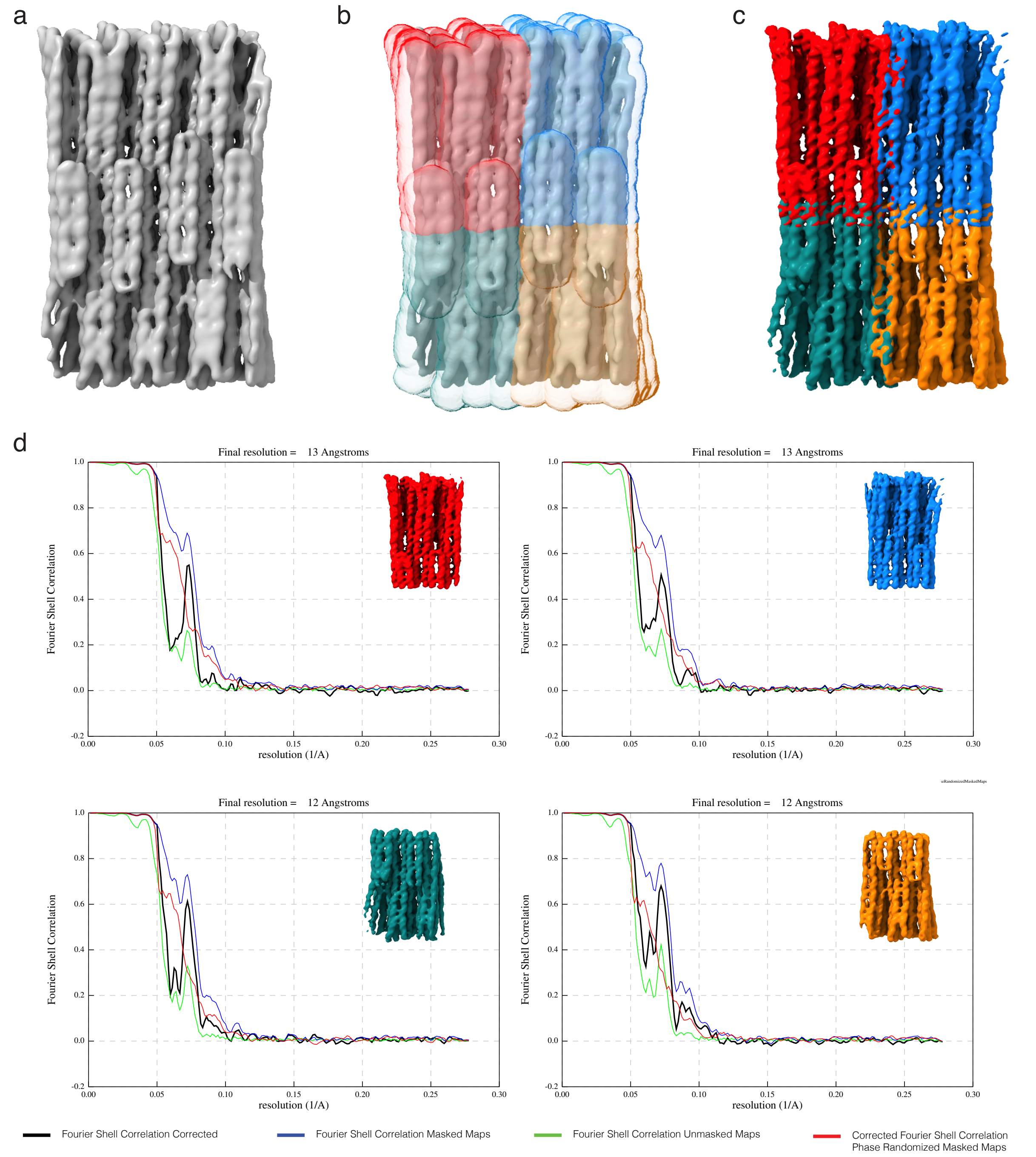

Supplementary Figure 34 | Multibody refinement of 48-helix bundle Brick with one T at all staple crossovers. (a) Map from consensus refinement. (b) Masks used for multibody. (c) Individual bodies. (d) FSC curves for the individual bodies.

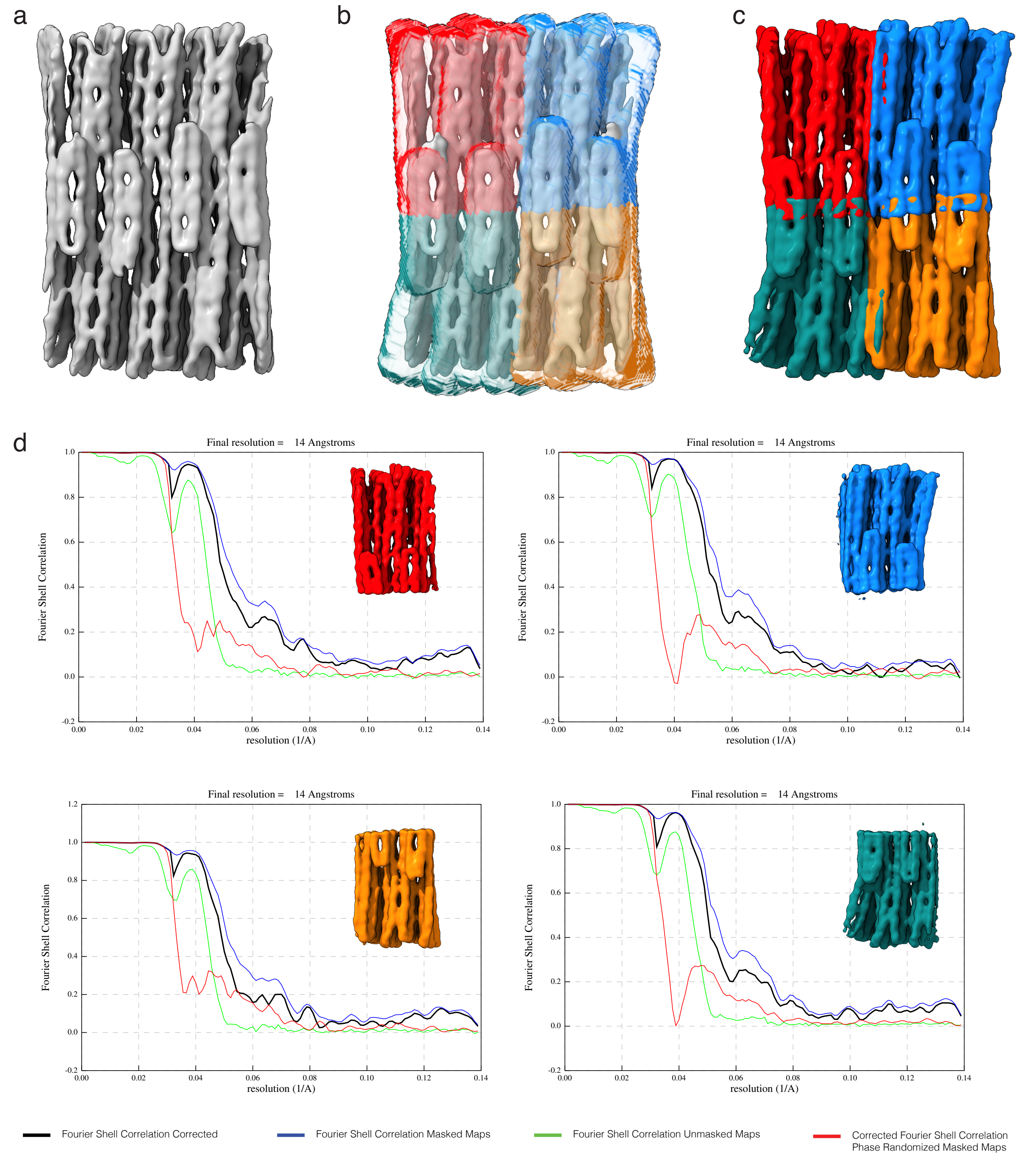

Supplementary Figure 35 | Multibody refinement of 48-helix bundle Brick with two Ts at all staple crossovers. (a) Map from consensus refinement. (b) Masks used for multibody. (c) Individual bodies. (d) FSC curves for the individual bodies.

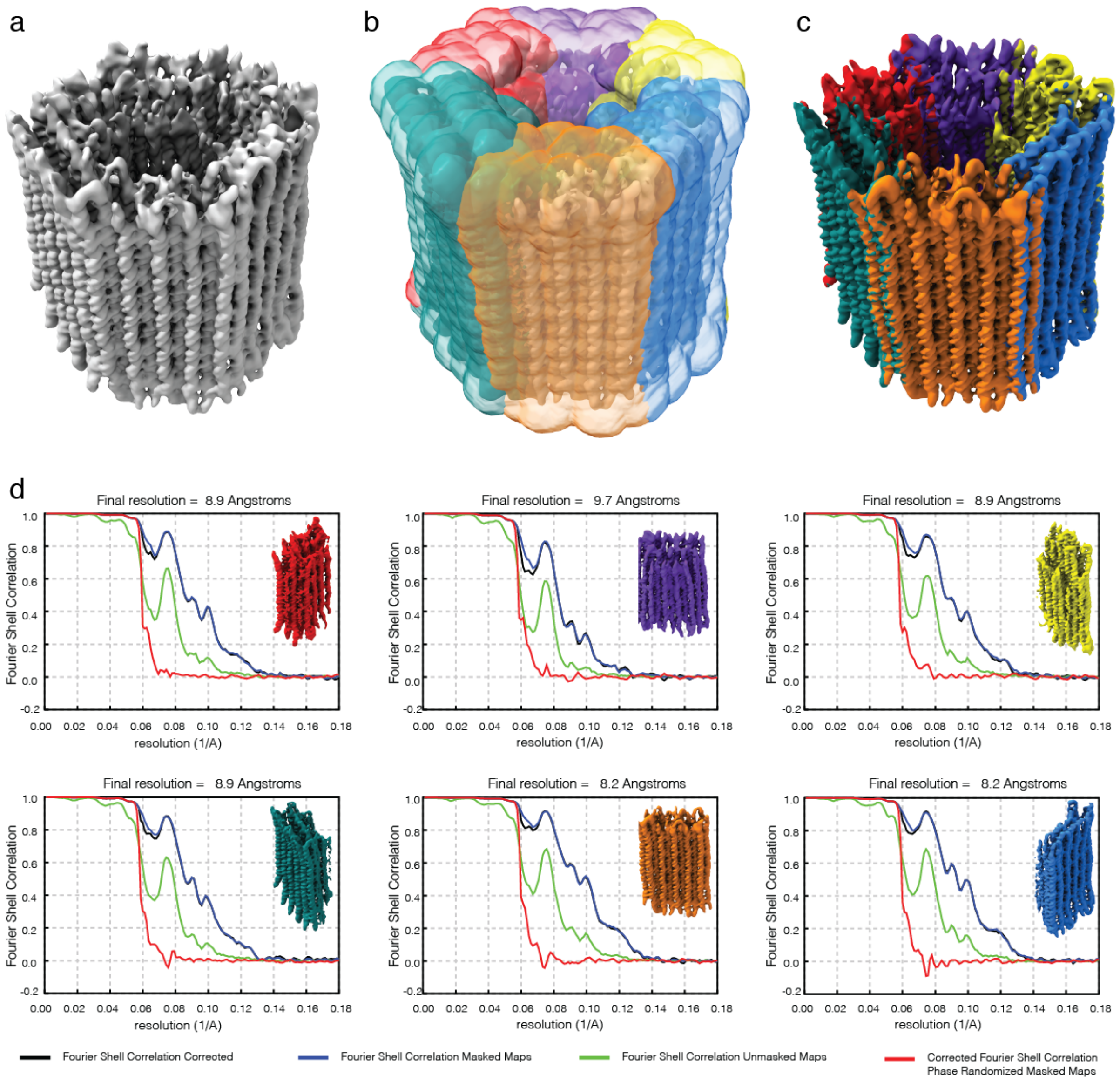

Supplementary Figure 36 | Multibody refinement of 126-helix bundle. (a) Map from consensus refinement. (b) Masks used for multibody. (c) Individual bodies. (d) FSC curves for the individual bodies.

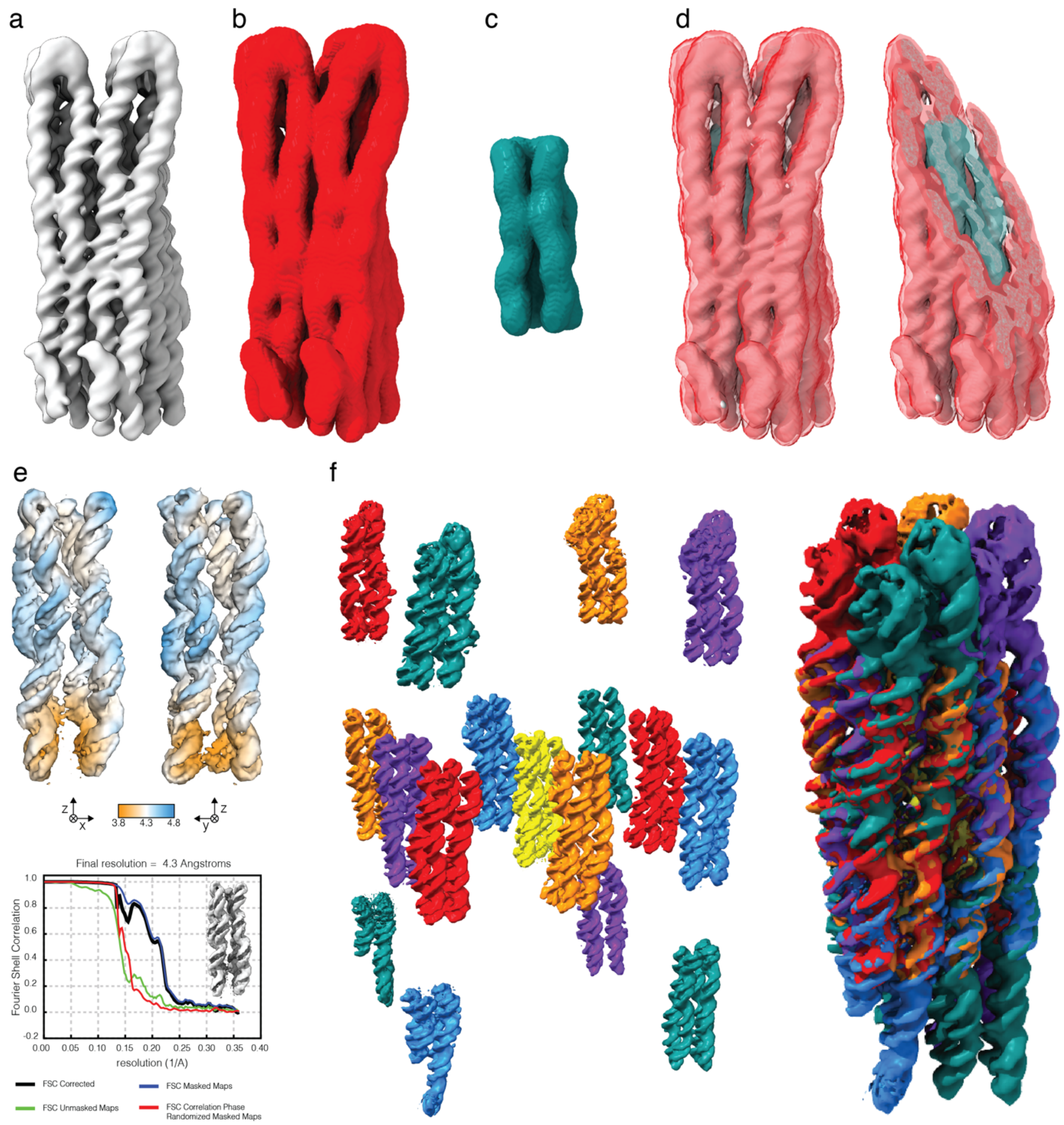

Supplementary Figure 37: Focused scanning refinement. (a) Map from consensus refinement (here: 4x4 domain in Twisttower object). **(b-d)** Masks used for refinement. Green = focal region of interest. Red = context. **(e)** Refined map of the focal region of interest with FSC curves. Colorbar indicates local resolution. **(f)** Scanning refinements of the 4x4 domain. Left = exploded view.

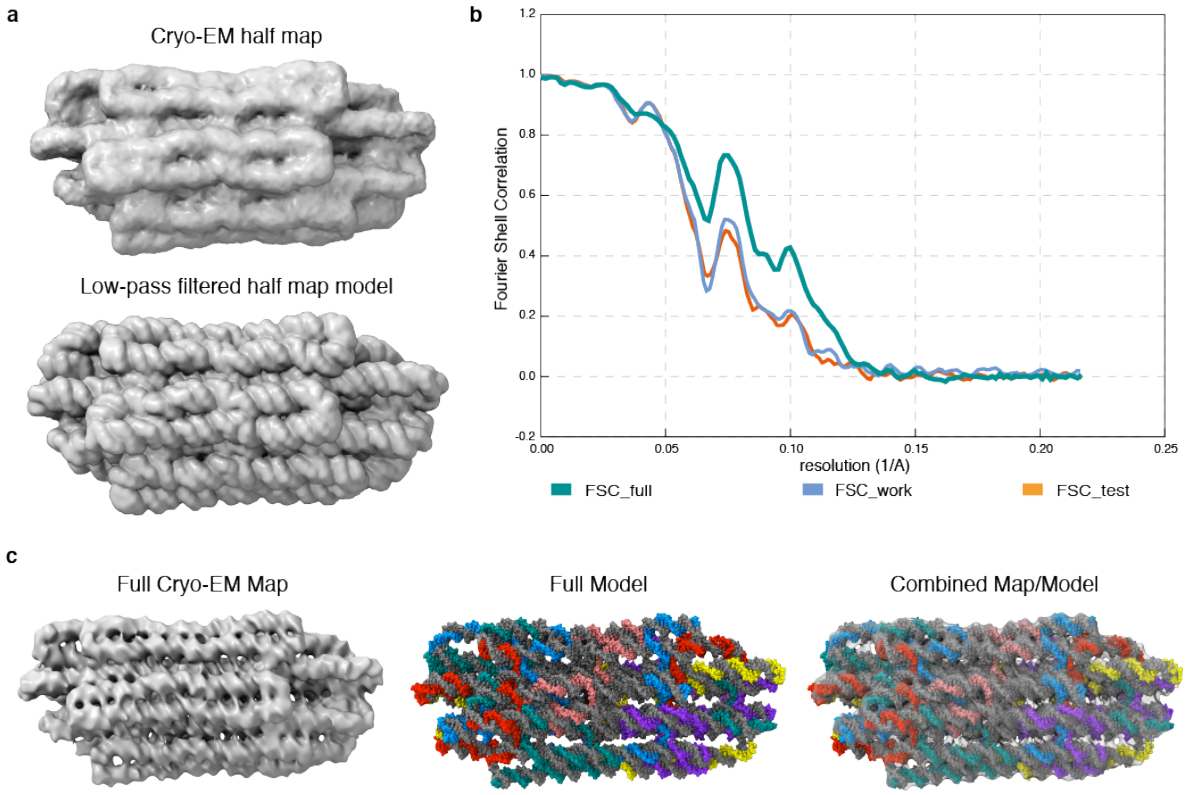

Supplementary Figure 38 | Atomic model validation using Fourier Shell Correlation (FSC). (a) First, the fitting procedure is performed using one of the two experimental half-maps. For each fitted model, a low pass-filtered map is created at the resolution of the experimental map. **(b)** Comparison of the FSC of the full cryo-EM map and the final fitted model (FSC_full), model for half map 1 with half map 1 (FSC_work) and model for half map 1 with half map 2 (FSC_test). Strong deviations of FSC_work and FSC_test would indicate overfitting of the dataset. **(c)** If the two FSCs for the fitted models do not differ significantly, the procedure can be applied to the full dataset creating a fitted atomic model of the high resolution cryo electron density map.

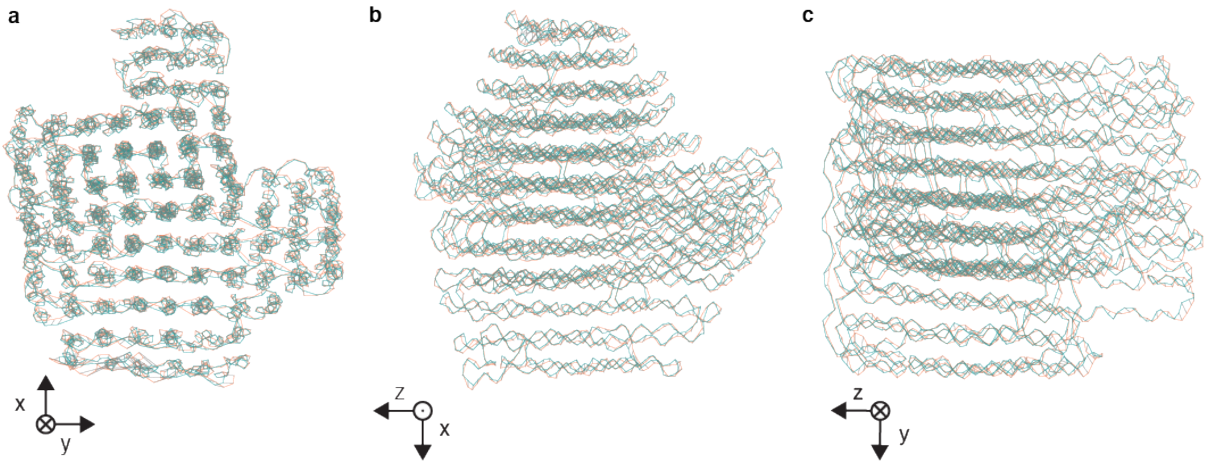

Supplementary Figure 39 | Atomic model comparison. (a-c) Lines give helical axes of the atomic models of the Pointer v1 (Bai et, ref 15) (green) and our cascaded fitting procedure (orange). Connecting lines (grey) depict the displacement at every 2nd base position. The cross-correlation of the new model with the Cryo-EM map is 0.913 (slightly improved over the previously published model (0.895)). The overall RMSD of old and new model is with 6.025 Å well below map resolution.

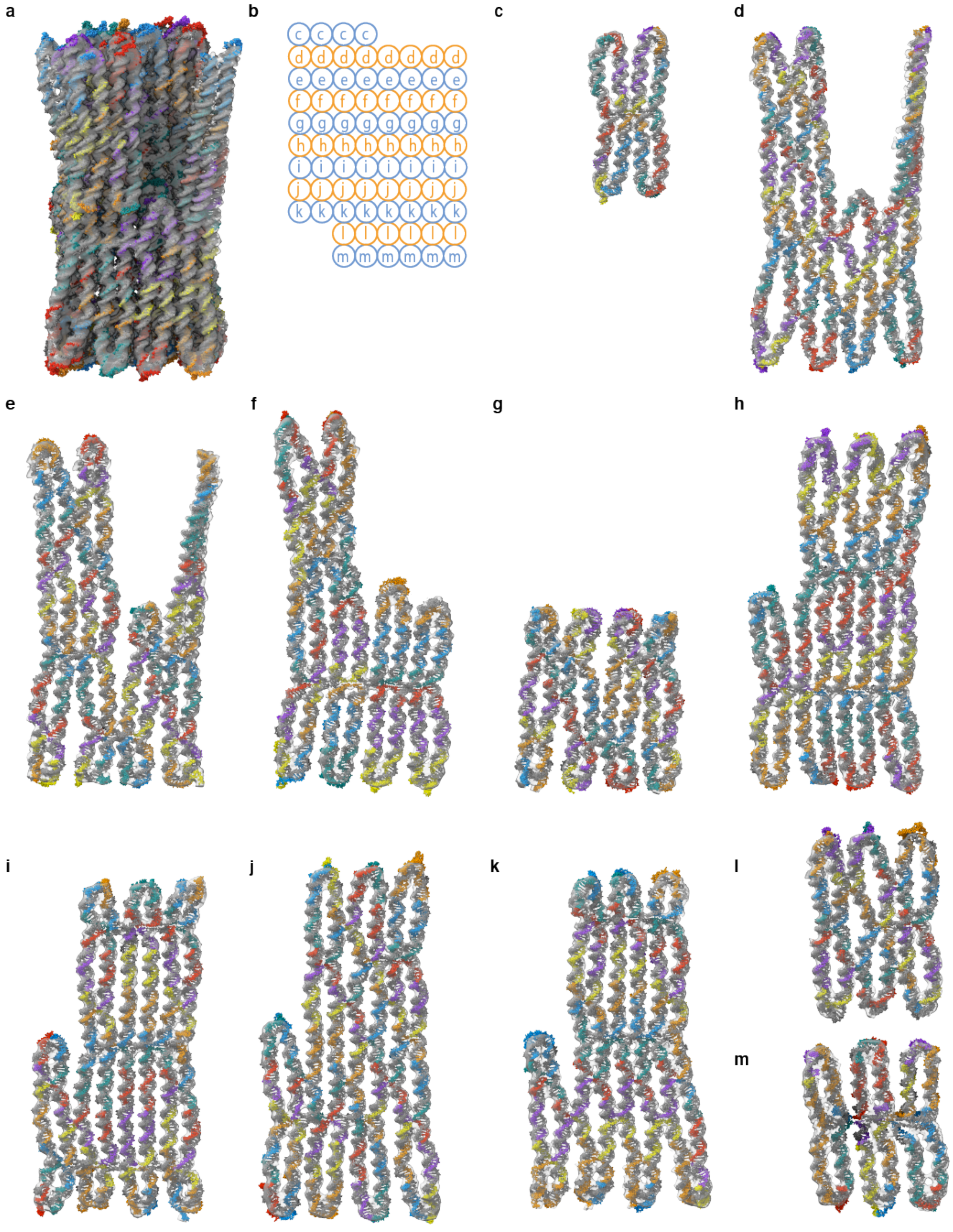

Supplementary Figure 40 | Slice-by-slice comparison of atomic model versus Cryo-EM map for the Twisttower. (a) Full 3D overlay of Cryo-EM map and atomic model. **(b)** Cross sectional scheme with slice indices. **(c-m)** Slices of the Cryo-EM map and atomic model. Panel labels as in **(b)**.

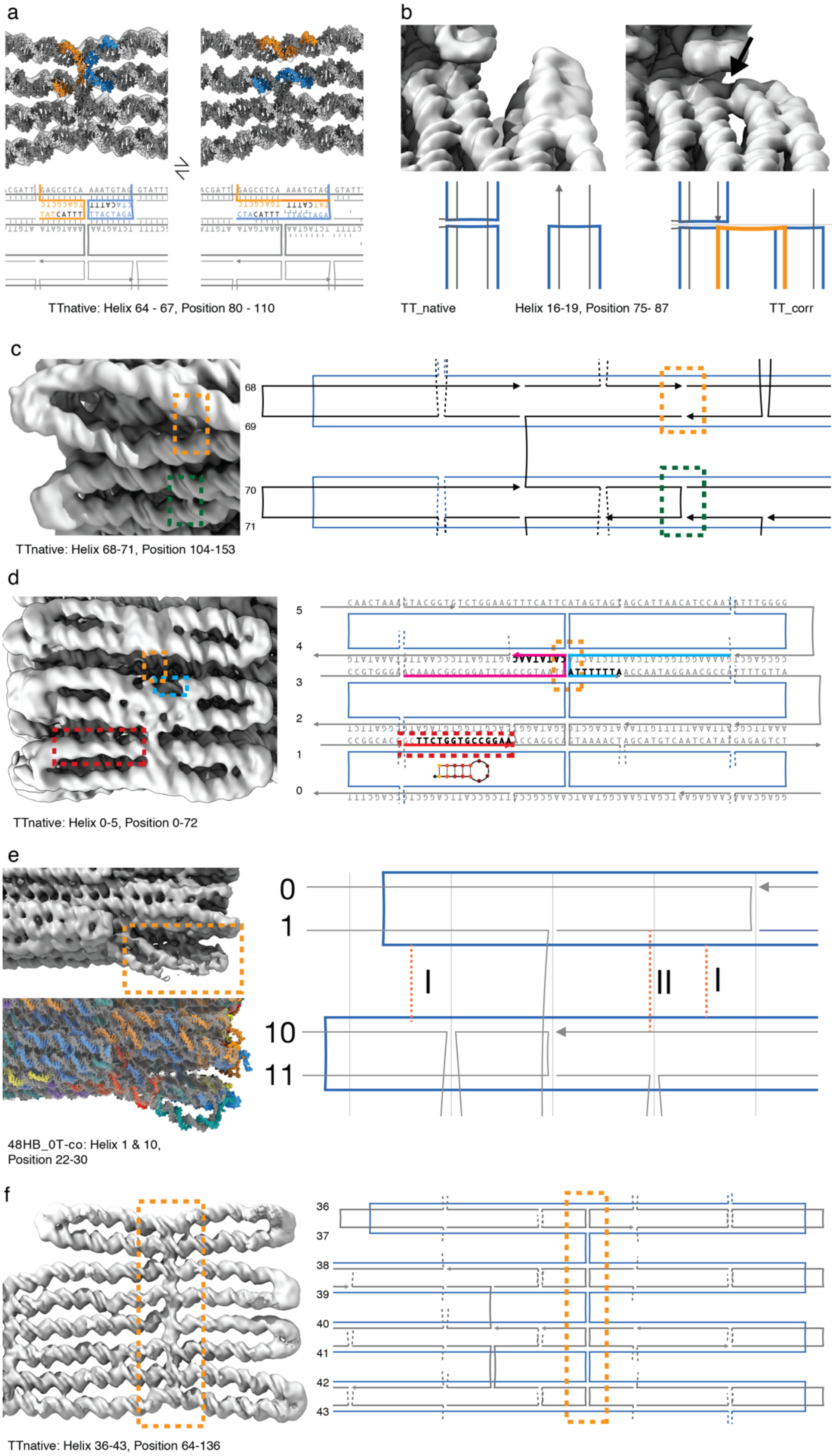

Supplementary Figure 41 | Structural features revealed by detailed inspection of Cryo-EM maps, guided by atomic model annotation. (a) Poorly resolved crossover in the 8x8 domain of the Twisttower. A designed crossover (left diagram) between the two top helices is missing in the Cryo-EM density map at a threshold where the other two crossovers in the stack are well resolved. The strand diagrams show two possible binding conformation of the two staples (orange and blue) due to partial sequence homology. **(b)** Design variation in the Twisttower and the twist-corrected Twisttower.

Left: The two outer helices on the right are bent outwards in the Twisttower. Right: In the twist-corrected variant an additional crossover (orange) connects the end of the helices with the rest of the structure. **(c)** Weakly connected helix at an edge of the 8x8 domain of the Twisttower. The two top helices are spread apart due to not being connected to each other in the shown region. A possible connection position (orange box) was kept unused, unlike for the two helices below (green box). **(d)** Poorly resolved helices in the 6x6 domain in the Twisttower. We attribute the designed but not resolved crossover (orange box) to the partial sequence homology like in a) and high A and T content. The poorly resolved helical domain (blue box) might show an unbound staple segment due to a high A and T content. The staple segment (strand diagram, red box) can form a stable hairpin. This might cause an unbound state of the staple segment from the scaffold that leads to a higher flexibility and thus a lower electron density in this region (cryo-EM map, red box). **(e)** The helix pair is not resolved well due to not realised possible crossovers (I = -scaffold, II = staple). **(f)** Crossover stack in the 8x8 domain of the Twisttower. At the position of the crossover stack (orange box) the helices are tied together.

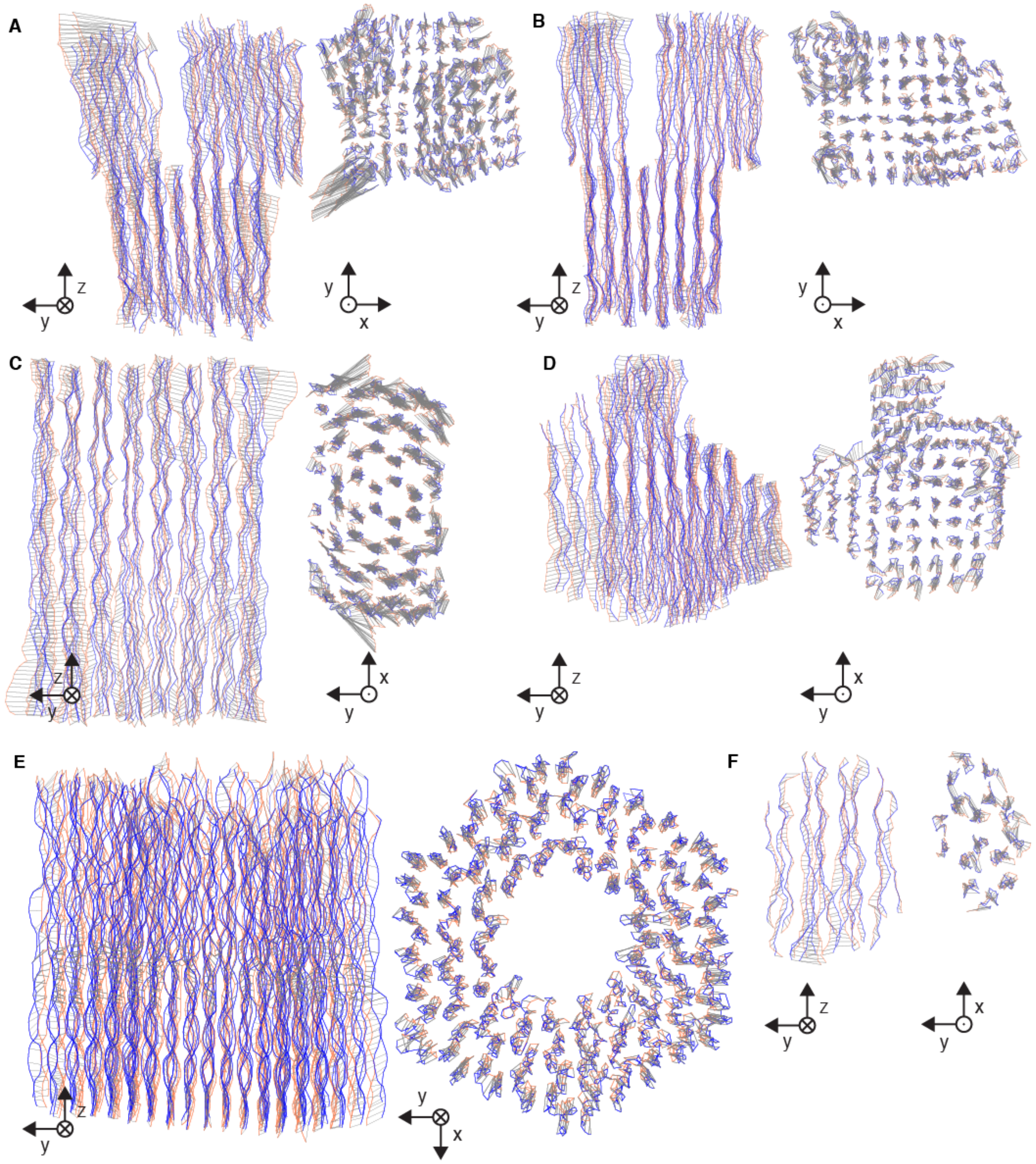

Supplementary Figure 42 | Deviations between ENRG-MD predictions and cryo-EM-map derived atomic models. Orange lines represent helical axes of the atomic models for **(a)** Twisttower, **(b)** twist-corrected Twisttower, **(c)** 48-helix Brick, **(d)** Pointer v2, **(e)** 126-helix bundle **(f)** 16-helix bundle aligned with the relaxed ENRG-MD predicted structures (blue). Connecting lines (grey) depict the displacement at every 2nd base position.

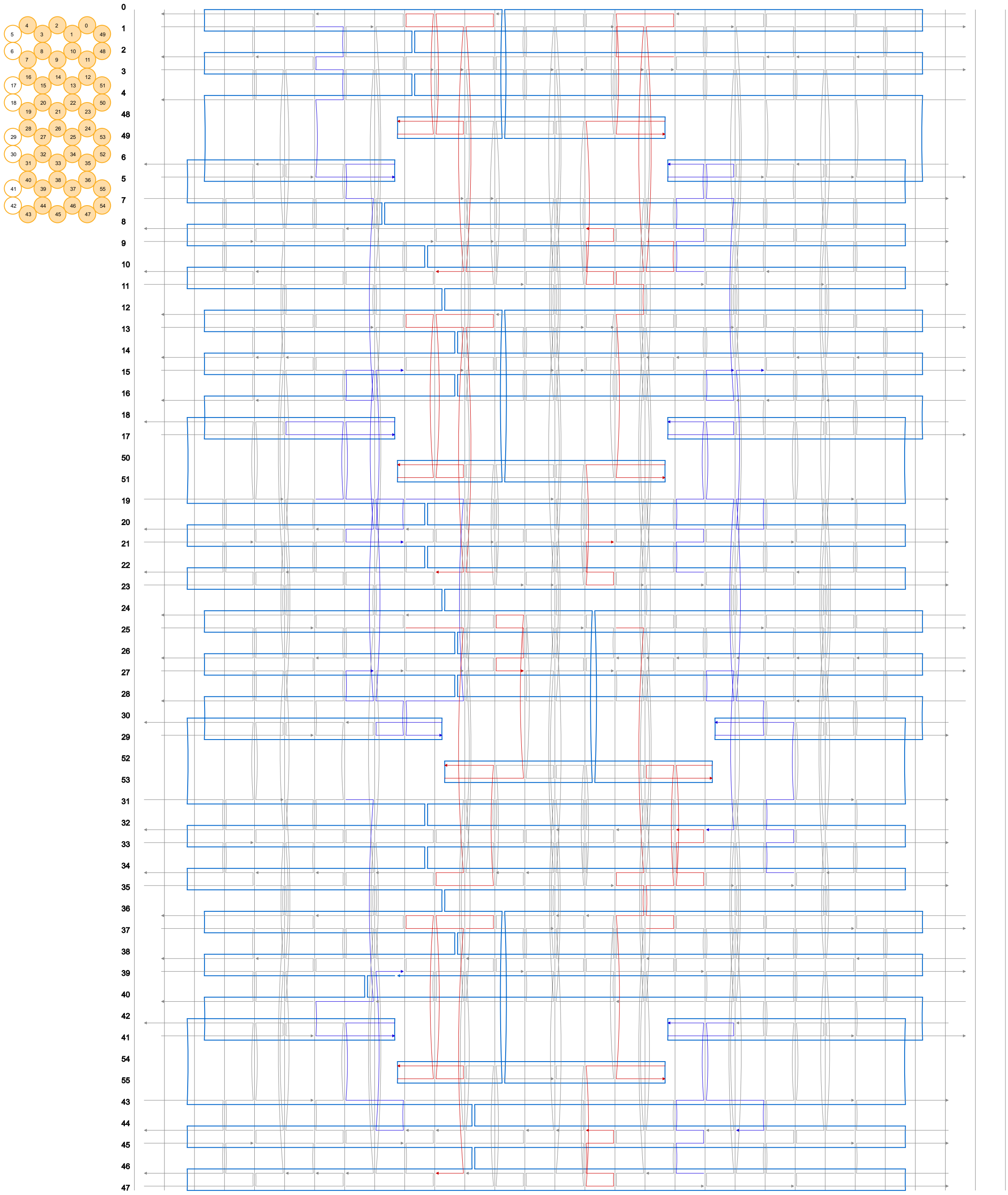

Supplementary Figure 43 | Strand diagram prepared with caDNAno. Object: 48 helix bricks. 0,1,2,4 thymidines were added algorithmically to each staple strand crossover after sequence output.

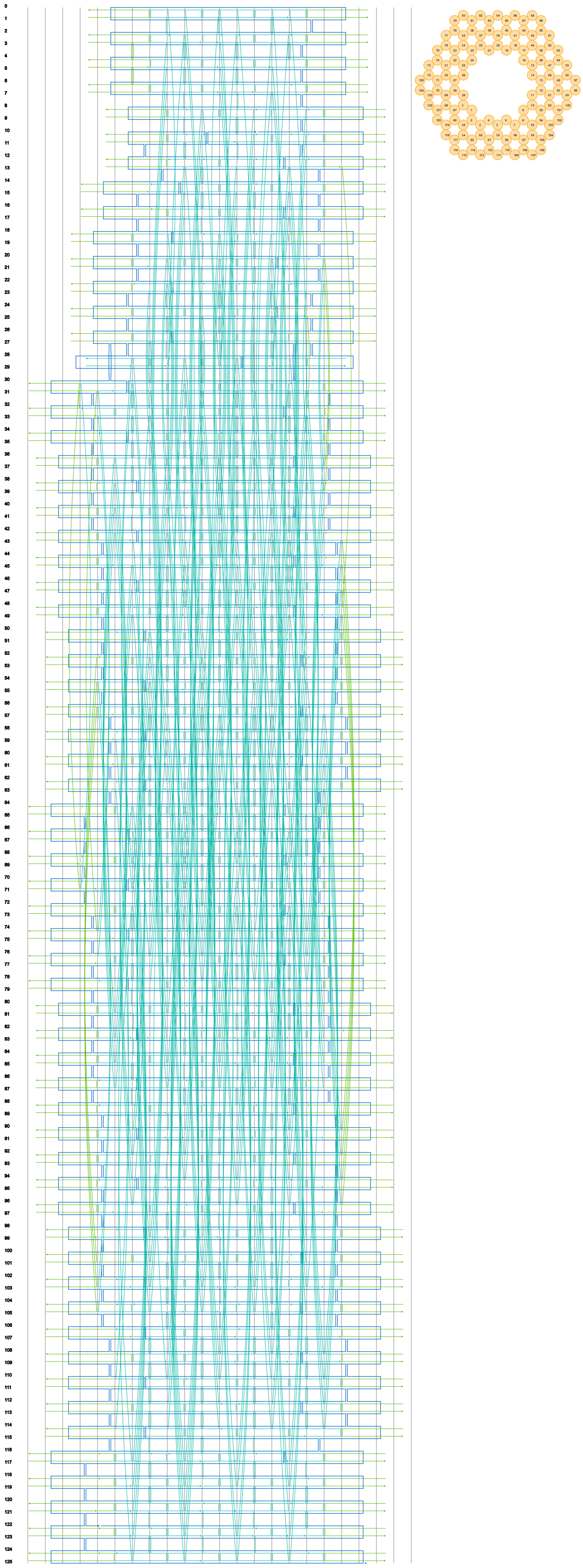

Supplementary Figure 44 | Strand diagram prepared with caDNAno. Object: 126-helix barrel.

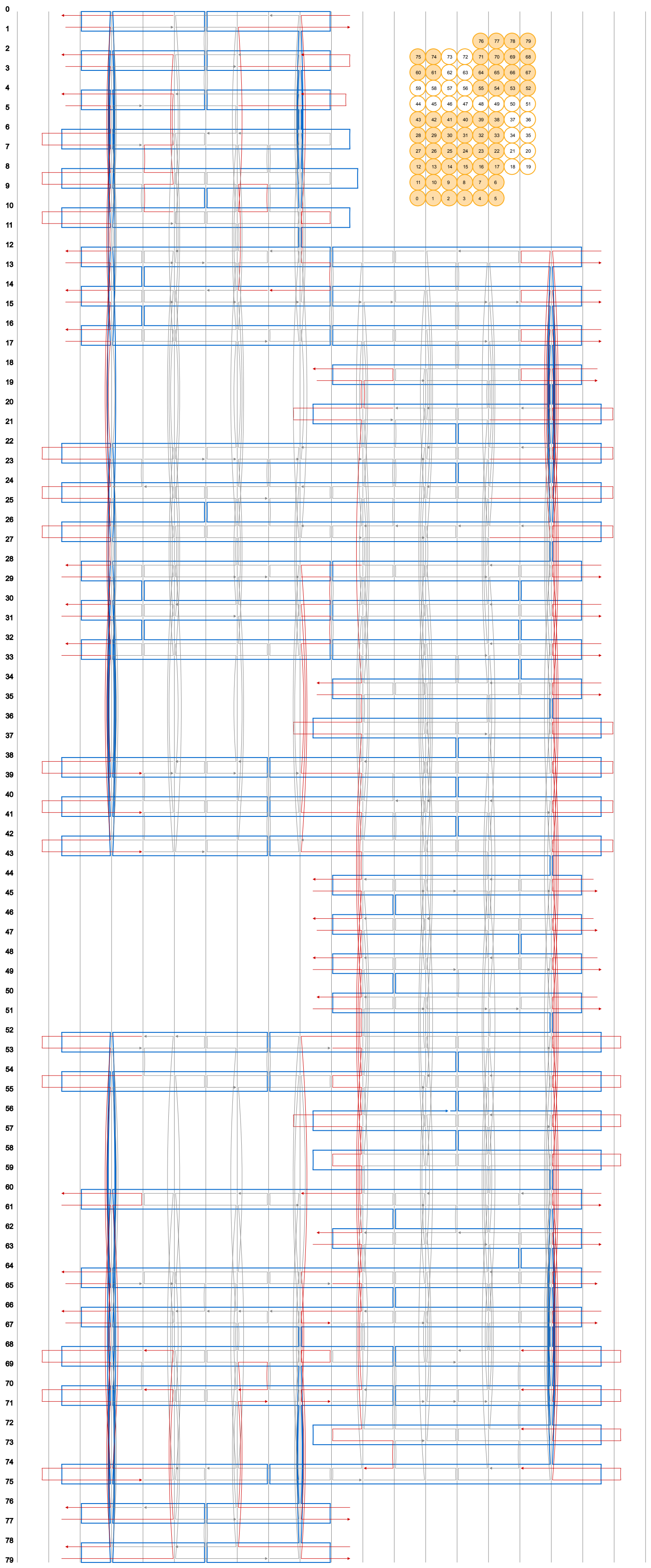

Supplementary Figure 45 | Strand diagram prepared with caDNAno. Object: Twisttower.

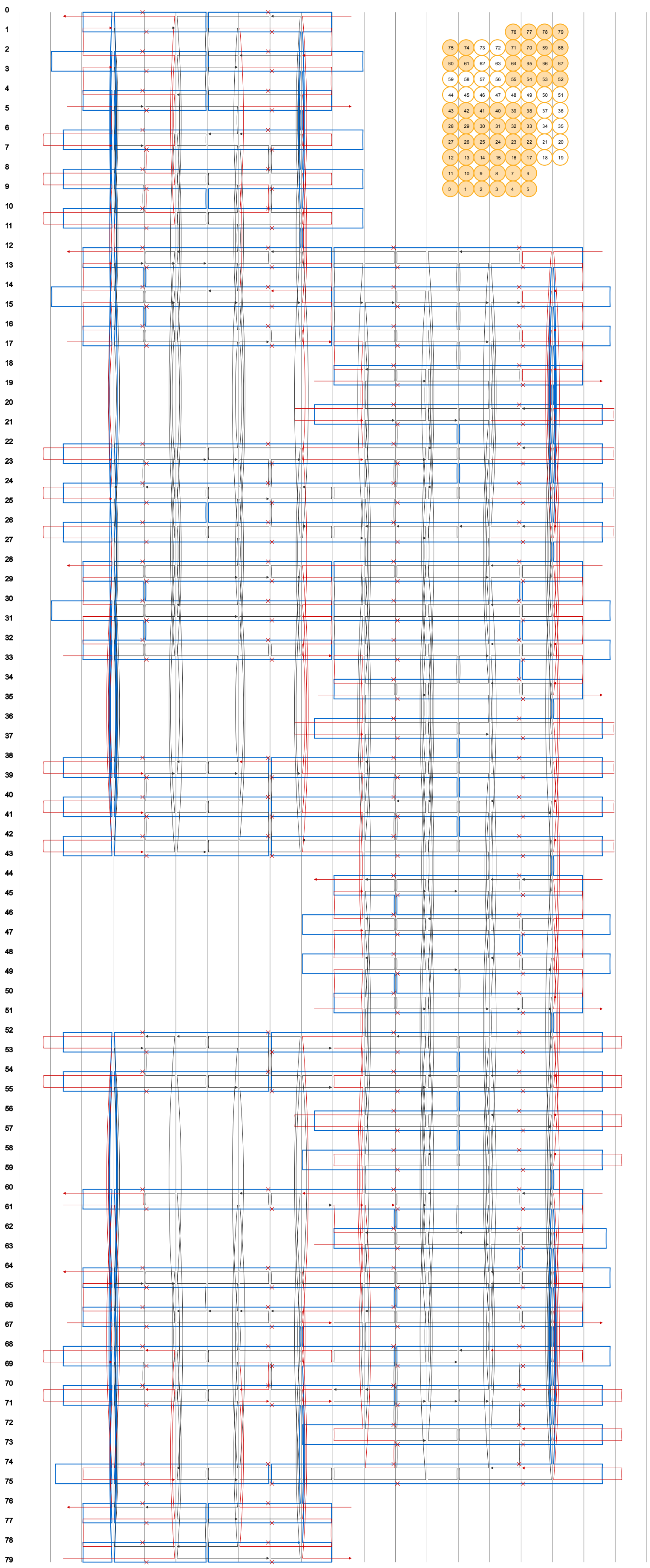

Supplementary Figure 46 | Strand diagram prepared with caDNAno. Object: twist-corrected Twisttower.

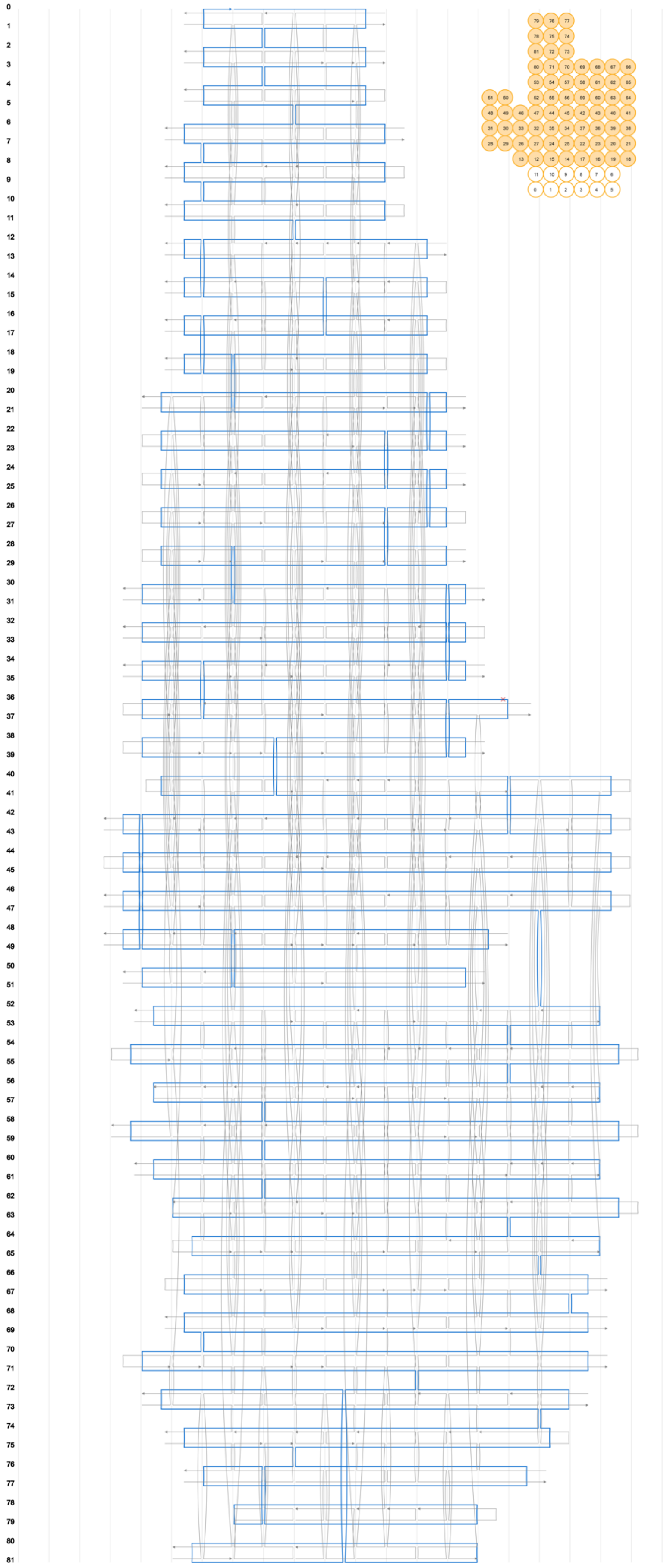

Supplementary Figure 47 | Strand diagram prepared with caDNAno. Object: pointer v2.

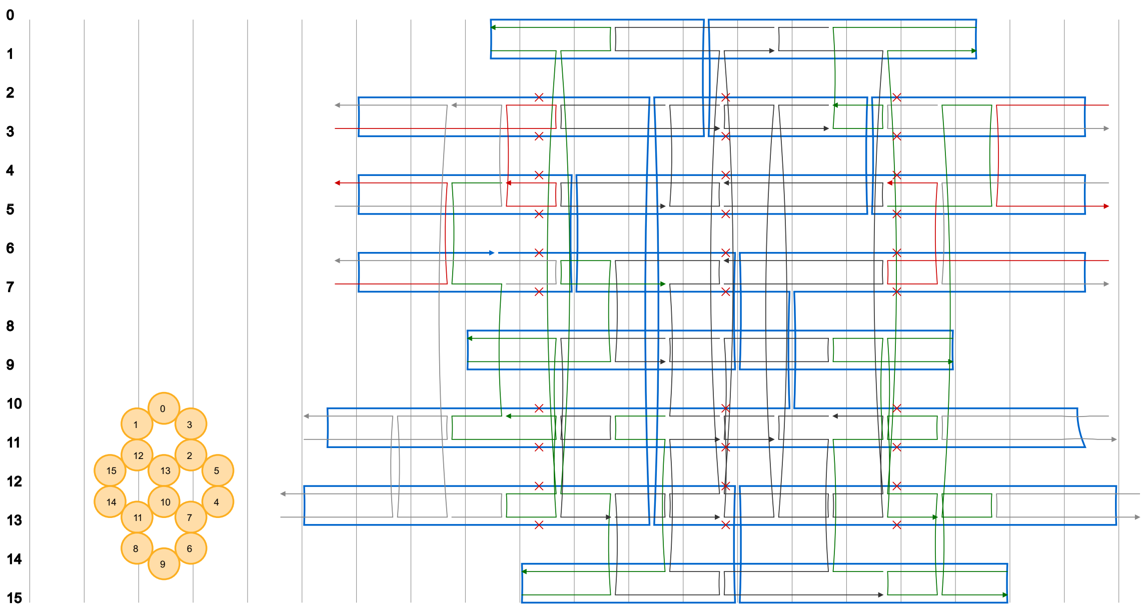

Supplementary Figure 48 | Strand diagram prepared with caDNAno. Object: 16 helix bundle.

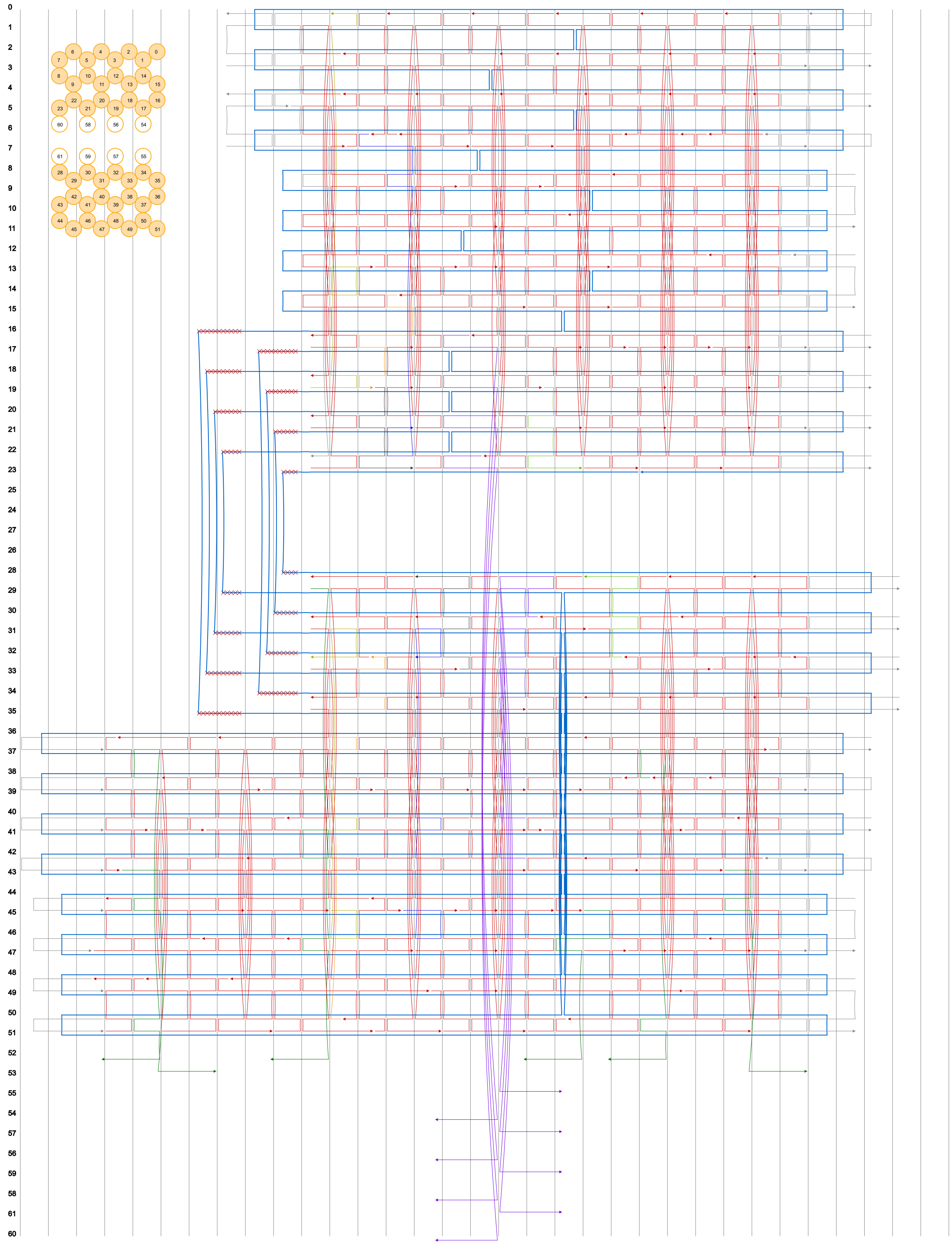

Supplementary Figure 49 | Strand diagram prepared with caDNAno. Object: hinged beam object v1.

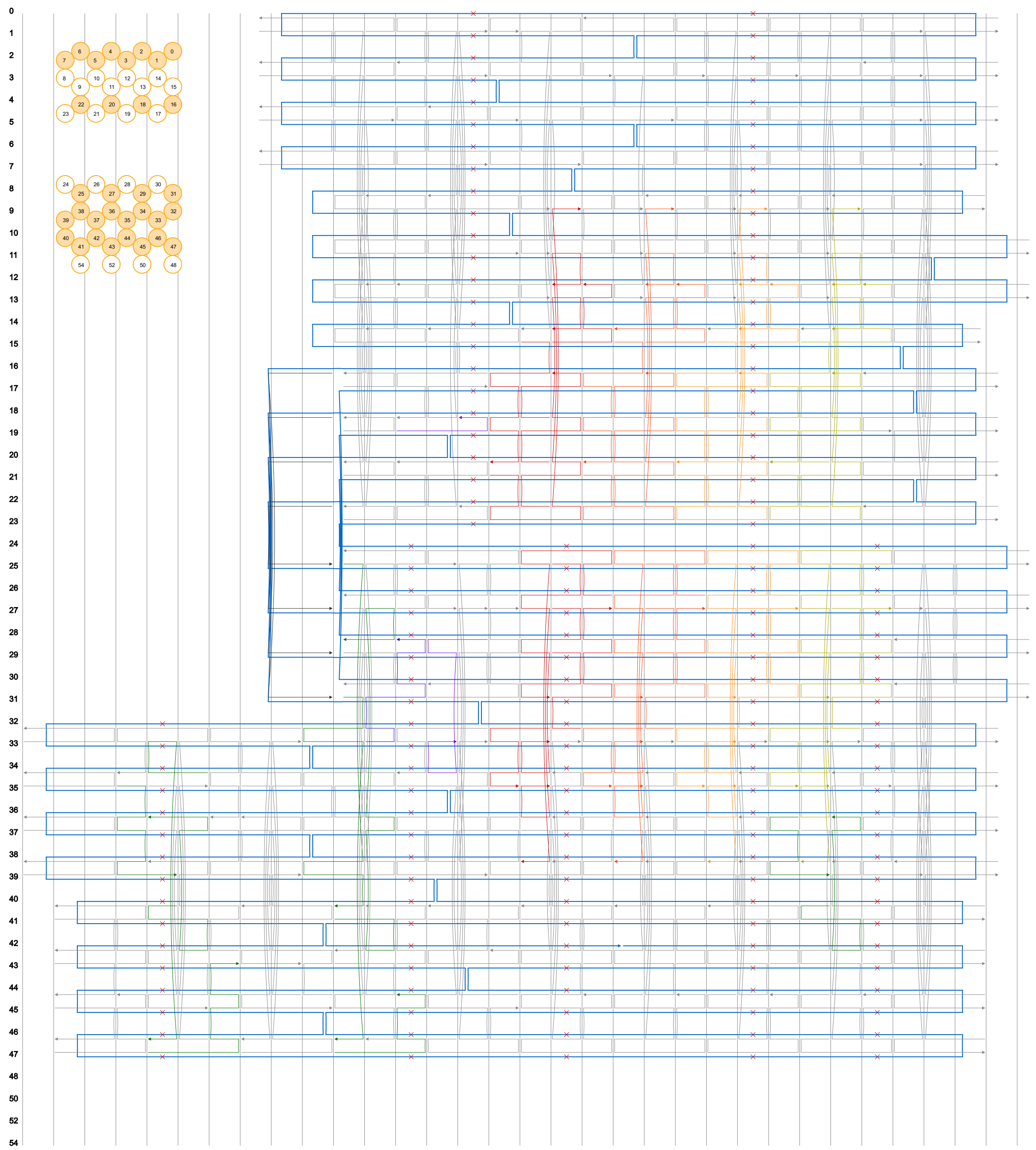

Supplementary Figure 50 | Strand diagram prepared with caDNAno. Object: hinged beam object v2.

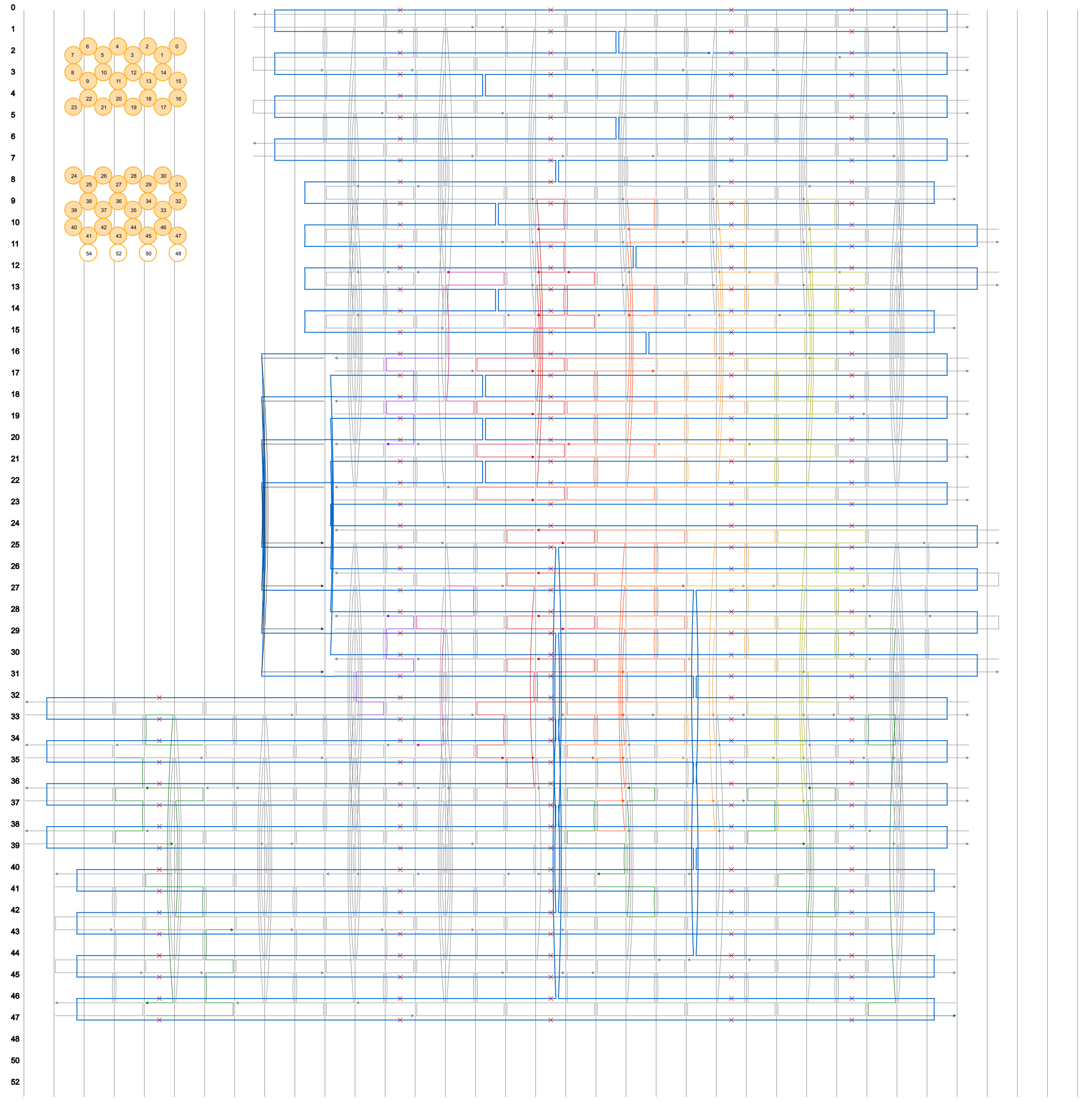

Supplementary Figure 51 | Strand diagram prepared with caDNAno. Object: hinged beam object v3.

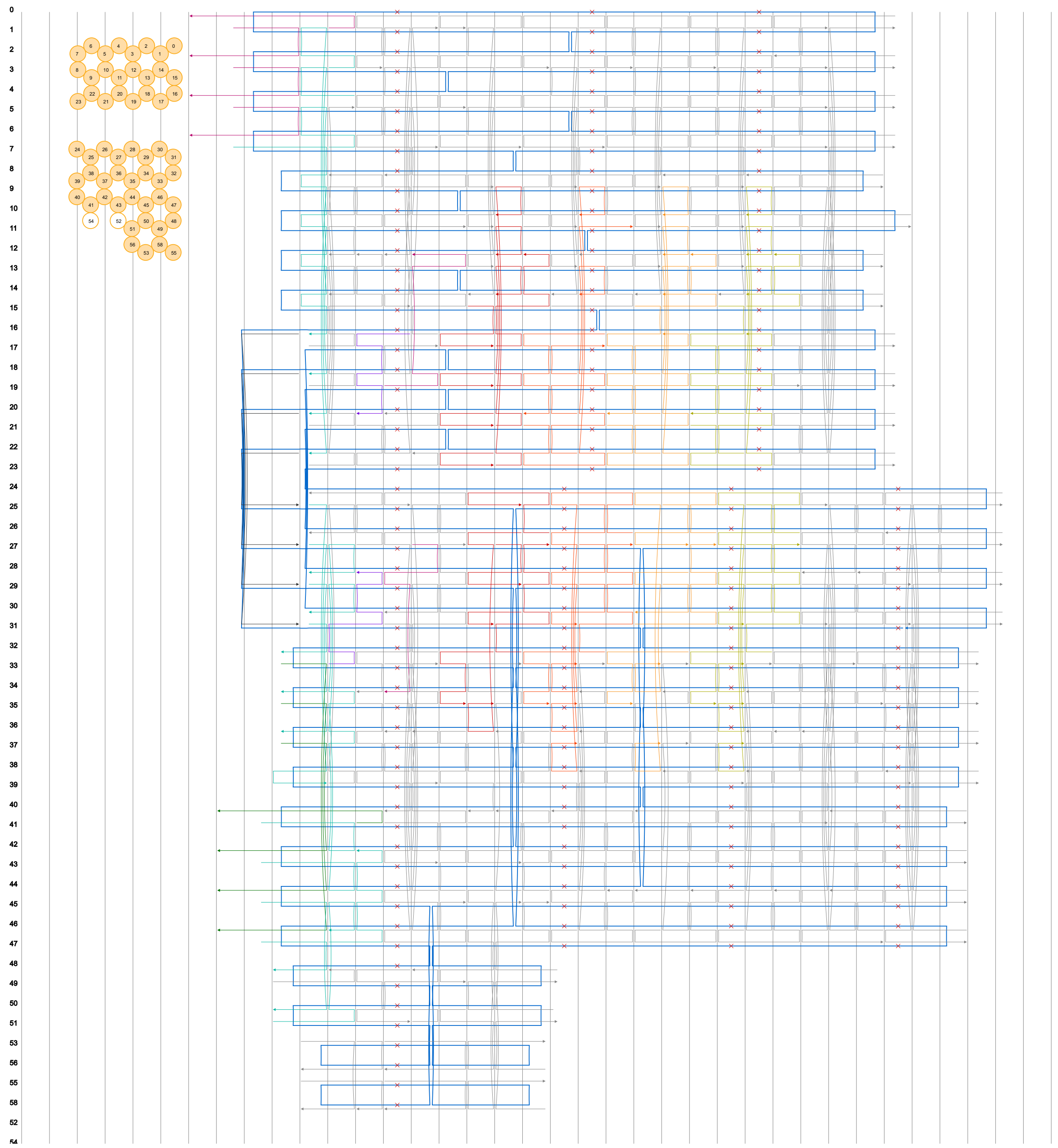

Supplementary Figure 52 | Strand diagram prepared with caDNAno. Object: hinged beam object v4.

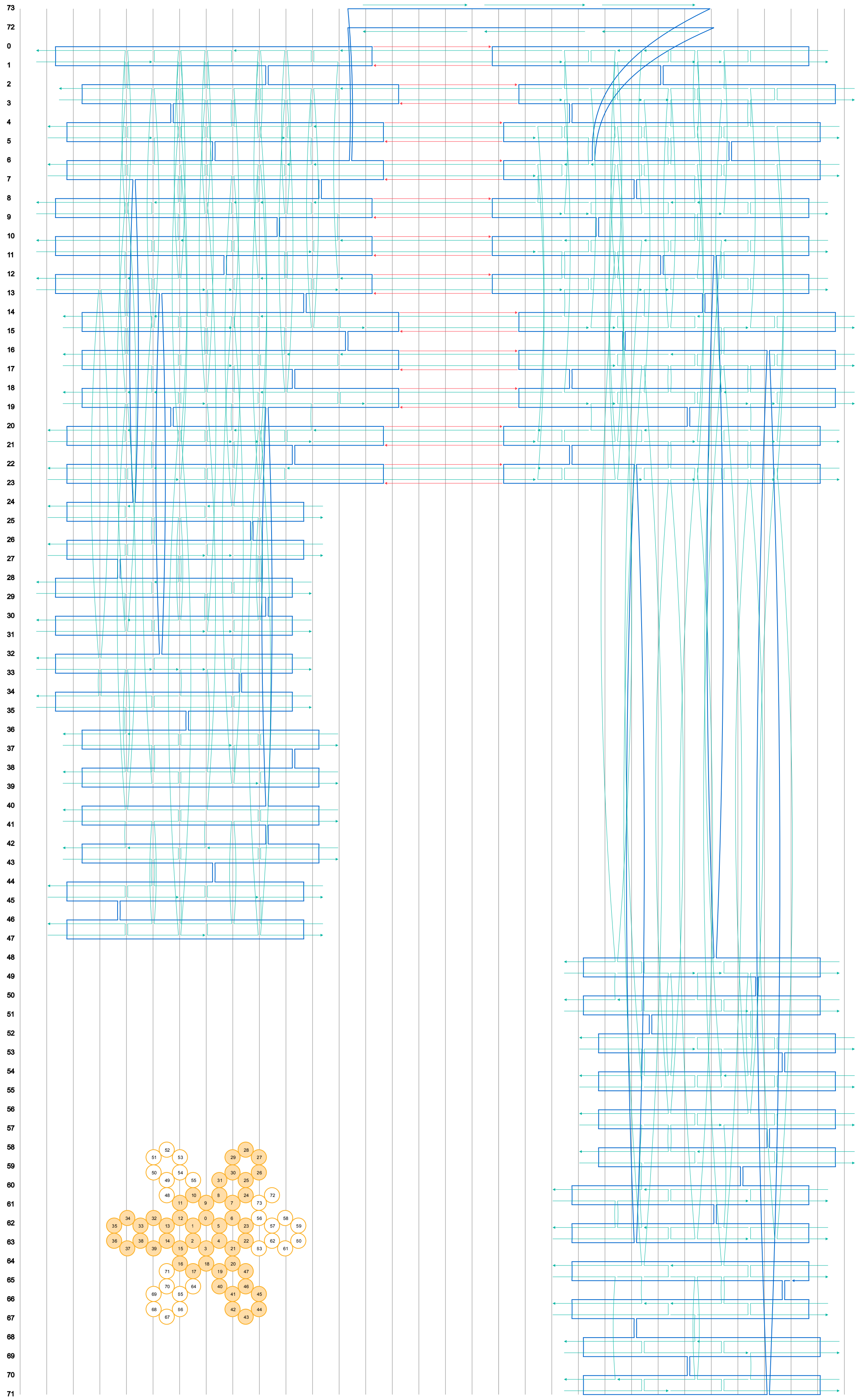

Supplementary Figure 53 | Strand diagram prepared with caDNAno. Object: dumbbell.

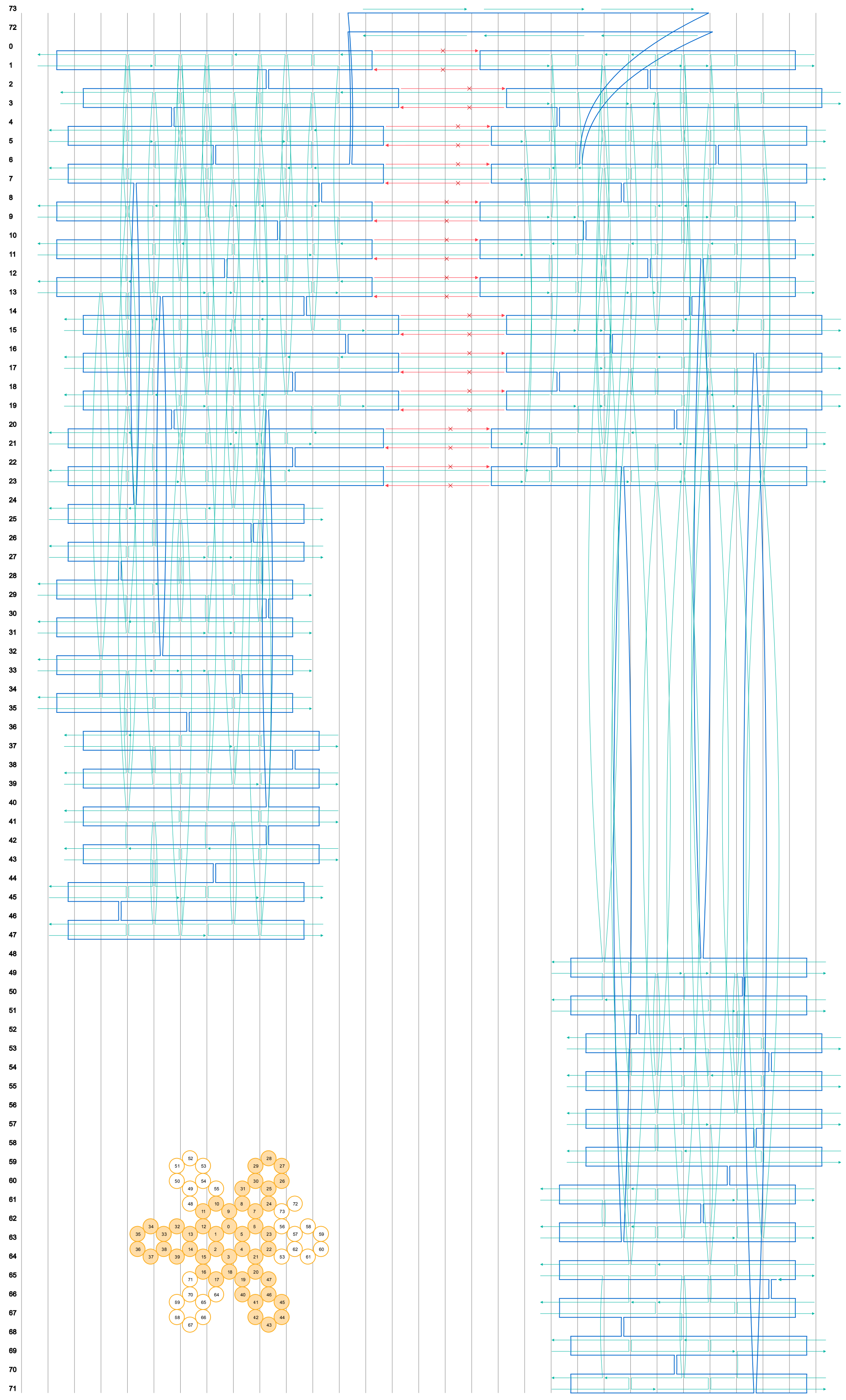

Supplementary Figure 54 | Strand diagram prepared with caDNAno. Object: dumbbell with skips.

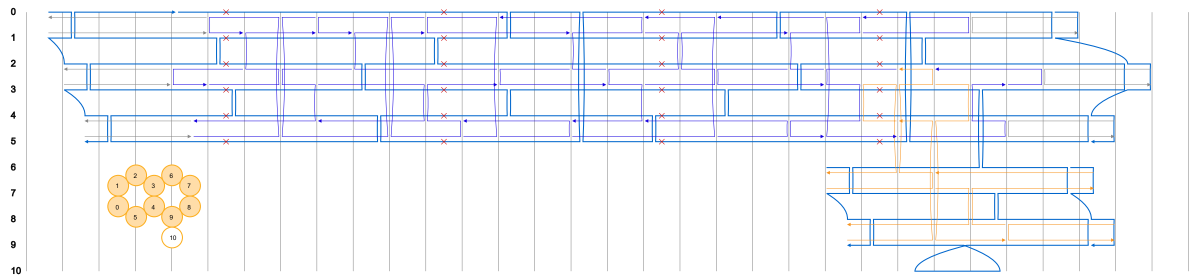

Supplementary Figure 55 | Strand diagram prepared with caDNAno. Object: six-helix-tube v1.

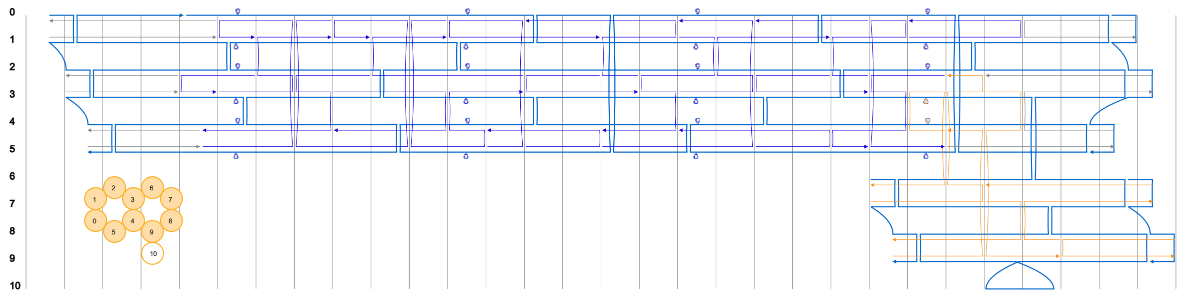

Supplementary Figure 56 | Strand diagram prepared with caDNAno. Object: six-helix-tube v2.

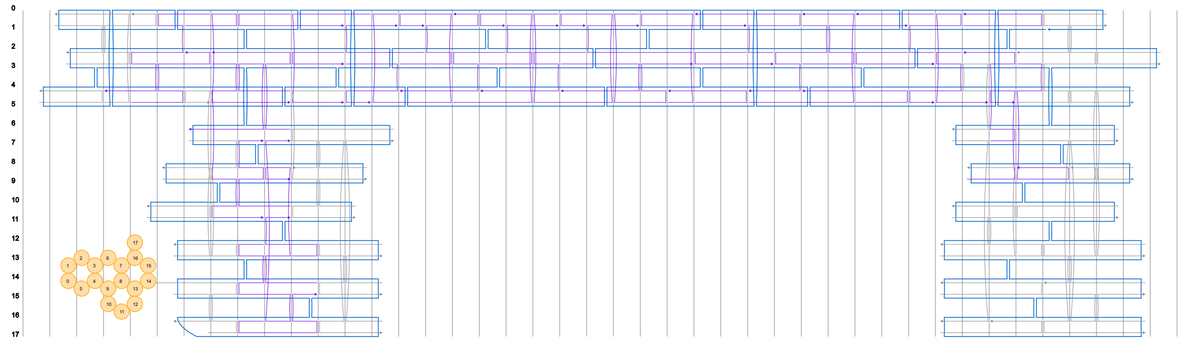

Supplementary Figure 57 | Strand diagram prepared with caDNAno. Object: six-helix-tube v3.

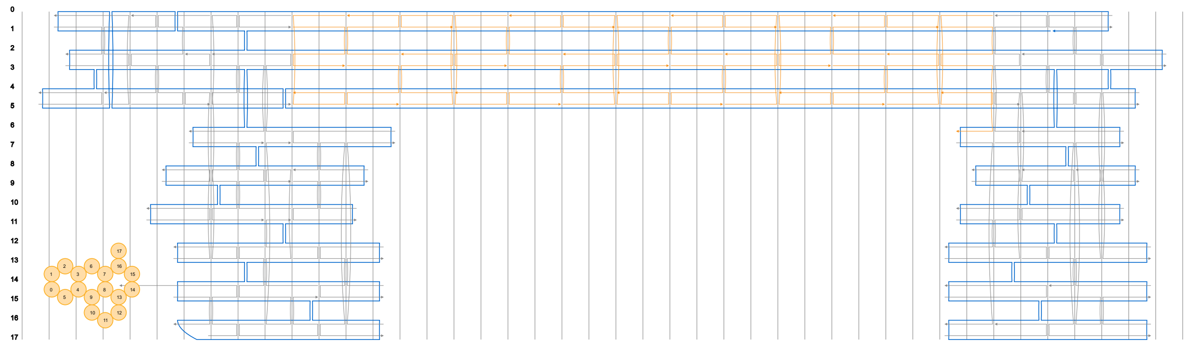

Supplementary Figure 58 | Strand diagram prepared with caDNAno. Object: six-helix-tube v4.

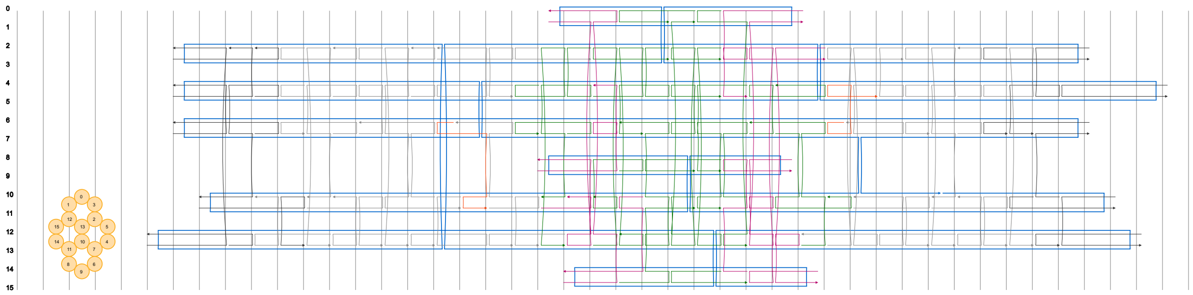

Supplementary Figure 59 | Strand diagram prepared with caDNAno. Object: 10 helix tube.

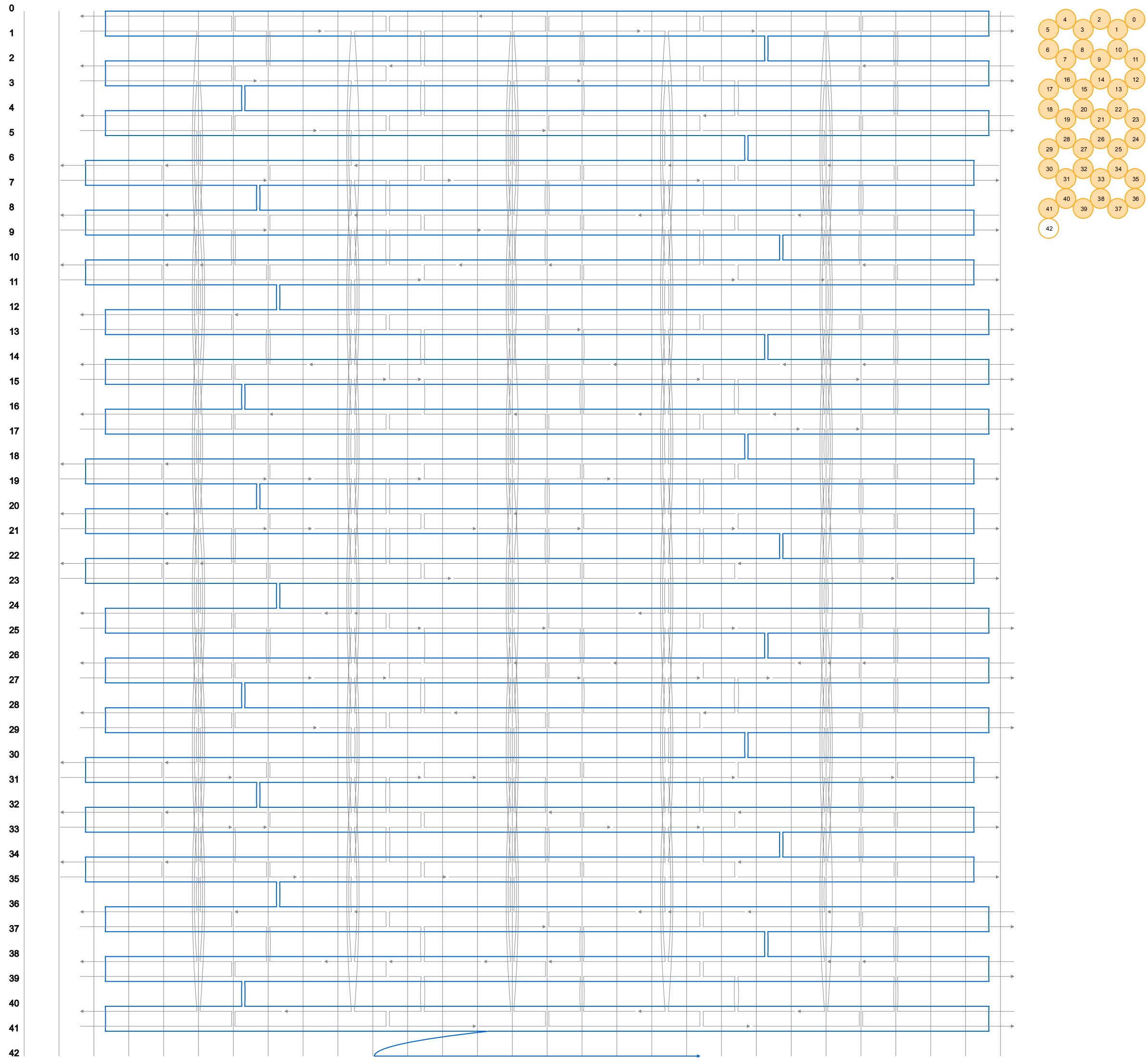

Supplementary Figure 60 | Strand diagram prepared with caDNAno. Object: 42 helix bundle with reduced staple end density.

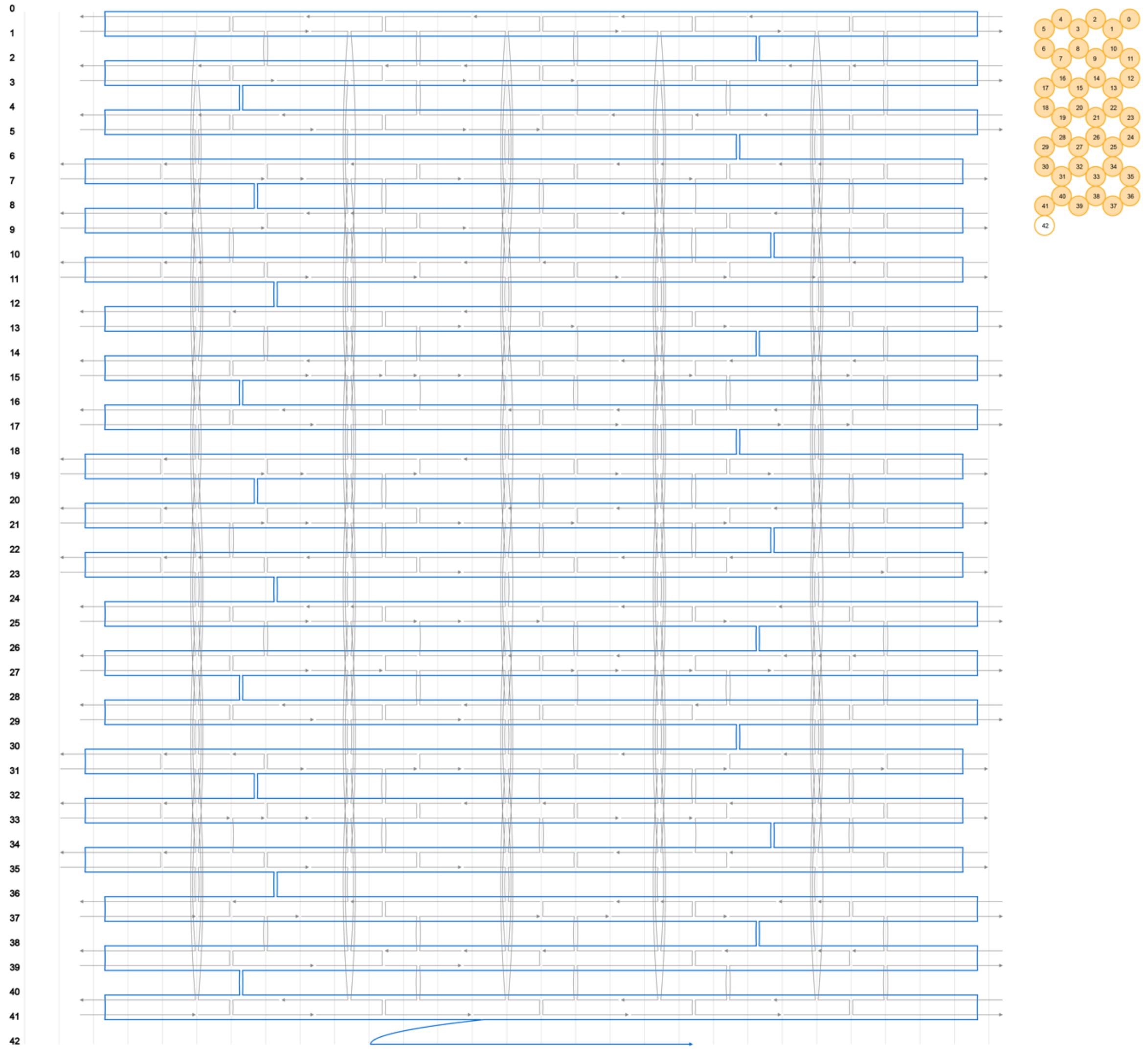

Supplementary Figure 61 | Strand diagram prepared with caDNAno. Object: 42 helix bundle with aligned staple ends. For the 42 helix bundle with aligned staple ends and thymidines at the ends, the thymidines were added manually after sequence output.

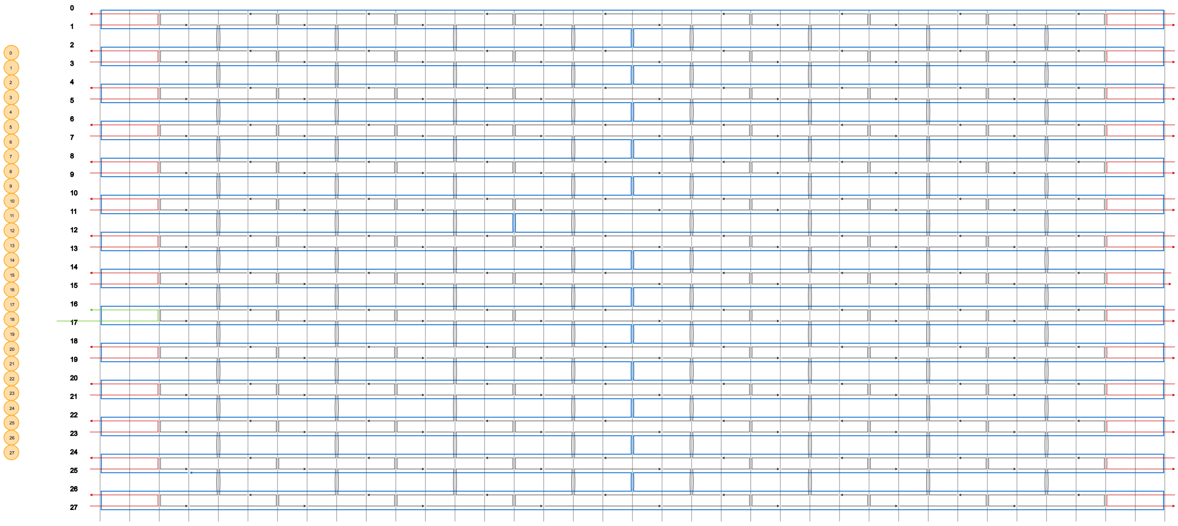

Supplementary Figure 62 | Strand diagram prepared with caDNAno. Object: Rothemund rectangle, default design rules.

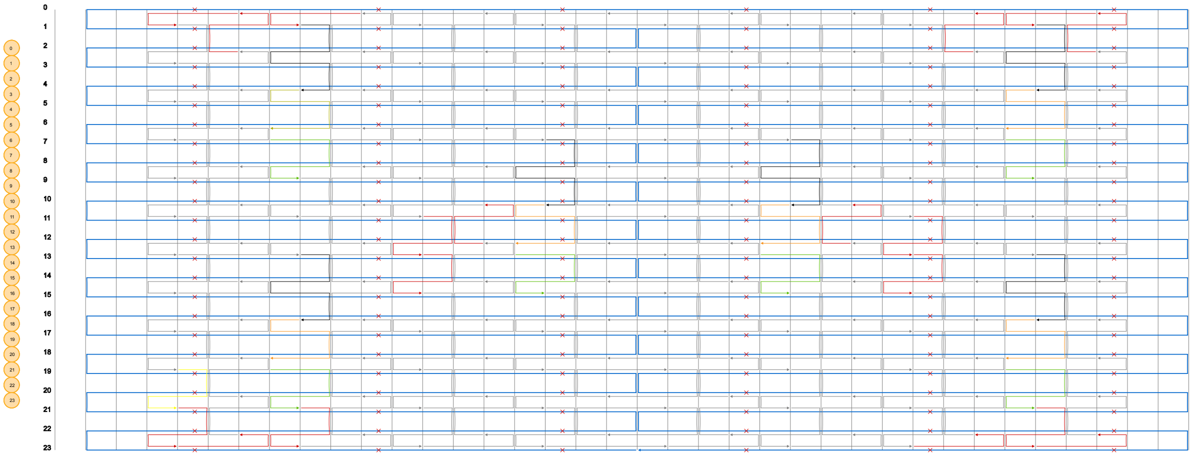

Supplementary Figure 63 | Strand diagram prepared with caDNAno. Object: twist corrected Rothemund rectangle.

SUPPLEMENTARY TABLES

	Structure	Resolution
previous publications	Pointer v1 [1]	12
	52-bp edge-length icosahedron (wireframe) [2]	20
	48 helix bundle connector brick [3]	21
	Nanobarrel [4]	7.5
	Rotor [5]	not stated
this publication	Twisttower	7.4
	Twisttower subpart (2x2x32bp, focussed scanning refinement)	4.3
	Twisttower twist-corrected variant	8.5
	Twisttower twist-corrected variant subpart (4x4 domain)	7.7
	126 helix bundle	9.8
	126 helix bundle subpart (slice)	8.2
	Pointer v2	7.4
	48 helix bundle	9.9
	48 helix bundle subpart (quarter)	9.2
	16 helix bundle	10

Supplementary Table 1 | Overview Resolutions

Structure	Concentration	Grid Type	Magnified Pixel Size [Å]	# used micrographs	#particles in final refinement
**126 helix bundle_dataset_1**	1.2µM	C-Flat 2/1 4C	1.39	2,734	67,819
**126 helix bundle_dataset_2**	1.2µM	C-Flat 2/1 4C	1.39	2,271	54,674
**Dumbbell v1**	1.3µM	C-Flat 1.2/1.3 4C	1.79	220	2,607
**Dumbbell v2**	1.0µM	C-Flat 1.2/1.3 4C	3.71	736	18,656
**Twisttower_twist-corrected variant_dataset 1**	0.9µM	C-Flat 2/1 4C	1.39	876	19,815
**Twisttower_twist-corrected_variant dataset 2**	0.9µM	C-Flat 2/1 4C	1.39	5,035	75,019
**Twisttower dataset_1**	1.5µM	C-Flat 2/1 4C	1.39	2,05	32,548
**Twisttower dataset_2**	1.0µM	C-Flat 2/1 4C	1.39	4,866	80,415
**Twisttower_dataset_3**	2.3µM	C-Flat 2/1 4C	1.39	1496	46,738
**Twisttower_ dataset_4**	2.0µM	C-Flat 2/1 4C	1.39	3623	172,78
**Twisttower_ dataset_5**	1.75µM	C-Flat 2/1 4C	1.39	9244	350,111
**48 helix bundle brick**	500nM	C-Flat 2/1 4C	1.79	1491	31931
**48 helix bundle brick with one T at all staple crossovers**	200nM	C-Flat 2/1 4C	1.79	3893	59019
**48 helix bundle brick with two Ts at all staple crossovers**	500nM	C-Flat 2/1 4C	1.79	2600	30105
**48 helix bundle brick with four Ts at all staple crossovers**	500nM	C-Flat 2/1 4C	1.79	1639	14966
**hinged-beam-like object v1**	500nM	C-Flat 1.2/1.3 4C	2.3 (Phaseplate)	1435	21839
**hinged-beam-like object v2**	500nM	C-Flat 1.2/1.3 4C	2.3 (Phaseplate)	1521	5920
**hinged-beam-like object v3**	500nM	C-Flat 1.2/1.3 4C	2.3 (Phaseplate)	449	4530
**hinged-beam-like object v4**	1.0µM	C-Flat 2/1 4C	1.79	702	17924
**42 helix bundle with aligned staple breaks**	700nM	C-Flat 2/1 4C	2.3	1790	31128
**42 helix bundle with aligned staple breaks UV point welded**	500nM	C-Flat 2/1 4C	2.3	1582	28281
**42 helix bundle with aligned staple breaks and 1T at termini**	500nM	C-Flat 2/1 4C	1.79	1536	21674
**42 helix bundle with aligned staple breaks and 1T at termini 1T UV point welded**	500nM	C-Flat 2/1 4C	1.79	2080	15754
**42 helix bundle with reduced staple density**	500nM	C-Flat 1.2/1.3 4C	2.3	409	7553
**42 helix bundle with reduced staple density UV point welded**	500nM	C-Flat 2/1 4C	2.3	1228	20440
**10 helix tube**	1.2µM	C-Flat 2/1 4C	2.3 (Phaseplate)	421	3953
**16 helix bundle**	20nM	C-Flat 1.2/1.3 4C GO coated	2.3	480	44605
**6 helix tube V1**	1.4µM	C-Flat 2/1 4C	2.3	744	29932
**6 helix tube V2**	1.4µM	C-Flat 2/1 4C	1.79	989	50785
**6 helix tube V3**	900nM	C-Flat 2/1 4C	1.79	358	8968
**6 helix tube V4**	400nM	C-Flat 1.2/1.3 4C	2.3	2503	9763
**rectangle variant with 8064 scaffold**	1.6µM	C-Flat 2/1 4C	1.39	1167	9708
**rectangle variant twist corrected**	0.55µM	C-Flat 1.2/1.3 4C	1.39 (Phaseplate)	3365	7415
**Pointer v2**	50nM	Quantifoil gold 1.2/1.3 4C GO coated	1.79	2381	281527

Supplementary Table 2| Cryo grid preparation and data acquisition details.

	Overall Resolution [Å]	Masked CCC Fitted Model	RMSD ENRG-MD vs Fitted Model [Å]
**Twisttower**	7.0	0.927	16.455
**Twisttower twist corrected variant**	8.1	0.941	10.987
**48 helix bundle Brick**	9.9	0.932	14.927
**16 helix bundle**	10.0	0.940	10.307
**Pointer V2**	7.4	0.874	9.656
**126 helix bundle**	8.8	0.909	13.560

Supplementary Table 3 | Cross-correlation coefficients for each of the six fitted atomic models and deviation from ENRG-MD initial models. Cryo-maps have been masked, simulated maps have been created at the reported resolution of the experimental map. For each model a reference ENRG-MD simulation has been performed and its RMSD to the fitted model calculated.

STRUCTURE	SCAFFOLD TYPE(S) AND CONCENTRATION	#OLIGONUCLEOTIDES, CONCENTRATION	MAGNESIUM IN FOLDING BUFFER	ANNEALING RAMP	PURIFICATION METHOD	CONCENTRATION METHOD
**126 HELIX BUNDLE**	7560 @ 20nM, Csv2 @ 20nM	#453, each @200nM	25mM	60-40°C, 3h/°C	filter	filter
**TWISTTOWER SAMPLE 1 (DATASET 1)**	8064 @ 50nM	#194, each @ 200nM	20mM	56-53°C, 1h/°C	filter	filter
**SAMPLE 2 (DATASET 2-4)**	8064 @ 50nM	#194, each @ 200nM	20mM	54-53°C, 2h/°C	filter	filter
**SAMPLE 3 (DATASET 5)**	8064 @ 50nM	#194, each @ 200nM	20mM	54-53°C, 2h/°C	filter	filter
**TWISTTOWER TWIST CORRECTED VARIANT**	8064 @ 20nM	#191, each @ 200nM	20mM	60-44°C, 1h/°C	filter	filter
**DUMBELL V1**	7249 @ 50nM	#204, each @ 200nM	25mM	60-44°C, 1h/°C	filter	filter
**DUMBELL V2**	7249 @ 50nM	#204, each @ 200nM	25mM	60-44°C, 1h/°C	filter	filter
**48 HELIX BUNDLE BRICK**	8064 @50nM	#211, each @ 200nM	20mM	60-44°C, 1h/°C	PEG	filter
**48 HELIX BUNDLE BRICK WITH ONE T AT ALL STAPLE CROSSOVERS**	8064 @50nM	#211, each @ 200nM	20mM	60-44°C, 1h/°C	PEG	filter
**48 HELIX BUNDLE BRICK WITH TWO TS AT ALL STAPLE CROSSOVERS**	8064 @50nM	#211, each @ 200nM	20mM	60-44°C, 2h/°C	PEG	filter
**48 HELIX BUNDLE BRICK WITH FOUR TS AT ALL STAPLE CROSSOVERS**	8064 @50nM	#211, each @ 200nM	20mM	60-44°C, 1h/°C	PEG	filter
**HINGED-BEAM-LIKE OBJECT V1**	7560 @50nM	#188, each @ 200nM	20mM	58-56;52-50°C, 2h/°C	PEG	filter
**HINGED-BEAM-LIKE OBJECT V2**	8064 @50nM	#210, each @ 200nM	20mM	60-44°C, 1h/°C	PEG	filter
**HINGED-BEAM-LIKE OBJECT V3**	8064 @50nM	#219, each @ 200nM	20mM	60-44°C, 1h/°C	PEG	filter
**HINGED-BEAM-LIKE OBJECT V4**	8064 @50nM	#209, each @ 200nM	20mM	60-44°C, 1h/°C	PEG	filter
**42 HELIX BUNDLE WITH ALIGNED STAPLE BREAKS**	7560 @50nM	#213, each @ 200nM	20mM	60-44°C, 1h/°C	PEG	filter
**42 HELIX BUNDLE WITH ALIGNED STAPLE BREAKS AND ONE T AT TERMINI**	7560 @50nM	#219, each @ 200nM	20mM	60-44°C, 1h/°C	PEG	filter
**42 HELIX BUNDLE WITH REDUCED STAPLE DENSITY**	7560 @50nM	#161, each @ 200nM	20mM	60-44°C, 1h/°C	PEG	filter
**10 HELIX tube**	2873 @50nM	#99, each @ 200nM	15mM	60-45°C, 1h/°C	PEG	filter
**16 HELIX BUNDLE**	1317 @50nM	#46, each @ 200nM	15mM	60-45°C, 1h/°C	PEG	filter
**6 HELIX tube V1**	1317 @50nM	#39, each @ 200nM	15mM	60-45°C, 1h/°C	PEG	filter
**6 HELIX tube V2**	1317 @50nM	#40, each @ 200nM	15mM	60-45°C, 1h/°C	PEG	filter
**6 HELIX tube V3**	2873 @50nM	#79, each @ 200nM	15mM	60-45°C, 1h/°C	PEG	filter
**6 HELIX tube V4**	2873 @20nM	#77, each @ 200nM	15mM	60-45°C, 1h/°C	HPLC	filter
**RECTANGLE VARIANT WITH 8064 SCAFFOLD**	8064 @40nM	#188, each @266nM	20mM	64-54°C, 5min/°C + 55-40°C, 1min/°C	filter	filter
**RECTANGLE VARIANT TWIST CORRECTED**	7249 @50nM	#186, each @ 200nM	12.5mM	65-45°C, 2min/°C	PEG	filter
**POINTER V2**	7249 @50nM	#202, each @ 200nM	20mM	56-44°C, 3h/°C	filter	filter

Supplementary Table 4 | DNA origami self-assembly reaction details.

STRUCTURE	EMDB ID	PDB ID
Twisttower	EMD-11379	7ARV
Twisttower twist corrected variant	EMD-11378	7ARY
pointer object v2	EMD-11881	7ARE
126 helix bundle	EMD-11170	7AS5
16 helix bundle	EMD-11367	7ARQ
48 helix bundle brick	EMD-11387	7ART
48 helix bundle brick with 1t at staple crossovers	EMD-11343	
48 helix bundle brick with 2t at staple crossovers	EMD-11344	
48 helix bundle brick with 4t at staple crossovers	EMD-11345	
10 helix bundle	EMD-11355	
6 helix tube v1	EMD-11351	
6 helix tube v2	EMD-11352	
6 helix tube v3	EMD-11353	
6 helix tube v4	EMD-11354	
hinged beam like object v1	EMD-11346	
hinged beam like object v2	EMD-11348	
hinged beam like object v3	EMD-11349	
hinged beam like object v4	EMD-11350	
dumbbell_1	EMD-11159	
dumbbell_2	EMD-11168	
42 helix bundle with aligned staple breaks	EMD-11294	
42 helix bundle with aligned staple breaks after UV illumination	EMD-10993	
42 helix bundle with aligned staple breaks and 1t at staple termini	EMD-11295	
42 helix bundle with aligned staple breaks and 1t at staple termini after UV illumination	EMD-11296	
42 helix bundle with reduced staple density	EMD-11297	
42 helix bundle with reduced staple density after UV illumination	EMD-11298	

Supplementary Table 5 | EMDB and PDB codes

SUPPLEMENTARY REFERENCES

1. X. C. Bai, T. G. Martin, S. H. Scheres, H. Dietz, Cryo-EM structure of a 3D DNA-origami object. *Proceedings of the National Academy of Sciences of the United States of America* **109**, 20012-20017 (2012)
2. R. Veneziano *et al*., Designer nanoscale DNA assemblies programmed from the top down. *Science* **352**, 1534-1542 (2016).
3. K. F. Wagenbauer, C. Sigl, H. Dietz Gigadalton-scale Shape-Programmable DNA Assemblies. *Nature* **552**, pages78–83 (2017)
4. Y. Dong *et al*., Folding DNA into a Lipid-Conjugated Nanobarrel for Controlled Reconstitution. *Angewandte Chemie* **57**, 2072-2076 (2018)
5. Y. Ahmadi et al., The Brownian and Flow-Driven Rotational Dynamics of a Multicomponent DNA Origami-Based Rotor. *Small* **16**, 2001855 (2020)
