# Supplementary material for: Revealing the structures of megadalton-scale DNA complexes with nucleotide resolution
Source: Nat Commun. 2020 Dec 4;11:6229. doi: 10.1038/s41467-020-20020-7 (PMC7718922; doi:10.1038/s41467-020-20020-7)
Supplement: Supplementary file 9 — Reporting Summary [file 41467_2020_20020_MOESM9_ESM.pdf]

nature research

Corresponding author(s): Prof. Dr. Hendrik Dietz

Last updated by author(s): Jul 22, 2020

Reporting Summary

Nature Research wishes to improve the reproducibility of the work that we publish. This form provides structure for consistency and transparency in reporting. For further information on Nature Research policies, see Authors & Referees and the Editorial Policy Checklist .

Statistics

For all statistical analyses, confirm that the following items are present in the figure legend, table legend, main text, or Methods section.

n/a	Confirmed	
[ ]	[x]	The exact sample size (n) for each experimental group/condition, given as a discrete number and unit of measurement
[ ]	[x]	A statement on whether measurements were taken from distinct samples or whether the same sample was measured repeatedly
[x]	[ ]	The statistical test(s) used AND whether they are one- or two-sided<br>*Only common tests should be described solely by name; describe more complex techniques in the Methods section.*
[x]	[ ]	A description of all covariates tested
[x]	[ ]	A description of any assumptions or corrections, such as tests of normality and adjustment for multiple comparisons
[x]	[ ]	A full description of the statistical parameters including central tendency (e.g. means) or other basic estimates (e.g. regression coefficient) AND variation (e.g. standard deviation) or associated estimates of uncertainty (e.g. confidence intervals)
[x]	[ ]	For null hypothesis testing, the test statistic (e.g. F, t, r) with confidence intervals, effect sizes, degrees of freedom and P value noted<br>*Give P values as exact values whenever suitable.*
[x]	[ ]	For Bayesian analysis, information on the choice of priors and Markov chain Monte Carlo settings
[x]	[ ]	For hierarchical and complex designs, identification of the appropriate level for tests and full reporting of outcomes
[x]	[ ]	Estimates of effect sizes (e.g. Cohen's d, Pearson's r), indicating how they were calculated

Our web collection on statistics for biologists contains articles on many of the points above.

Software and code

Policy information about availability of computer code

Data collection	EPU 1.2 up to 2.6 (Thermo Fisher)
Data analysis	Relion 2.0 up to 3.1beta, CTFFIND 3, CTFFIND 4.0.7 up to 4.1.14, MotionCorr 2, Cryolo 1.0 up to 1.5, CaDNAno 2.3.0, NAMD 2.12_Linux, CHARMM36 forcefield, Python 3.6.7, Python packages: mrcfile 1.1.2, mdanalysis 0.20.1, autodesk/nanodesign: https://github.com/elija-feigl/nanodesign_dietz, Jupyter-core 4.6.3, VMD 1.9.3 MacOS X OpenGL (32-bit Intel x86), Vmd packages: mdff_0.5 and volutil_1.3 https://github.com/elija-feigl/FitViewer, UCSF chimera 1.13.0 & 1.14.0

For manuscripts utilizing custom algorithms or software that are central to the research but not yet described in published literature, software must be made available to editors/reviewers. We strongly encourage code deposition in a community repository (e.g. GitHub). See the Nature Research guidelines for submitting code & software for further information.

Data

Policy information about availability of data

All manuscripts must include a data availability statement. This statement should provide the following information, where applicable:

- Accession codes, unique identifiers, or web links for publicly available datasets
- A list of figures that have associated raw data
- A description of any restrictions on data availability

All maps and fitted models that support the findings of this study are available in the EMDB and in the Protein Data Bank (PDB), respectively. Identifiers are listed in Table S5. Raw cryo-EM data are available from the corresponding author upon reasonable request. Source data are provided with this paper.

Field-specific reporting

Please select the one below that is the best fit for your research. If you are not sure, read the appropriate sections before making your selection.

☒ Life sciences ☐ Behavioural & social sciences ☐ Ecological, evolutionary & environmental sciences

For a reference copy of the document with all sections, see nature.com/documents/nr-reporting-summary-flat.pdf

Life sciences study design

All studies must disclose on these points even when the disclosure is negative.

Sample size	The sample size was not predetermined. In most cases the logarithm of the number of particle (sample size) correlates linearly with the inverse of the resolution (ResLog plots). The number of particles depend on the density of particles per micrograph, whereas the number of acquired micrographs is limited by beam time. In case the resolution was not sufficient to answer a particular research question, the number of particles was increased by acquiring more micrographs.
Data exclusions	No data were excluded from the analysis. During the analysis micrographs were sorted out by manual inspection of the CTF estimations. Individual particles were sorted out by manual inspection of 2D and 3D classes indicating particles which are damaged, overlapping or too close to the foil hole edge.
Replication	Most of the maps were created from one data set acquired from one sample. In cases where more than one data set and sample was acquired, the data was pooled to increase the number of particles and the quality (resolution) of the associated map.
Randomization	The experiments were not randomized. This is not relevant for the underlying study. No group allocation was performed.<br>Each dataset containing the particles of one type of DNA origami structure was treated individually. A 3D electron density map is created from 2D projections of thousands of particles which are assumed to be close to identical. During the reconstruction the particles are randomly assigned to two groups of equal size to be used for two independent reconstructions, which are merged after convergence.
Blinding	This is not relevant for the underlying study. No group allocation was performed (see point above).

Reporting for specific materials, systems and methods

We require information from authors about some types of materials, experimental systems and methods used in many studies. Here, indicate whether each material, system or method listed is relevant to your study. If you are not sure if a list item applies to your research, read the appropriate section before selecting a response.

Materials & experimental systems

n/a	Involved in the study
☒	☐ Antibodies
☒	☐ Eukaryotic cell lines
☒	☐ Palaeontology
☒	☐ Animals and other organisms
☒	☐ Human research participants
☒	☐ Clinical data

Methods

n/a	Involved in the study
☒	☐ ChIP-seq
☒	☐ Flow cytometry
☒	☐ MRI-based neuroimaging
