# Supplementary material for: Revealing the structures of megadalton-scale DNA complexes with nucleotide resolution
Source: Nat Commun. 2020 Dec 4;11:6229. doi: 10.1038/s41467-020-20020-7 (PMC7718922; doi:10.1038/s41467-020-20020-7)
Supplement: Supplementary file 10 — Description of Additional Supplementary Files [file 41467_2020_20020_MOESM10_ESM.pdf]

Title: Supplementary Data 1:
Descriptions: Sequences of scaffold DNA and staple oligonucleotides.

Title: Supplementary Movie 1:
Descriptions: Domain motions of the twist tower object identified via 3D classification.

Title: Supplementary Movie 2:
Descriptions: Breathing motions of the pointer v2 object identified via 3D classification, cross-sectional view,

Title: Supplementary Movie 3:
Descriptions: Breathing motions of the pointer v2 object identified via 3D classification, helical view.

Title: Supplementary Movie 4:
Descriptions: Illustration of multi-domain refinement and focussed scanning refinement

Title: Supplementary Movie 5:
Descriptions: Illustration of cascaded relaxation procedure for a region within the twist tower object.
